# Supplementary material for: Understanding the role of household hygiene practices and foodborne disease risks in child stunting: a UKRI GCRF Action Against Stunting Hub protocol paper
Source: BMJ Paediatr Open. 2024 Feb 27;8(Suppl 1):e001695. doi: 10.1136/bmjpo-2022-001695 (PMC10900323; doi:10.1136/bmjpo-2022-001695)
Supplement: Supplementary data [file bmjpo-2022-001695supp001.pdf]

APPENDIX 1: WASH AND FOOD SAFETY QUESTIONNAIRE

This questionnaire is administered by trained enumerators to mothers at 3 different time points, to elicit information about food safety and water and sanitation and hygiene practices at home, particularly in relation to infant and young child feeding. Data are collected in tablets on CommCare.

**# WATER SANITATION *(3 TIMES AT 3RD TRIMESTER, 3 & 24 MO)***
**ws_1. What is the main source of drinking water for members of your household?**
Piped waterpiped to dwelling
Piped waterpiped to compound yard or plot
Piped waterpiped to neighbor
Public tap / tapstand
Borehole or tubewell
Protected well
Unprotected well
Protected spring
Unprotected spring
Rainwater
Delivered watertanker or truck
Delivered watersmall cart with tank drum
Water kiosk
Packaged waterbottled water
Packaged watersachet water
Surface water
Other: specify
ws_1_96x. Other: specify
**ws_2. What is the main source of water used by members of this household for other purposes, such as cooking and handwashing?**
Piped waterpiped to dwelling
Piped waterpiped to compound yard or plot
Piped waterpiped to neighbor
Public tap / tapstand
Borehole or tubewell
Protected well
Unprotected well
Protected spring
Unprotected spring
Rainwater
Delivered watertanker or truck
Delivered watersmall cart with tank drum
Water kiosk
Packaged waterbottled water
Packaged watersachet water
Surface water
Other: specify
ws_2_96x. Other: specify
**ws_3. Where is this water collected from?**
In own dwelling

Dominguez-Salas P, *et al. BMJ Paediatrics Open* 2024; 8:e001695. doi: 10.1136/bmjpo-2022-001695

In own yard
Elsewhere
**ws_4. How many minutes in total does it take you to go fetch water from this source and come back? (Fill in X minutes)**
**ws_5. Do you store drinking water in your home?**
Yes
No
**ws_6. Where do you store your drinking water?**
In a container with a lid/top on it
In an open container
Container on the roof
**ws_7. In the last 4 weeks/month, how frequently did you or anyone in your household worry you would not have enough water for all of your household needs?**
Never (0 times)
Rarely (1-2 times)
Sometimes (3-10 times)
Often (11-20times)
Always (more than 20 times)
**ws_8. In the last 4 weeks/month, how frequently has your main water source been interrupted or limited (eg water pressure, less water than expected, river dried up)?**
Never (0 times)
Rarely (1-2 times)
Sometimes (3-10 times)
Often (11-20times)
Always (more than 20 times)
**ws_9. In the last 4 weeks/month, how frequently has there not been enough water to wash clothes?**
Never (0 times)
Rarely (1-2 times)
Sometimes (3-10 times)
Often (11-20times)
Always (more than 20 times)
**ws_10. In the last 4 weeks/month, how frequently have you or anyone in your household had to change schedules or plans due to problems with your water situation? Activities that may have been interrupted include caring for others, doing household chores, agricultural work, income-generating activities, sleeping, etc**
Never (0 times)
Rarely (1-2 times)
Sometimes (3-10 times)
Often (11-20times)
Always (more than 20 times)
**ws_11. In the last 4 weeks/month, how frequently have you or anyone in your household had to change what was being eaten because there were problems with water (eg, for washing foods, cooking, etc)?**
Never (0 times)
Rarely (1-2 times)
Sometimes (3-10 times)
Often (11-20times)
Always (more than 20 times)

**ws_12. In the last 4 weeks/month, how frequently have you or anyone in your household had to go without washing hands after dirty activities (eg, defecating or changing diapers, cleaning animal dung) because of problems with water?**
Never (0 times)
Rarely (1-2 times)
Sometimes (3-10 times)
Often (11-20times)
Always (more than 20 times)
**ws_13. In the last 4 weeks/month, how frequently have you or anyone in your household had to go without washing their body because of problems with water (eg, not enough water, dirty, unsafe)?**
Never (0 times)
Rarely (1-2 times)
Sometimes (3-10 times)
Often (11-20times)
Always (more than 20 times)
**ws_14. In the last 4 weeks/month, how frequently has there not been as much water to drink as you would like for you or anyone in your household?**
Never (0 times)
Rarely (1-2 times)
Sometimes (3-10 times)
Often (11-20times)
Always (more than 20 times)
**ws_15. In the last 4 weeks/month, how frequently did you or anyone in your household feel angry about your water situation?**
Never (0 times)
Rarely (1-2 times)
Sometimes (3-10 times)
Often (11-20times)
Always (more than 20 times)
**ws_16. In the last 4 weeks/month, how frequently have you or anyone in your household gone to sleep thirsty because there wasn't any water to drink?**
Never (0 times)
Rarely (1-2 times)
Sometimes (3-10 times)
Often (11-20times)
Always (more than 20 times)
**ws_17. In the last 4 weeks/month, how frequently has there been no useable or drinkable water whatsoever in your household?**
Never (0 times)
Rarely (1-2 times)
Sometimes (3-10 times)
Often (11-20times)
Always (more than 20 times)
**ws_18. In the last 4 weeks/month, how frequently have problems with water caused you or anyone in your household to feel ashamed/excluded/stigmatized?**
Never (0 times)
Rarely (1-2 times)
Sometimes (3-10 times)
Often (11-20times)

Always (more than 20 times)
**ws_19. Is there a latrine or toilet that members of this household use regularly?**
Yes
No
**ws_20. Where is this toilet facility located?**
In own dwelling
In own yard or plot
Elsewhere
**ws_21. How many households other than your own share this facility?**
Enter the enumerator Name
**fh_1. Is there a designated place for handwashing in the household?**
Yes
No
**fh_2. How often is soap available at this location?**
Always
Sometimes
Never
**fh_3. What were all of the times when you washed your hands (Circle all that apply but do not prompt After each response, ask “any other times?”)**
After defecating
After cleaning a child/wiping a child's bottom
After changing a babies nappy
Before preparing food
Before eating
Before feeding the child
After touching animals
After touching animal faeces
After handling raw animal source foods
After crop farming activities
Other
Cannot remember/does not know
fh_3_96x. Specify other
**FOOD STORAGE**
**fh_4. Do you store meat and meat products (except dried meat)?**
Yes
No
**fh_4a. Where do you store raw meat (fresh/chilled/frozen)?**
In a fridge
In a freezer
Covered in a cool room (below 5 degrees Celsius)
Uncovered in a cool room (below 5 degrees Celsius)
Covered in a room at room temperature
Uncovered in a room at room temperature
Other, specify
fh_4a_96x. Other, specify
**fh_4b. How long do you store the meat and meat products?**
less than 2 hours

2 hours to 5 hours
5 hours to 12 hours
12 hours
1-3 days
3-7 days
1 week
1 month
**fh_4c. Where do you store processed meat (ready-to-cook and ready-to-eat)?**
In a fridge
In a freezer
Covered in a cool room (below 5 degrees Celsius)
Uncovered in a cool room (below 5 degrees Celsius)
Covered in a room at room temperature
Uncovered in a room at room temperature
Other, specify
fh_4c_96x. Other, specify
**fh_4d. How long do you store the meat and meat products?**
less than 2 hours
2 hours to 5 hours
5 hours to 12 hours
12 hours
1-3 days
3-7 days
1 week
1 month
**fh_5. Do you store milk and milk products (except milk powder, evaporated milk or condensed milk)?**
Yes
No
**fh_5a. Where do you store liquid milk (including pasteurized and non-pasteurized)?**
In a fridge
In a freezer
Covered in a cool room (below 5 degrees Celsius)
Uncovered in a cool room (below 5 degrees Celsius)
Covered in a room at room temperature
Uncovered in a room at room temperature
Other, specify
fh_5a_96x. Other, specify
**fh_5b. Where do you store solid/semi solid dairy/milk products (e.g cheese, yoghurt, ghee, etc)?**
In a fridge
In a freezer
Covered in a cool room (below 5 degrees Celsius)
Uncovered in a cool room (below 5 degrees Celsius)
Covered in a room at room temperature
Uncovered in a room at room temperature
Other, specify
fh_5b_96x. Other, specify
**fh_6. Do you store raw or cooked vegetables and fruits?**

Yes
No
**fh_6a. Where do you store raw vegetables?**
In a fridge
In a freezer
Covered in a cool room (below 5 degrees Celsius)
Uncovered in a cool room (below 5 degrees Celsius)
Covered in a room at room temperature
Uncovered in a room at room temperature
Other, specify
fh_6a_96x. Other, specify
**fh_6b. Where do you store cooked vegetables and fruit?**
In a fridge
In a freezer
Covered in a cool room (below 5 degrees Celsius)
Uncovered in a cool room (below 5 degrees Celsius)
Covered in a room at room temperature
Uncovered in a room at room temperature
Other, specify
fh_6b_96x. Other, specify
**fh_7. Do you store food that has been prepared for your child to give it to the child later?**
Yes
No
**fh_7a. How do you store food that has been prepared for your child?**
Thermos
Jug
Plate or bowl (covered)
Plate or bowl (uncovered)
Cooking pot (covered)
Cooking pot (uncovered)
Other container (covered)
Other container (uncovered)
Other, specify
fh_7a_96x. Other, specify
**fh_7b. Where do you store this food?**
In a fridge
In a freezer
In a cool room (below 5 degrees Celsius)
In a room at room temperature
Other, specify
fh_7b_96x. Other, specify
**How long do you store this food?**
less than 1 hour
1-2 hours
2 hours to 5 hours
5 hours to 12 hours
12 hours

Dominguez-Salas P, *et al. BMJ Paediatrics Open* 2024; 8:e001695. doi: 10.1136/bmjpo-2022-001695

1-3 days
3-7 days
Longer than a week
Animals
**fh_8. Does your household keep any animals inside or outside the house you are looking after (i.e. not taken care of by others)?**
Yes
No
**fh_9. What type of animals?**
Chickens
Ducks
Other birds
Dogs
Cats
Goats
Sheep
Pigs
Cows
Fish
Other animals, specify
fh_9_96x. Other: specify
**fh_10. Which of these animals are in the patio, veranda or house any time of the day?**
None
Chickens
Ducks
Other birds
Dogs
Cats
Goats
Sheeps
Pigs
Cows
Other animals, specify
fh_10_96x. Other: specify
**fh_10a. Is the animal contained in some form of enclosure?**
Yes
No
**fh_10b. Can the child be in contact with the animals or their faeces?**
Yes
No
**fh_10c. Can the animal be in contact with food for the child?**
Yes
No
**ASF1_1. What is the first priority ASF consumed by the child most recently? Select [ASF1]**
Chicken
fish
beef
egg

squid
meat ball
beef soup
chicken soup
duck egg
fish egg
None
**ASF1_2. How was this [ASF] packaged when you got it from the source? (select all that apply)**
No packaging
Sealed package
Washable container (plastic, glass, steel), open
Washable container (plastic, glass, steel), closed
Plastic bag
Newspaper
Other paper
Aluminium foil
Other foil
Container made from natural materials
Other, specify
ASF1_2_96x. ASF_2_96x Other: specify
**ASF1_3. Did you transport this [ASF] after you got it?**<br>**No transport means that they either produced it themselves or the ASF was brought to them (e.g. by a trader or neighbour, etc)**
Yes
No
**ASF1_3a. How long did you transport the ASF for (to take it home)?**
less than 5 minutes
5 min to 15 min
15 to 30 min
30 min to 1 hour
1hour to 2 hours
2 hours to 3 hours
3 hours
**ASF1_3b. How did you transport this [ASF] after you got it?**
Chilled (e.g. on ice)
At ambient temperature
Kept warm (e.g. thermos or thermal food container)
Other, specify
ASF1_3b_96x. Other, specify
**ASF1_4. Did you store this [ASF] before preparation?**
Yes
No
**ASF1_4a. Was this [ASF] covered when you stored it before preparation?**
Yes
No
**ASF1_4b. Where did you store this [ASF] before preparation?**
In a fridge
In a freezer

In a cool room (below 5 degrees Celsius)
In a room at room temperature
Other, specify
ASF1_4b_96x. Specify, other
**ASF1_4c. How long was this [ASF] stored before preparing?**
minute
hour
day
total minutes
total hours
total day
**ASF1_5. Did you wash this [ASF] before preparation?**
Yes
No
Not applicable
**ASF1_6. If yes, why did you wash this [ASF]? (multiple answers)**
To remove visible dirt
To remove visible blood
To make it safer
Because it is a good practice
Other, specify
ASF1_6_96x. Specify, other
**ASF1_7. Did you chop or cut this [ASF] product before preparation?**
Yes
No
Not applicable
**ASF1_8. If yes, which tool(s) did you use to chop or cut this [ASF]? (multiple answers)**
Cutting board
Plate
Knife
Other, specify
ASF1_8_96x. Specify, other
**ASF1_9. How did you prepare this food for the child?**
Grilling
Baking
Frying
Boiling
Stewing
Simmering
Fermenting
Heating
Mixed in with other ingredients without cooking the ASF (e.g. mixing yoghurt with fruit or adding dried fish to salad)
Made smaller portion (e.g. cutting in pieces or butting in a bowl), but did not cook or mix with other ingredients
Other, specify
ASF1_9_96x. Specify, other
**ASF1_10. For how long was this [ASF] cooked?**

1-5 min
5-15 min
15-30 min
30-60 min
More than 1 hour
**ASF1_11. After preparation, was this [ASF] stored before giving it to the child?**
Yes
No
**ASF1_12. After preparation, how long was this [ASF] stored before giving it to the child?**
1-5 min
5-15 min
15-30 min
30-60 min
More than 1 hour, less than 24h
More than 1 day, less than 2 days
More than 2 days
**ASF1_13. How did you store this [ASF] that had been prepared for your child?**
Thermos
Jug
Plate or bowl (covered)
Plate or bowl (uncovered)
Cooking pot (covered)
Cooking pot (uncovered)
Other container (covered)
Other container (uncovered)
Other, specify
ASF1_13_96x. Specify, other
**ASF1_14. Where did you store this [ASF]?**
In a fridge
In a freezer
In a cool room (below 5 degrees Celsius)
In a room at room temperature
Other, specify
ASF1_14_96x. Specify other
**ASF2_1. What is the second priority ASF consumed by the child most recently? Select [ASF1]**
Chicken
fish
beef
egg
squid
meat ball
beef soup
chicken soup
duck egg
fish egg
None
**ASF2_2. How was this [ASF] packaged when you got it from the source? (select all that apply)**

No packaging
Sealed package
Washable container (plastic, glass, steel), open
Washable container (plastic, glass, steel), closed
Plastic bag
Newspaper
Other paper
Aluminium foil
Other foil
Container made from natural materials
Other, specify
ASF_2_96x Other: specify
**ASF2_3. Did you transport this [ASF] after you got it?**<br>**No transport means that they either produced it themselves or the ASF was brought to them (e.g. by a trader or neighbour, etc)**
Yes
No
**ASF2_3a. How long did you transport the ASF for (to take it home)?**
less than 5 minutes
5 min to 15 min
15 to 30 min
30 min to 1 hour
1hour to 2 hours
2 hours to 3 hours
3 hours
**ASF2_3b. How did you transport this [ASF] after you got it?**
Chilled (e.g. on ice)
At ambient temperature
Kept warm (e.g. thermos or thermal food container)
Other, specify
ASF2_3b_96x. Other specify
**ASF2_4. Did you store this [ASF] before preparation?**
Yes
No
**ASF2_4a. Was this [ASF] covered when you stored it before preparation?**
Yes
No
**ASF2_4b. Where did you store this [ASF] before preparation?**
In a fridge
In a freezer
In a cool room (below 5 degrees Celsius)
In a room at room temperature
Other, specify
ASF2_4b_96x. Specify, other
**ASF2_4c. How long was this [ASF] stored before preparing?**
minute
hour
day

total minutes
total hours
total day
**ASF2_5. Did you wash this [ASF] before preparation?**
Yes
No
Not applicable
**ASF2_6. If yes, why did you wash this [ASF]? (multiple answers)**
To remove visible dirt
To remove visible blood
To make it safer
Because it is a good practice
Other, specify
ASF2_6_96x. Specify, other
**ASF2_7. Did you chop or cut this [ASF] product before preparation?**
Yes
No
Not applicable
**ASF2_8. If yes, which tool(s) did you use to chop or cut this [ASF]? (multiple answers)**
Cutting board
Plate
Knife
Other, specify
ASF2_8_96x. Specify, other
**ASF2_9. How did you prepare this food for the child?**
Grilling
Baking
Frying
Boiling
Stewing
Simmering
Fermenting
Heating
Mixed in with other ingredients without cooking the ASF (e.g. mixing yoghurt with fruit or adding dried fish to salad)
Made smaller portion (e.g. cutting in pieces or butting in a bowl), but did not cook or mix with other ingredients
Other, specify
ASF2_9_96x. Specify, other
**ASF2_10. For how long was this [ASF] cooked?**
1-5 min
5-15 min
15-30 min
30-60 min
More than 1 hour
**ASF2_11. After preparation, was this [ASF] stored before giving it to the child?**
Yes
No

**ASF2_12. After preparation, how long was this [ASF] stored before giving it to the child?**
1-5 min
5-15 min
15-30 min
30-60 min
More than 1 hour, less than 24h
More than 1 day, less than 2 days
More than 2 days
**ASF2_13. How did you store this [ASF] that had been prepared for your child?**
Thermos
Jug
Plate or bowl (covered)
Plate or bowl (uncovered)
Cooking pot (covered)
Cooking pot (uncovered)
Other container (covered)
Other container (uncovered)
Other, specify
ASF2_13_96x. Specify, other
**ASF2_14. Where did you store this [ASF]?**
In a fridge
In a freezer
In a cool room (below 5 degrees Celsius)
In a room at room temperature
Other, specify
ASF2_14_96x. Specify, other
**ASF3_1. What is the third priority ASF consumed by the child most recently? Select [ASF1]**
Chicken
fish
beef
egg
squid
meat ball
beef soup
chicken soup
duck egg
fish egg
None
**ASF3_2. How was this [ASF] packaged when you got it from the source? (select all that apply)**
No packaging
Sealed package
Washable container (plastic, glass, steel), open
Washable container (plastic, glass, steel), closed
Plastic bag
Newspaper
Other paper
Aluminium foil

Other foil
Container made from natural materials
Other, specify
ASF_2_96x Other: specify
**ASF3_3. Did you transport this [ASF] after you got it?**<br>**No transport means that they either produced it themselves or the ASF was brought to them (e.g. by a trader or neighbour, etc)**
Yes
No
**ASF3_3a. How long did you transport the ASF for (to take it home)?**
less than 5 minutes
5 min to 15 min
15 to 30 min
30 min to 1 hour
1hour to 2 hours
2 hours to 3 hours
3 hours
**ASF3_3b. How did you transport this [ASF] after you got it?**
Chilled (e.g. on ice)
At ambient temperature
Kept warm (e.g. thermos or thermal food container)
Other, specify
ASF3_3b_96x. Other, specify
**ASF3_4. Did you store this [ASF] before preparation?**
Yes
No
**ASF3_4a. Was this [ASF] covered when you stored it before preparation?**
Yes
No
**ASF3_4b. Where did you store this [ASF] before preparation?**
In a fridge
In a freezer
In a cool room (below 5 degrees Celsius)
In a room at room temperature
Other, specify
ASF3_4b_96x. Specify, other
**ASF3_4c. How long was this [ASF] stored before preparing?**
minutes
hour
day
total minutes
total hours
total day
**ASF3_5. Did you wash this [ASF] before preparation?**
Yes
No
Not applicable
**ASF3_6. If yes, why did you wash this [ASF]? (multiple answers)**

To remove visible dirt
To remove visible blood
To make it safer
Because it is a good practice
Other, specify
ASF3_6_96x. Specify, other
**ASF3_7. Did you chop or cut this [ASF] product before preparation?**
Yes
No
Not applicable
**ASF3_8. If yes, which tool(s) did you use to chop or cut this [ASF]? (multiple answers)**
Cutting board
Plate
Knife
Other, specify
ASF3_8_96x. Specify, other
**ASF3_9. How did you prepare this food for the child?**
Grilling
Baking
Frying
Boiling
Stewing
Simmering
Fermenting
Heating
Mixed in with other ingredients without cooking the ASF (e.g. mixing yoghurt with fruit or adding dried fish to salad)
Made smaller portion (e.g. cutting in pieces or butting in a bowl), but did not cook or mix with other ingredients
Other, specify
ASF3_9_96x. Specify, other
**ASF3_10. For how long was this [ASF] cooked?**
1-5 min
5-15 min
15-30 min
30-60 min
More than 1 hour
**ASF3_11. After preparation, was this [ASF] stored before giving it to the child?**
Yes
No
**ASF3_12. After preparation, how long was this [ASF] stored before giving it to the child?**
1-5 min
5-15 min
15-30 min
30-60 min
More than 1 hour, less than 24h
More than 1 day, less than 2 days
More than 2 days

**ASF3_13. How did you store this [ASF] that had been prepared for your child?**
Thermos
Jug
Plate or bowl (covered)
Plate or bowl (uncovered)
Cooking pot (covered)
Cooking pot (uncovered)
Other container (covered)
Other container (uncovered)
Other, specify
ASF3_13_96x. Specify, other
**ASF3_14. Where did you store this [ASF]?**
In a fridge
In a freezer
In a cool room (below 5 degrees Celsius)
In a room at room temperature
Other, specify
ASF3_14_96x. Other, specify

APPENDIX 2: WASH AND FOOD SAFETY SPOT CHECK

This checklist is filled by trained enumerators in the households at 3 different time points, to observe and record information about behaviours and facilities in relation to food safety and water and sanitation and hygiene practices at home. Data are collected in tablets on CommCare.

WATER SANITATION (SPOT CHECK) *(3 TIMES AT 2ND TRIMESTER, 3 & 9MO)*
**wash_1. What type of facility is the primary location for defecation?**
Flush toilet to piped sewer system
Flush toilet to septic tank
Flush toilet to pit
Flush toilet to onsite, above ground, open pit
Pour flush toilet to piped sewer system
Pour flush toilet to septic tank
Pour flush toilet to pit
Pour flush toilet to onsite, above ground, open pit
Pit latrine with concrete slab (not pour flush)
Pit latrine without slab (not pour flush)
Bucket
Bag
Open defecation (no facilities)
Other:
Refused observation
wash_1_96x. Specify, other
**wash_2. Approximately how many meters is the toilet from the entrance of the household**
**wash_3. For the following, please note if following indicators of functionality are present**
**wash_3_1. Is the pan working (not broken)**
yes
no
**wash_3_2. Are there leaves or rubbish blocking the pan?**
yes
no
**wash_3_3. Is there standing water in the pan?**
yes
no
**wash_3_4. Is there a cover over the pit?**
yes
no
**wash_3_5. Are there one or two pits?**
One
Two
More
**wash_3_6. Is there a cover on all the pits?**
No
Partial (only to one pit)
Yes, to more than one pit
**wash_3_7. Are there flies?**

None
Some
Many
**wash_4. Can you please show me where members of this household most often wash their hands?**
Fixed facility (sink/basin)… in dwelling
Fixed facility….in yard
Mobile object (bucket / jug)
No handwashing place in dwelling
Refused observation
**wash_5. Is there water available at this location?**
Yes
No
**wash_6. Is soap available at this location? (Soap includes bar soap, liquid soap, powder detergent, and soapy water but does not include ash, soil, sand or other handwashing agents)**
Yes
No
**wash_7. Is the dedicated location located near (within 5 steps) the primary cooking location?**
Yes
No
**wash_8. Is the dedicated location located near (within 5 steps) the latrine/toilet**
Yes
No
**wash_9. Are there animals located in or around the household? (select all)**
No animals visible
In the cooking area (outside house)
In the cooking area (inside house)
In the food storing area (outside house)
In the food storing area (inside house)
In other areas of the house that are not cooking or food storing areas
**wash_10. What type of animals (select all)**
Chickens
Ducks
Other birds
Dog
Cat
Goat
Sheep
Pig
Cow
Wild animals
Rodents
Other animal
wash_10_96x. Other, specify
**wash_11. Are there animal feces located in or around the household? (select all)**
No animal feces visible
In the cooking area (outside house)
In the cooking area (inside house)
In the food storing area (outside house)

In the food storing area (inside house)
In other areas of the house that are not cooking or food storing areas
In the yard / courtyard
FOOD SAFETY QUESTIONNAIRE *(1 TIME: AT 6 MO)*
HAND WASHING
**spot_1. Can you please show me where members of this household most often wash their hands after using the toilets?**
Fixed facility (sink/basin)… in dwelling
Fixed facility….in yard
Mobile object (bucket / jug)
No handwashing place in dwelling
Refused observation
**spot_2. Is there water available at this location?**
Yes
No
**spot_3. Is soap available at this location? (Soap includes bar soap, liquid soap, powder detergent, and soapy water but does not include ash, soil, sand or other handwashing agents)**
Yes
No
**spot_4. Is the dedicated location located near (within 5 steps) the primary cooking location?**
Yes
No
**spot_5. Is the dedicated location located near (within 5 steps) the latrine/toilet**
Yes
No
**spot_6. Can you show me where members of this household most often wash their hands before eating or preparing food?**
Fixed facility (sink/basin)… in dwelling
Fixed facility….in yard
Mobile object (bucket / jug)
No handwashing place in dwelling
Refused observation
Same as after toilet
**spot_7. Is there water available at this location?**
Yes
No
**spot_8. Is soap available at this location? (Soap includes bar soap, liquid soap, powder detergent, and soapy water but does not include ash, soil, sand or other handwashing agents)**
Yes
No
**spot_9. Is the dedicated location located near (within 5 steps) the primary cooking location?**
Yes
No
**spot_10. Is the dedicated location located near (within 5 steps) the latrine/toilet**
Yes
No
**spot_11. Do you see animals in or around the household? (select all)**

No animals visible
In the cooking area (outside house)
In the cooking area (inside house)
In the food storing area (outside house)
In the food storing area (inside house)
In other areas of the house that are not cooking or food storing areas
**spot_12. What type of animals (select all)**
Chickens
Ducks
Other birds
Dog
Cat
Goat
Sheep
Pig
Cow
Wild animals
Rodents
Other animal
spot_12_96x. Specify other
**spot_13. Is it possible for the child to be in contact with these animals (e.g. if they are unrestrained or the child can access an enclosure)?**
Yes
No
**spot_14. Is it possible for these animals to be in contact with food (e.g. if animals are unrestrained)**
Yes
No
**spot_15. Are there visible animal feces located in or around the household? (select all)**
No animal feces visible
In the cooking area (outside house)
In the cooking are (inside house)
In the food storing area (outside house)
In the food storing area (inside house)
In other areas of the house that are not cooking or food storing areas
In the yard / courtyard
In the animal enclosure (e.g. chicken coop)
**spot_16. Is it possible for the child to be in contact with these faeces (e.g. because they are in areas where the child is or the child can access an enclosure)?**
Yes
No
**spot_17. Is it possible that food gets in contact with these faeces?**
Yes
No
**safe_1. Is there a functioning refrigerator?**
Yes
No
**safe_2. Is there a functioning freezer?**
Yes

No
**safe_3. Is there any visible, stored [ASF] not protected from the environment?**
Yes
No
**safe_4. Is it [ASF] in contact with other food products?**
Yes
No
**safe_5. Is the area where this [ASF] is kept protected from animals and flies?**
Yes
No
**safe_6. Is there a kitchen area (a dedicated food preparation area)?**
Yes
No
**safe_6a. Is the kitchen area free from pest animals (i.e. birds, flies, rats)**
Yes
No
**safe_6b. Are there anti-pest measures in place in the kitchen area? e.g. nets, traps, insect killers**
Yes
No
**safe_6c. Is the kitchen area free of visible dirt?**
Yes
No
**safe_6d. Are cooking utensils clean?**
Yes
No
**safe_6e. Is the kitchen area easy to clean (e.g. tiles)?**
Yes
No
**safe_6f. Does the kitchen area have access to running water?**
Yes
No
**safe_6g. If there is no access to running water, is there stored water in the area where food is being prepared?**
Yes, there is water in a container with a lid/top on it
Yes, there is water in an open container
No, there is no water
**safe_6h. Is soap available at this location? (Soap includes bar soap, liquid soap, powder detergent, and soapy water but does not include ash, soil, sand or other handwashing agents)**
Yes
No
**safe_7. Is there a food storage area?**
Yes
No
**safe_7a. In the food storage area, is the storage equipment made of material that is easy to clean (e.g. metal, plastic)?**
Yes
No
**safe_7b. Is the food storage area free from pest animals (i.e. birds, flies, rats)**

Yes
No
**safe_7c. Are there anti-pest measures in place in the food storage area? e.g. nets, traps, insect killers**
Yes
No
**safe_7d. Is the food storage area free of visible dirt?**
Yes
No
**safe_7e. Is the food storage area easy to clean (e.g. tiles)?**
Yes
No
**safe_8. Is there a facility to dispose of food waste?**
No
In the cooking area (outside house)
In the cooking area (inside house)
In the food storing area (outside house)
In the food storing area (inside house)
Outside the house remove from cooking or food storing areas
Removed from the household
**safe_9. Are the knife and chopping board used to cut vegetables and fruits are clean?**
Yes
No
**safe_10.Are the nails of the food handler trimmed?**
Yes
No

Dominguez-Salas P, et al. BMJ Paediatrics Open 2024; 8:e001695. doi: 10.1136/bmjpo-2022-001695

APPENDIX 3: WASH AND FOOD SAFETY DIRECT OBSERVATION TOOL

This direct observation tool is filled by trained enumerators in the households at 3 different time points, to observe and record information about behaviours related to food safety and water and sanitation and hygiene practices at home, particularly in relation to infant and young child feeding. Food, drinking water and surface samples are subsequently collected for microbiological testing. Data are collected on paper.

GCRF Action against stunting Hub

**Household ID:**	**Mother ID:**	**Child ID:**
**Enumerator ID:**	**Supervisor ID:**	
**District:**	**Sub-county:**	
**Parish:**	**Village:**	
**Salter calibration ID:**	**Salter calibration weight:**	
**Date of record:**<br><br>**Time ARRIVED:** *(24-hr)*<br><br>**Time DEPARTED:** *(24-hr)*	**Day of the week** **today**:<br><br>Monday ☐ Friday ☐<br>Tuesday ☐ Saturday ☐<br>Wednesday ☐ Sunday ☐<br>Thursday ☐	
Complete at the end of the day		

Describe the location of the observations: **Where in the home the observation was completed – where the observer sat, what the conditions were when you did the observation (weather, lighting, heat) how busy the home was, etc.**

Describe who the main caretaker of the child was:

Mother ☐
Sibling ☐
Grandmother ☐
Aunt ☐
Caretaker ☐
Other, specify ☐ ____

Please note if caretaker changes during observation: Yes ☐ **No** ☐ **Mother** ☐
Sibling ☐
Grandmother ☐
Aunt ☐
Caretaker ☐
Other, specify ☐ ____

Dominguez-Salas P, *et al. BMJ Paediatrics Open* 2024; 8:e001695. doi: 10.1136/bmjpo-2022-001695

Direct observation

Household ID: _____

	Yes *(tick)*	Date: *dd/mm/yy*
**Checked by supervisor:** *(code)*		
**Checked by co-ordinator:** *(code)*		
**Data entry completed:** *(code)*		

Activity	___ ____:00				____ ____:00				___ ____:00				____ ____:00				Pilot notes
	a	b	c	d	e	f	g	h	I	j	k	l	m	n	o	p	
	00	15	30	45	00	15	30	45	00	15	30	45	00	15	30	45	
1. Child Activities																	
1.1 Sleeping																	
1.2 Caretaker change																	
1.3 Child being cared for by other children																	
2. Child feces																	
2.1 Child nappy or diaper is changed																	
2.2 Child defecates on floor																	
2.3 Child defecates in potty																	
2.4 Child feces disposed in toilet																	
2.5 Child feces disposed in bush / ditch																	
3. Food preparation																	
3.1 Fruit or vegetable washed																	
3.2 Food preparation surface rinsed (specify surface type)																	
3.3 Food preparation surface washed (specify surface type)																	
3.4 Utensils rinsed																	
3.5 Utensils washed																	
3.6 Plates / bowls/ cups rinsed																	
3.7 Plates / bowls/cups washed																	
3.8 Child b ottle rinsed																	
3.9 Child b ottle cleaned																	
4. Food cooking																	Specify from section 3

Dominguez-Salas P, *et al. BMJ Paediatrics Open* 2024; 8:e001695. doi: 10.1136/bmjpo-2022-001695

Direct observation

Household ID: _____

Activity	___ ____:00				____ ____:00				___ ____:00				____ ____:00				Pilot notes
	a	b	c	d	e	f	g	h	I	j	k	l	m	n	o	p	
	00	15	30	45	00	15	30	45	00	15	30	45	00	15	30	45	
																	Specify type in comments
4.1 Food fried (ASF / Fruit Veg / Other)																	
4.2 Food dried (ASF / Fruit Veg / other)																	
4.3 Food roasted (ASF / Fruit Veg / other)																	
4.4 Food heated in pot, no boiled (ASF/ Fruit Veg / other)																	
4.5 Food boiled (ASF / Fruit Veg/ other)																	
4.6 Food steamed (ASF / Fruit Veg / Other)																	
4.7 Food arrives processed (ASF / Fruit Veg / Other)																	
4.8 Food added raw / uncooked to dish (ASF / Fruit Veg / Other)																	
5. Food handling																	Specify from section 4
5.1 Cooked food served immediately to child																	
5.2 Cooked food left at room temp / open																	
5.3 Cooked food placed in cabinet / cupboard																	
5.4 Cooked food placed in fridge																	
5.5 Cooked food covered (loosely)																	
5.6 Cooked food covered (sealed)																	
6. Child feeding																	Specify from section 4
6.1 Child breastfed																	
6.2 Child given food that was stored																	
6.3 Child given milk																	
6.4 Child given water																	
6.5 Flies on food given to child																	
6.6 Child fed by caregivers hand																	
6.6 Child eating using child’s hand																	
6.7 Child fed/eating using utensils (e.g. spoon)																	
7. Floor contact																	

Direct observation

Household ID: _____

Activity	___ ____:00				____ ____:00				___ ____:00				____ ____:00				Pilot notes
	a	b	c	d	e	f	g	h	I	j	k	l	m	n	o	p	
	00	15	30	45	00	15	30	45	00	15	30	45	00	15	30	45	
7.1 Child hand contact with floor																	
7.2 Child hand contact with dirt																	
7.3 Child eats dirt / soil																	
7.4 Child places objects from the floor in their mouths																	
7.5 Child places hands that have been in contact with the floor in mouth																	
8. Animal contact																	Write down the animal (chickens, ducks, other birds, goats, sheep, pigs, cow, dog, cat, other/specify)
8.1 Caretaker hand contact with animals																	
8.2 Caretaker hand contact with animal feces																	
8.3 Child hand contact with animals																	
8.4 Child hand contact with animal feces																	
9. Toilet use																	
9.1 Primary caretaker uses toilet																	
9.2 Primary caretaker goes for open defecation																	
10. Domestic cleaning																	
10.1 Caretakers washes clothes																	
10.2 Caretaker / other household member sweeps																	
10.3 Caretaker / other household members cleans floors (with disinfectant / soap)																	
10.3 Caretakers handles garbage																	
11. Handwashing / Facewashing																	
11.1 Caretaker rines hands – water only																	
11.2 Caretaker washes hands – soap used																	
11.3 Child's hands rinsed – water only																	
11.4 Child's hands washed – soap used																	
11.5 Child's face wiped or rinses																	
11.6 Child's face cleaned																	
11.7 Caretaker bathes																	

Direct observation

Household ID: _____

Activity	___ ____:00				____ ____:00				___ ____:00				____ ____:00				Pilot notes
	a	b	c	d	e	f	g	h	I	j	k	l	m	n	o	p	
	00	15	30	45	00	15	30	45	00	15	30	45	00	15	30	45	
11.8 Child bathed																	

Direct observation
Household ID: _____

Data log Table. Food Sample

HH ID: _________________________
Timepoint: 9 month/ 12 month/ 18 month
Date (DD/MMM/YY): _________________________
Collection time (24 hour clock, HH:MM): ---_________________________

Collection period: During feeding ☐ Directly after feeding ☐ Before feeding ☐	
Food:	
Total sample weight	
Ingredients:	
Preparation method:	
Notes:	

Direct observation

Household ID: _____

Data log Table. Water Sample

HH ID: _________________________

Timepoint: 9 month/ 12 month/ 18 month

Date (DD/MMM/YY): _________________________

Collection time (24 hour clock, HH:MM): ---_________________________

Has the water been treated with chlorine/aquatabs or is from the municipal system?<br>Yes ☐ (collect water in a small 100 mL whirlpak bag with sodium thiosulfate tablets + collect further water in a 50mL centrifuge tube)<br>No ☐ (collect water in a large 250 mL whirlpak bag)	
Notes:	

Direct observation
Household ID: _____

Data log Table. Surface Sample

Timepoint: 9 month/ 12 month/ 18 month
HH ID: _________________________
Date (DD/MMM/YY): _________________________
Collection time (24 hour clock, HH:MM): ---_________________________

Where is the child's food prepared [the surface where foods and utensils are placed during preparation and/or where foods cut up or otherwise processed in any way]?	
Notes:	
