# Supplementary material for: Understanding the role of household hygiene practices and foodborne disease risks in child stunting: a UKRI GCRF Action Against Stunting Hub protocol paper
Source: BMJ Paediatr Open. 2024 Feb 27;8(Suppl 1):e001695. doi: 10.1136/bmjpo-2022-001695 (PMC10900323; doi:10.1136/bmjpo-2022-001695)
Supplement: Author's manuscript [file bmjpo-2022-001695.draft_revisions.pdf]

BMJ Paediatrics Open

BMJ Paediatrics Open is committed to open peer review. As part of this commitment we make the peer review history of every article we publish publicly available.

When an article is published we post the peer reviewers' comments and the authors' responses online. We also post the versions of the paper that were used during peer review. These are the versions that the peer review comments apply to.

The versions of the paper that follow are the versions that were submitted during the peer review process. They are not the versions of record or the final published versions. They should not be cited or distributed as the published version of this manuscript.

BMJ Paediatrics Open is an open access journal and the full, final, typeset and author-corrected version of record of the manuscript is available on our site with no access controls, subscription charges or pay-per-view fees (http://bmjpaedsopen.bmj.com).

If you have any questions on BMJ Paediatrics Open's open peer review process please email info.bmjpo@bmj.com

BMJ Paediatrics Open

Understanding the role of household hygiene practices and foodborne disease risks in child stunting: a UKRI GCRF Action Against Stunting Hub protocol paper

Journal:	*BMJ Paediatrics Open*
Manuscript ID	bmjpo-2022-001695
Article Type:	Protocol
Date Submitted by the Author:	04-Oct-2022
Complete List of Authors:	Dominguez-Salas, Paula; University of Greenwich; International Livestock Research Institute (ILRI)<br>Waddington, Hugh Sharma; London School of Hygiene and Tropical Medicine, Department of Disease Control<br>Grace, Delia; University of Greenwich; International Livestock Research Institute (ILRI)<br>Bosire, Caroline; International Livestock Research Institute (ILRI)<br>Moodley, Arshnee; International Livestock Research Institute (ILRI)<br>Kulkarni, Bharati; National Institute of Nutrition<br>Dasi, Teena; National Institute of Nutrition<br>Kumar, Santosh; National Institute of Nutrition, Clinical Division<br>Kumar, Ramachandrappa Naveen; National Institute of Nutrition<br>Fahmida, Umi; Southeast Asian Ministry of Education Organisation Regional Centre for Food and Nutrition (SEAMEO RECFON)<br>Htet, Min Kyaw; Southeast Asian Ministry of Education Organisation Regional Centre for Food and Nutrition (SEAMEO RECFON)<br>Sudibya, Arienta R.P.; Southeast Asian Ministry of Education Organisation Regional Centre for Food and Nutrition (SEAMEO RECFON)<br>Faye, Babacar; Université Cheikh Anta Diop (UCAD)<br>Tine, Roger ; University of Cheikh Anta DIOP, Department of Parasitology-Mycology<br>Saxena, Deepak ; Public Health Foundation of India<br>Dreibelbis, Robert; London School of Hygiene and Tropical Medicine, Department of Disease Control<br>Häsler, Barbara N; Royal Veterinary College (RVC), Department of Pathobiology and Population Sciences
Keywords:	Epidemiology, Microbiology, Data Collection

SCHOLARONE™
Manuscripts

I, the Submitting Author has the right to grant and does grant on behalf of all authors of the Work (as defined in the below author licence), an exclusive licence and/or a non-exclusive licence for contributions from authors who are: i) UK Crown employees; ii) where BMJ has agreed a CC-BY licence shall apply, and/or iii) in accordance with the terms applicable for US Federal Government officers or employees acting as part of their official duties; on a worldwide, perpetual, irrevocable, royalty-free basis to BMJ Publishing Group Ltd ("BMJ") its licensees and where the relevant Journal is co-owned by BMJ to the co-owners of the Journal, to publish the Work in this journal and any other BMJ products and to exploit all rights, as set out in our licence.

The Submitting Author accepts and understands that any supply made under these terms is made by BMJ to the Submitting Author unless you are acting as an employee on behalf of your employer or a postgraduate student of an affiliated institution which is paying any applicable article publishing charge ("APC") for Open Access articles. Where the Submitting Author wishes to make the Work available on an Open Access basis (and intends to pay the relevant APC), the terms of reuse of such Open Access shall be governed by a Creative Commons licence – details of these licences and which Creative Commons licence will apply to this Work are set out in our licence referred to above.

Other than as permitted in any relevant BMJ Author's Self Archiving Policies, I confirm this Work has not been accepted for publication elsewhere, is not being considered for publication elsewhere and does not duplicate material already published. I confirm all authors consent to publication of this Work and authorise the granting of this licence.

Confidential: For Review Only

Understanding the role of household hygiene practices and foodborne disease risks in child stunting: a UKRI GCRF Action Against Stunting Hub protocol paper

Paula Dominguez-Salas[ab], Hugh Sharma Waddington[c], Delia Grace[ab], Caroline Bosire[b], Arshnee Moodley[b], Bharati Kulkarni[d], Teena Raajev[d], Santosh Kumar Banjara[d], Ramachandrappa Naveen Kumar [d], Umi Fahmida[e], Kyaw Htet[e], Arienta R.P. Sudibya[e], Babacar Faye[f], Roger C. Tine[f], Deepak Saxena[g], Robert Dreibelbis[c], Barbara Häsler[h]

[a]Natural Resources Institute (NRI), University of Greenwich, London, United Kingdom

[b]International Livestock Research Institute (ILRI), Nairobi, Kenya

[c]Environmental Health Group, Department of Disease Control, London School of Hygiene and Tropical Medicine, London, United Kingdom

[d]ICMR- National Institute of Nutrition (ICMR), Hyderabad, Telangana, India

[e]Southeast Asian Ministers of Education Organization Regional Center for Food and Nutrition (SEAMEO-RECFON)-Pusat Kajian Gizi Regional, Universitas Indonesia, Jakarta, Indonesia

[f]Department of Parasitology-Mycology, University of Cheikh Anta DIOP, Dakar, Senegal

[g]Indian Institute of Public Health Gandhinagar (IIPHG), India

[h]Department of Pathobiology and Population Sciences, Royal Veterinary College (RVC), Hatfield, United Kingdom

Corresponding author:

Paula Dominguez Salas, Natural Resources Institute, University of Greenwich, Chatham Maritime, Kent, United Kingdom, P.DominguezSalas@gre.ac.uk

Keywords: food safety, hygiene, water, sanitation, child stunting, protocol

Confidential: For Review Only

ABSTRACT

Introduction: Environmental hygiene and food safety are important determinants of child stunting. This research aims to explore the relationship between child stunting and household hygiene practices and behaviours, including the availability of water, sanitation and hygiene (WASH) facilities; the use of safe food and good quality drinking water (especially when used for complementary feeding); hygienic practices in food transport, storage and preparation; and the control of cross-contamination from animals, their produce and waste.

Methods and analysis: This study is part of a wider observational study which aims to investigate the interdisciplinary factors contributing to child stunting using a 'whole child' paradigm. The observational study recruits women during pregnancy in Hyderabad, India, Lombok, Indonesia and Kaffrine, Senegal, and dyads (i.e., 500 mother-infant pairs per country) are followed longitudinally up to 24 months after birth. Within the interdisciplinary niche, the study herein has developed tools to investigate the potential exposure pathways to environmental pathogen contamination of foods and water. Holistic WASH and food safety data collection tools have been developed to explore exposure pathways at the household-level, including: i) survey questionnaires; ii) spot-checks; iii) biological sampling of drinking water, food and domestic surfaces; and iv) direct observation. An integrated analytical approach will be used to triangulate the evidence in order to examine the relationships between child stunting, WASH and food safety behaviours.

Ethics and dissemination: Ethical approval of the study was granted by the ethics committee of the LSHTM, RVC, ILRI, ICMR, IIPHG, SEAMEO-RECFON, University of Cheikh Anta Diop. Findings of the study will be disseminated through publication in peer-reviewed journals, relevant international conferences, public engagement events, and policy-maker and stakeholder events.

Confidential: For Review Only

SUMMARY BOX

- **What is known about the subject**
  - From the moment complementary feeding begins, the chances of exposure to food contamination and infectious disease increase exponentially
  - Adequate WASH and food safety practices can play a key role in reducing infectious disease transmission and child malnutrition.
  - The contribution of hygiene behaviours and food contamination in driving transmission of infections and childhood stunting requires further investigation.

- **What this study hopes to add**
  - Development, application and analysis of integrated WASH and food safety tools, towards a holistic understanding of how these practices and behaviours impact child stunting
  - Evidence towards a new typology of child stunting, with a focus on exposure pathways for pathogens, and to support programmes and policies to minimise stunting in childhood.

Confidential: For Review Only

INTRODUCTION

The incidence and severity of stunting in childhood is closely linked to exposure to infection from food, drinking water and the wider environment[1]. Infections associated with contaminated water or insufficient water, sanitation and hygiene (WASH) are responsible for an estimated 21% of the total global burden of diseases[2], contributing to the outbreak and chronicity of preventable infections such as diarrhoeal diseases and ARI (Acute Respiratory Infections), which are the two leading causes of death in children globally[3]. In addition, the World Health Organization (WHO) Foodborne Disease Burden Epidemiology Reference Group (FERG), estimated that approximately 40% of the global burden of foodborne diseases occurs in children under the age of five years[4]. A common cause of foodborne infections is the consumption of raw or undercooked meat, fish, seafood, eggs, fresh produce and dairy products contaminated by norovirus, Campylobacter, non-typhoidal Salmonella or pathogenic *E. coli*[4]. Food hygiene is a primary concern in the preparation of complementary foods - for example, bottles often cannot be adequately sterilised and perishable complementary foods are often left unrefrigerated or eaten using unclean utensils or with unwashed hands.

Disease transmission from faeces can be food- or water-borne (i.e., oral ingestion of contaminated food and water), water-washed or water-scarce (i.e., spread through inadequate hand and food hygiene) and water-based (i.e., transmitted by parasites that penetrate skin in water, such as schistosomiasis, or by walking barefoot on contaminated soil in the case of hookworm)[5]. For example, theory and evidence suggests that pathogens in foods are a main driver of infant faecal-oral disease[6]. Field studies have demonstrated high bacterial contamination from *E. Coli* or Salmonella in weaning foods[7 8]. Of particular importance for the cleanliness of infant complementary foods are the quality of water, surfaces and utensils used to prepare and eat them[9-11] as well as the quality of the food ingredients procured.

Hence, the availability of WASH and food storage and preparation facilities is likely to determine exposure to infection. However, pathogen exposure can also arise when available facilities are used inadequately. For example, cultural norms and beliefs can restrict domestic food and hygiene practices – e.g., whether mothers are the principal decisionmakers about food preparation and hygiene in the household, or whether women are able to use the same toilet facilities as other household members[12].

However, while contamination of foods during cooking and feeding is common, the behavioural drivers have not been adequately explored. A recent systematic survey of 350 WASH evaluation projects in low and middle income countries (LMICs) found that around 15% of studies examined nutrition outcomes like stunting, and handwashing before food preparation, but only 5% reported on other food hygiene behaviours, such as whether food was stored appropriately and kitchen utensils were washed[13]. Whilst food safety and WASH are inter-linked, to date little attention has been paid to understanding the relationships and trade-offs between food safety, WASH and child stunting. The safety of livestock and fish derived foods is a particularly neglected area and there has been no systematic integration of these topics in food safety and WASH protocols[9].

The UKRI GCRF Action Against Stunting Hub (AASH) explores child stunting from the Whole Child Approach. The aim of this study is to examine WASH and food safety elements, their convergence and contributions to child stunting at the household level. It evaluates WASH and food safety practices across households in three study sites with the objective to provide evidence on potential exposure to pathogens through contaminated environment, drinking water, and complementary food, and associated risk factors.

METHOD AND ANALYSIS

Conceptual Framework

https://mc.manuscriptcentral.com/bmjpo

The manner in which WASH and food safety at the household level lead to changes in infection and dietary intake, thereby affecting child stunting, is illustrated in Figure 1, adapted from the UNICEF´s (1990) conceptual framework on the causes of malnutrition[14]. Inadequate WASH practices and food safety can contribute to childhood stunting directly, by exposing infants to infection via complementary foods contaminated during preparation and storage, or through feeding with unclean utensils, including the hands of carers. The safey of foods can also be compromised before arrival at households, throughout the different steps and processes of the food value chain. Those food hazards can be intrinsic to the food produced (e.g., pathogens from animal diseases, heavy metal marine contamination), or extrinsic through processing and handling (e.g., inadequate hygienic practices, aflatoxin contamination during storage)[15].

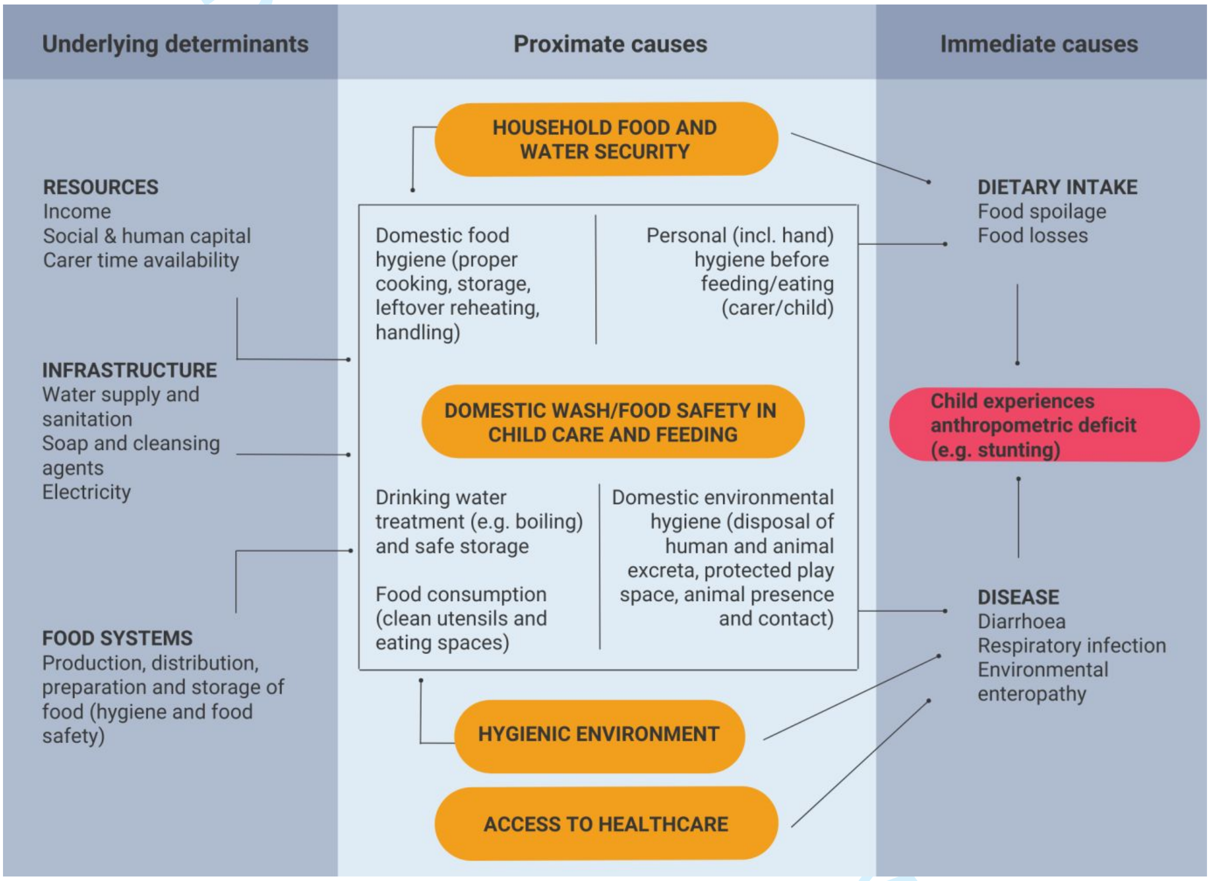

Figure 1: A conceptual framework showing the linkages of WASH and food safety with stunting at the household level

In theory, access to adequate WASH facilities enables appropriate drinking water, hygiene and food safety practices, contributing to reduction in transmission of food- and water-borne pathogens and improving nutrient uptake, and ultimately child growth and development[15].

Drinking water may be contaminated at the point of supply, or through transport and storage[10 11]. The availability of clean water is critical for optimal hygiene practices[5], to address contamination by human and animal faeces[16], and to prepare food hygienically. Sanitation facilities can enable safe faeces disposal, especially those of young children, which can be particularly pathogenic[17]. Faecal contamination can also come from the presence of domestic and wild animals or pests inside the household or in surrounding spaces [18]. It is expected that these sources of infection become more important as a child starts crawling and interacting with their environment. As such, understanding of

the determinants of food and hygiene practises in the household is key to improving infant health and nutrition[19].

Along with direct exposure, there is also an indirect route from water supply and sanitation facilities to childhood malnutrition, which operates through the opportunity costs of resource use. The time carers have to observe, correct, and address unhygienic behaviour, and the scarcity of time in relation to competing activities that need to be done by carers in the home or workplace, may also be detrimental for children's nutritional outcomes[20]. For example, it is typically women and older children, both of whom are more likely than others to be carers of young children, who spend time fetching water, engaging with animal fodder and collecting firewood. This may occur at the expense of adequate childcare and feeding, food preparation, education or rest[21]. When drinking water needs cannot be met affordably, households may need to purchase less food in order to afford enough water to drink. This kind of trade-off is clearly more prevalent for the poorest households, for whom malnutrition is also likely to be highest.

The WASH and food safety approaches, including their scope, evolution and methods are described in Table 1. Combining both WASH and food safety approaches provides an opportunity for more effective integration and learning of the two disciplines and their contributions to child growth and development. The two should converge when looking at household units with a central focus such as child stunting.

Table 1. Characteristics of Water, Sanitation and Hygiene (WASH) and Food Safety and Veterinary Public Health research

	**Water, Sanitation and Hygiene (WASH)**	**Food Safety and Veterinary Public Health**
Scope	To investigate deficiencies in water supply and quality, sanitation and hygiene to improve public health. Also, to evaluate the adequacy of hygiene standard to minimise food-borne diseases risks, and the role of water supply and sanitation access as enabling factors for improving child care, health and nutrition.	To pursue a comprehensive "farm to fork", "stable to table", "boat to throat" approach to prevent and minimise risks of food contamination at all stages of the food chain from production to human consumption and waste management.
Sector	Human-centred with a strong anchor in the public health sector.	Focus on animal source foods, zoonoses and their respective risks.

Confidential: For Review Only

Evolution	Historically, WASH research has focused on water-related disease transmission from human excreta (faecal-oral diseases). This has contributed to understanding the mechanisms of faecal-oral infection through water-borne, water-washed, and water-based routes. Early intervention studies focused on the provision of infrastructure, and information, education and communication (IEC).<br><br>Current research focuses more on behaviour change communication using bottom-up approaches, and incorporating the "A (Animals)" into WASH more holistically.	Veterinary public health traditionally looked at all food chain stages to identify where risks (e.g., biological, chemical, and physical) can emerge and how to prevent or mitigate them. The initial focus was on microbiological aspects with a strong technical dimension.<br><br>Veterinary public health now looks at human behaviour more widely, bringing in social science aspects and economics to understand better practices and the motivations behind them. There is a (slow) move towards systems thinking, and a lot of progress in quantitative microbiological and epidemiological studies, detection techniques, etc.
Research methods	▪ Interventions (e.g., randomised controlled trials)<br>▪ Cross-sectional, questionnaire- based surveys<br>▪ Longitudinal questionnaire-based surveys<br>▪ Spot checks<br>▪ Direct observation<br>▪ Biological sampling and testing<br>▪ Risk assessments<br>▪ Qualitative research on behaviour, practices and perceptions, including participatory approaches	

Confidential: For Review Only

Study Design

This is a longitudinal observational study where women are recruited during pregnancy and mother-infant pairs followed up to 24 months after birth. This protocol involves multiple approaches to assess WASH and food safety practices in households of women enrolled in the study, in Hyderabad, India, Lombok, Indonesia and Kaffrine, Senegal, at predefined timepoints (see AASH observational cohort description in Heffernan et al in this supplement).

Data collection

This WASH and food safety data collection protocol focuses on three areas: exposure pathways to wider environmental pathogens, foodborne hazards, and waterborne hazards. Four data collection tools for the household-level have been developed, namely: i) questionnaire-based interviews; ii) spot-checks; iii) biological sampling; and iv) direct observation. The interviews and spot-checks take place simultaneously during home visits at 9, 12 and 18 months after birth. Direct observation and biological sampling are conducted during a separate subsequent visit at each timepoing. The food safety component of the data collection focuses on nutrient-rich foods with the potential to alleviate stunting, particularly on animal source foods (ASFs). This is due to their important nutritional profile[22], their documented potential to alleviate stunting[23-26], and their high food safety risk profile. Selected ASFs will be considered at each country, according to their relevance.

Trained enumerators collect data using electronic tablets in all study households, as summarised in Table 2. Data collection tools developed in English are translated into local languages and backtranslated into English to check for accuracy, and piloted in the field.

Table 2. Summary of the integrated study methods and tools used to assess WASH and food safety at the household level

Tool	Description	Sample size	Timepoints
Questionnaire-based interview	Mothers are asked about infant and young child feeding (IYCF) practices, household food expenditure, and the main household decision-makers and influencers of these activities. Physical activity questionnaires ask mothers about the times spent on work and housekeeping including collecting water, fodder and fuel. Furthermore, there are questions on water sources, water security, sanitation and handwashing. Other questions focus on the practices of acquisition, transport, storage, preparation of ASF, and the points of potential contamination in the household. Finally, other questions refer to the presence and behaviour of animals and potential transmission pathways associated with close proximity to animals, such as direct contact between animals and children or food.<br><br>The hazards and risks prior to arrival of ASFs in the household are elucidated in a separate component, at the value chain level (see Cooper et al in this supplement).	Whole cohort (500 households per country)	At 9, 12, and 18 months after birth
Spot-check	Enumerators observe behavioural issues alongside the interview questionnaire. The data are recorded using a check list on the availability and type of sanitation facilities available at the households (e.g., the place for defecation and proximity), handwashing infrastructure (e.g., location, availability of water and soap), presence of animals (e.g., livestock, pests), food storage facilities and practices (e.g., functioning refrigerator), and food preparation (e.g., easy to clean equipment).	Whole cohort (500 households per country)	At 9, 12, and 18 months after birth

Confidential: For Review Only

Direct observation	Enumerators stay at the household for 3 hours around a feeding event, observing the mothers perform their daily activities and taking a passive approach to blend into the background in order to better observe hygiene related behaviours. Structured observation include activities related to water collection and storage, as well as personal, environmental and food hygiene (food preparation, storage, child feeding, handwashing, and animal contact). This data collection method is a gold standard for actual observation of behavioural practices.	Subsample (approximately one-third of full cohort in each country)	At 9, 12, and 18 months after birth
Biological sampling and testing	Enumerators collect samples of stored water, foods to be consumed by the children (as close to the feeding of the child as possible), and a swab of the main food preparation area at the households. Samples of hands rinse are optional.<br><br>The samples are stored in a coolbox and transported to a specialised laboratory for analysis. The samples are processed in-country and tested for overall contamination and for selected pathogens (such as Salmonella, Shigella, E.coli, and Campylobacter for food and E.coli/faecal indicators for surfaces and water), using standard laboratory protocols (of conventional culture and PCR). Part of the samples collected are used for lab analysis and the remaining part stored under -20°C for future analysis and crosschecking.	Subsample (approximately one-third of full cohort in each country)	At 9, 12, and 18 months after birth

ASF: animal source foods; IYCF: Infant and young child feeding; PCR: Polymerase chain reaction

Confidential: For Review Only

Data management and analysis

Data will be collected and analysed at each of the countries using tailored Standard Operating Procedures (SOPs). Data collected at the households and laboratories will be checked for accuracy and then transmitted to a central repository for analysis.

Descriptive, comparative and regression analyses will be used to investigate the impact of WASH and food safety on child stunting. Factors relating to WASH and food safety amenities and practices will be considered simultaneously as external risks or preventive factors influencing the incidence and severity of stunting, using multivariate statistical approaches. Data triangulation will also be used, firstly, to address biases in individual data collection tools. For example, known biases occur when measures are reported by participants, particularly by those people for whom illness becomes normalised after repeated bouts of infection[27]. Observation of behaviours, through enumerator

presence in the household at certain times of day, can help to mitigate reporting bias. However, surveys and observation may also affect participant motivation, particularly when repeated over long periods. Extensive collection of samples for laboratory testing can be done, but are expensive and have their own weaknesses [28]. Secondly, data triangulation through statistical modelling will be used to the determine the relationship between WASH and food safety in each of the study contexts, and to explore relevant questions such as whether they are complements or substitutes in child health and nutrition. As such, a mix of approaches is being used in this study to address these potential challenges.

Patient and Public Involvement statement

Study participants have not been involved in the general design of the study. However, the study was designed in partnership with local research partners and stakeholders with experience in working at each of the settings. Local enumerators helped finalise the data collection tools during training and pilot testing.

Extensive consultations will be organised with the local communities to understand the ethos of the research including the involvement of participants and the implications of the research. Question and answer sections will be held during the consulations to answer any questions and/or concerns raised. Study participants and the public will be involved in the dissemination of the study's findings through community discussion or engagement events.

DISCUSSION

Poor food safety and inadequate WASH, including getting in direct contact with animals, their produce and/or their faeces, are thought to be important contributors to undernutrition. The potential mechanisms include nutrient loss (e.g., during diarrhoeal episodes), energy used by the immune system (e.g., in combating illnesses), and increased intestinal permeability and reduction in nutrients absorption (e.g., enteropathy) [29]. Other indirect pathways include scarcity of income and time with which undertake childcare and feeding practices. Cumulatively over the first two years of life, these factors can contribute to stunting expressed as linear growth faltering. However, the causes of infant stunting are not exclusive to these factors, and therefore it is critical to cohesively consider the multiple determinants involved. The UKRI GCRF AASH is using an interdisciplinary approach to investigate this variety of factors causing stunting with the aim to identify solutions for the ameriolation of growth and development in early childhood at the 'whole-child' level. Within such an interdisciplinary framework, this study design will generate comprehensive evidence on the contribution of WASH, and food safety at the household level.

Challenges to conducting integrated assessment in WASH and food safety in a multi-country study like this are various, including identifying key ASFs and key hazards in a systematic way, or the analysis of pathways linking hygiene, food and water safety, and sanitation data into the typologies of stunting. For this purpose, we developed holistic data collection tools relevant to each study setting, to measure risk factors for exposure to food- and water-borne disease. Multiple household-level interventions are available in both WASH and food safety but, to date, their effectiveness has not been assessed together, despite the similarity in the exposure mechanisms they are targeting. The effectiveness of relavant interventions and technologies in addressing malnutrition depends on both their biological efficacy, and the degree to which consumer behaviour adapts to accept and use the new approach, in the environment where it is based[30]. We draw on an approach and suggest data collection tools through which these various underlying and proximate determinants of child growth may be analysed.

Confidential: For Review Only

Additionally, the value chains for ASF generally present important potential to improve availability, accessibility, affordability and safety for the most nutritionally vulnerable population groups[31]. This study will also provide information that will help to identify entry points in chains where upgrading may be feasible and welcome by stakeholders (e.g., consumers, government, and businesses). Based on secondary dietary and nutritional data, a systematic literature review of food safety hazards in ASF value chains in each study setting and local experts' feedback, commodities of likely relevance are eggs in all countries, milk in India and Senegal, and fish in Indonesia (see AASH food system protocol description in Cooper et al in this supplement for value chain work).

The data generated from the study will help pave the way for future interventions to to manage factors associated with and prevent stunting.

ETHICS AND DISSEMINATION

Ethical approval for the study was granted by the Institutional ethics committees of the London School of Hygiene and Tropical Medicine (17915/RR/17513), the Social Science Research Ethical Review Board at the Royal Veterinary College (URN SR2020-0198) and the International Livestock Research Institute Institutional Research Ethics Committee (ILRI-IREC2020-33). In-country approvals were also granted: the National Institute of Nutrition (ICMR), Ministry of Health and Family Welfare, Government of India (CR/04/I/2021), the Health Research Ethics Committee, University of Indonesia and Dr Cipto Mangunkusumo Hospital (KET-887/UN2.F1/ETIK/PPM.00.02/2019), and the Comité National d'Ethique pour la Recherche en Santé, Senegal (Protocole SEN19/78).

Findings of the study will be disseminated through publication in peer-reviewed journals, presented at relevant international conferences, public engagement events, and policy-maker and stakeholder events. Data generated from the study will be deposited on publicly available data repository.

Confidential: For Review Only

REFERENCES

1. Checkley W, Buckley G, Gilman RH, et al. Multi-country analysis of the effects of diarrhoea on childhood stunting. *International journal of epidemiology* 2008;37(4):816-30.

2. Black RE, Cousens S, Johnson HL, et al. Global, regional, and national causes of child mortality in 2008: a systematic analysis. *The lancet* 2010;375(9730):1969-87.

3. Liu L, Johnson HL, Cousens S, et al. Global, regional, and national causes of child mortality: an updated systematic analysis for 2010 with time trends since 2000. *The lancet* 2012;379(9832):2151-61.

4. Havelaar AH, Kirk MD, Torgerson PR, et al. World Health Organization Global Estimates and Regional Comparisons of the Burden of Foodborne Disease in 2010. *PLOS Medicine* 2015;12(12):e1001923. doi: 10.1371/journal.pmed.1001923

5. White GF, Bradley DJ, White AU. Drawers of water: domestic water use in East Africa. *Bulletin of the World Health Organization* 1972;80(1):63-73.

6. Motarjemi Y, Käferstein F, Moy G, et al. Contaminated weaning food: a major risk factor for diarrhoea and associated malnutrition. *Bulletin of the World health Organization* 1993;71(1):79.

7. Touré O, Coulibaly S, Arby A, et al. Piloting an intervention to improve microbiological food safety in Peri-Urban Mali. *International journal of hygiene and environmental health* 2013;216(2):138-45. doi: https://doi.org/10.1016/j.ijheh.2012.02.003

8. Kinkese DM, Hang'ombe MB, Toure O, et al. Contamination of complementary weaning foods for children with Escherichia coli and salmonella species in Lusaka District, Zambia. *Journal of Preventive and Rehabilitative Medicine* (1):19-31. doi: Doi:10.21617/jprm.2018.0101.3

9. Gautam OP, Schmidt W-P, Cairncross S, et al. Trial of a novel intervention to improve multiple food hygiene behaviors in Nepal. *The American journal of tropical medicine and hygiene* 2017;96(6):1415.

10. Wright J, Gundry S, Conroy R. Household drinking water in developing countries: a systematic review of microbiological contamination between source and point-of-use. *Tropical medicine & international health* 2004;9(1):106-17.

11. Lule JR, Mermin J, Ekwaru JP, et al. Effect of home based water chlorination and safe storage on diarrhea among persons with human immonodeficiency virus in Uganda. *American Journal of Tropical Medicine & Hygiene* 2005;73:926–33.

12. Curtis V. Hygiene: how myths, monsters, and mothers-in-law can promote behaviour change. *Journal of Infection* 2001;43(1):75-79.

13. Chirgwin H, Cairncross S, Zehra D, et al. Interventions promoting uptake of water, sanitation and hygiene (WASH) technologies in low-and middle-income countries: An evidence and gap map of effectiveness studies. *Campbell Systematic Reviews* 2021;17(4):e1194.

14. Unicef. Strategy for improved nutrition of children and women in developing countries. A UNICEF policy review. New York: UNICEF 1990.

15. Unnevehr L, Grace D. Aflatoxins: Finding solutions for improved food safety. Washington, DC: IFPRI 2013.

16. Penakalapati G, Swarthout J, Delahoy MJ, et al. Exposure to animal feces and human health: a systematic review and proposed research priorities. *Environmental science & technology* 2017;51(20):11537-52.

Confidential: For Review Only

17. Majorin F, Torondel B, Chan GKS, et al. Interventions to improve disposal of child faeces for preventing diarrhoea and soil-transmitted helminth infection. *Cochrane Database of Systematic Reviews* 2019(9)

18. Prendergast AJ, Gharpure R, Mor S, et al. Putting the "A" into WaSH: a call for integrated management of water, animals, sanitation, and hygiene. *The Lancet Planetary Health* 2019;3(8):e336-e37. doi: https://doi.org/10.1016/S2542-5196(19)30129-9

19. Aunger R, Curtis V. Behaviour Centred Design: towards an applied science of behaviour change. *Health psychology review* 2016;10(4):425-46. doi: http://dx.doi.org/10.1080/17437199.2016.1219673

20. Popkin BM, Solon FS. Income, time, the working mother and child nutriture. *Journal of tropical pediatrics* 1976;22(4):156-66.

21. Cairncross S, Cuff J. Water use and health in Mueda, Mozambique. *Transactions of the Royal Society of Tropical Medicine and Hygiene* 1987;81(1):51-54.

22. Iannotti L, Tarawali SA, Baltenweck I, et al. Livestock-derived foods and sustainable healthy diets. 2021

23. Iannotti LL, Lutter CK, Stewart CP, et al. Eggs in early complementary feeding and child growth: a randomized controlled trial. *Pediatrics* 2017;140(1)

24. Headey D, Hirvonen K, Hoddinott J. Animal sourced foods and child stunting: Wiley Online Library, 2018.

25. De Beer H. Dairy products and physical stature: a systematic review and meta-analysis of controlled trials. *Economics & Human Biology* 2012;10(3):299-309.

26. Neumann CG, Murphy SP, Gewa C, et al. Meat supplementation improves growth, cognitive, and behavioral outcomes in Kenyan children. *the Journal of Nutrition* 2007;137(4):1119-23.

27. Briscoe J, Feachem RG, Rahaman MM. Evaluating health impact: water supply, sanitation, and hygiene education: IDRC, Ottawa, ON, CA 1986.

28. Goddard FG, Ban R, Barr DB, et al. Measuring environmental exposure to enteric pathogens in low-income settings: review and recommendations of an interdisciplinary working group. *Environmental science & technology* 2020;54(19):11673-91. doi: DOI: 10.1021/acs.est.0c02421

29. Bourke CD, Berkley JA, Prendergast AJ. Immune dysfunction as a cause and consequence of malnutrition. *Trends in immunology* 2016;37(6):386-98.

30. Cumming O, Arnold BF, Ban R, et al. The implications of three major new trials for the effect of water, sanitation and hygiene on childhood diarrhea and stunting: a consensus statement. *BMC medicine* 2019;17(1):1-9. doi: 10.1186/s12916-019-1410-x

31. Alarcon P, Dominguez-Salas P, Fèvre EM, et al. The importance of a food systems approach to low and middle income countries and emerging economies: a review of theories and its relevance for disease control and malnutrition. *Frontiers in Sustainable Food Systems* 2021:92. doi: https://doi.org/10.3389/fsufs.2021.642635

Confidential: For Review Only

AUTHORS CONTRIBUTIONS

All authors were involved in the study design and the preparation of the study tools and protocols at different stages; BH, HW and PDS drafted the manuscript sections; all other authors critically reviewed the manuscript. PDS and HW share first authorship.

We want to thank Alessia Gasco for producing the conceptual framework and Claire Heffernan, Kaitlin Conway and Modou Jobarteh for careful editing of the manuscript.

FUNDING STATEMENT

This work was supported by the UKRI-GCRF Action Against Stunting Hub (Project ref. MR/S01313X/1). The views expressed in this manuscript are those of the authors and do not necessarily represent the views of the funders.

COMPETING INTEREST STATEMENT

The authors declare no conflict of interest.

Confidential: For Review Only

BMJ Paediatrics Open

Understanding the role of household hygiene practices and foodborne disease risks in child stunting: a UKRI GCRF Action Against Stunting Hub protocol paper

Journal:	*BMJ Paediatrics Open*
Manuscript ID	bmjpo-2022-001695.R1
Article Type:	Protocol
Date Submitted by the Author:	06-Nov-2022
Complete List of Authors:	Dominguez-Salas, Paula; University of Greenwich; International Livestock Research Institute (ILRI)<br>Waddington, Hugh Sharma; London School of Hygiene and Tropical Medicine, Department of Disease Control<br>Grace, Delia; University of Greenwich; International Livestock Research Institute (ILRI)<br>Bosire, Caroline; International Livestock Research Institute (ILRI)<br>Moodley, Arshnee; International Livestock Research Institute (ILRI)<br>Kulkarni, Bharati; National Institute of Nutrition<br>Dasi, Teena; National Institute of Nutrition<br>B, Santosh Kumar; National Institute of Nutrition, Clinical Division<br>Kumar, Ramachandrappa Naveen; National Institute of Nutrition<br>Fahmida, Umi; Southeast Asian Ministry of Education Organisation Regional Centre for Food and Nutrition (SEAMEO RECFON)<br>Htet, Min Kyaw; Southeast Asian Ministry of Education Organisation Regional Centre for Food and Nutrition (SEAMEO RECFON)<br>Sudibya, Arienta R.P.; Southeast Asian Ministry of Education Organisation Regional Centre for Food and Nutrition (SEAMEO RECFON)<br>Faye, Babacar; Université Cheikh Anta Diop (UCAD)<br>Tine, Roger ; University of Cheikh Anta DIOP, Department of Parasitology-Mycology<br>Saxena, Deepak ; Public Health Foundation of India<br>Dreibelbis, Robert; London School of Hygiene and Tropical Medicine, Department of Disease Control<br>Häsler, Barbara N; Royal Veterinary College (RVC), Department of Pathobiology and Population Sciences
Keywords:	Epidemiology, Microbiology, Data Collection

SCHOLARONE™
Manuscripts

I, the Submitting Author has the right to grant and does grant on behalf of all authors of the Work (as defined in the below author licence), an exclusive licence and/or a non-exclusive licence for contributions from authors who are: i) UK Crown employees; ii) where BMJ has agreed a CC-BY licence shall apply, and/or iii) in accordance with the terms applicable for US Federal Government officers or employees acting as part of their official duties; on a worldwide, perpetual, irrevocable, royalty-free basis to BMJ Publishing Group Ltd ("BMJ") its licensees and where the relevant Journal is co-owned by BMJ to the co-owners of the Journal, to publish the Work in this journal and any other BMJ products and to exploit all rights, as set out in our licence.

The Submitting Author accepts and understands that any supply made under these terms is made by BMJ to the Submitting Author unless you are acting as an employee on behalf of your employer or a postgraduate student of an affiliated institution which is paying any applicable article publishing charge ("APC") for Open Access articles. Where the Submitting Author wishes to make the Work available on an Open Access basis (and intends to pay the relevant APC), the terms of reuse of such Open Access shall be governed by a Creative Commons licence – details of these licences and which Creative Commons licence will apply to this Work are set out in our licence referred to above.

Other than as permitted in any relevant BMJ Author's Self Archiving Policies, I confirm this Work has not been accepted for publication elsewhere, is not being considered for publication elsewhere and does not duplicate material already published. I confirm all authors consent to publication of this Work and authorise the granting of this licence.

Confidential: For Review Only

Understanding the role of household hygiene practices and foodborne disease risks in child stunting: a UKRI GCRF Action Against Stunting Hub protocol paper

Paula Dominguez-Salas[ab], Hugh Sharma Waddington[c], Delia Grace[ab], Caroline Bosire[b], Arshnee Moodley[b], Bharati Kulkarni[d], Teena Raajev[d], Santosh Kumar Banjara[d], Ramachandrappa Naveen Kumar [d], Umi Fahmida[e], Kyaw Htet[e], Arienta R.P. Sudibya[e], Babacar Faye[f], Roger C. Tine[f], Deepak Saxena[g], Robert Dreibelbis[c], Barbara Häsler[h]

[a]Natural Resources Institute (NRI), University of Greenwich, London, United Kingdom

[b]International Livestock Research Institute (ILRI), Nairobi, Kenya

[c]Environmental Health Group, Department of Disease Control, London School of Hygiene and Tropical Medicine, London, United Kingdom

[d]ICMR- National Institute of Nutrition (ICMR), Hyderabad, Telangana, India

[e]Southeast Asian Ministers of Education Organization Regional Center for Food and Nutrition (SEAMEO-RECFON)-Pusat Kajian Gizi Regional, Universitas Indonesia, Jakarta, Indonesia

[f]Department of Parasitology-Mycology, University of Cheikh Anta DIOP, Dakar, Senegal

[g]Indian Institute of Public Health Gandhinagar (IIPHG), India

[h]Department of Pathobiology and Population Sciences, Royal Veterinary College (RVC), Hatfield, United Kingdom

Corresponding author:

Paula Dominguez Salas, Natural Resources Institute, University of Greenwich, Chatham Maritime, Kent, United Kingdom, P.DominguezSalas@gre.ac.uk

Keywords: food safety, hygiene, water, sanitation, child stunting, protocol

Confidential: For Review Only

ABSTRACT

Introduction: Environmental hygiene and food safety are important determinants of child stunting. This research aims to explore the relationship between child stunting and household hygiene practices and behaviours, including the availability of water, sanitation and hygiene (WASH) facilities; the use of safe food and good quality drinking water (especially when used for complementary feeding); hygienic practices in food transport, storage and preparation; and the control of cross-contamination from animals, their produce and waste.

Methods and analysis: This study is part of a wider observational study which aims to investigate the interdisciplinary factors contributing to child stunting using a 'whole child' paradigm. The observational study recruits women during pregnancy in Hyderabad, India, Lombok, Indonesia and Kaffrine, Senegal, and dyads (i.e., 500 mother-infant pairs per country) are followed longitudinally up to 24 months after birth. Within the interdisciplinary niche, the study herein has developed tools to investigate the potential exposure pathways to environmental pathogen contamination of foods and water. Holistic WASH and food safety data collection tools have been developed to explore exposure pathways at the household-level, including: i) survey questionnaires; ii) spot-checks; iii) biological sampling of drinking water, food and domestic surfaces; and iv) direct observation. An integrated analytical approach will be used to triangulate the evidence in order to examine the relationships between child stunting, WASH and food safety behaviours.

Ethics and dissemination: Ethical approval of the study was granted by the ethics committee of the LSHTM, RVC, ILRI, ICMR, IIPHG, SEAMEO-RECFON, University of Cheikh Anta Diop. Findings of the study will be disseminated through publication in peer-reviewed journals, relevant international conferences, public engagement events, and policy-maker and stakeholder events.

Confidential: For Review Only

SUMMARY BOX

- **What is known about the subject**
  - From the moment complementary feeding begins, the chances of exposure to food contamination and infectious disease increase exponentially
  - Adequate WASH and food safety practices can play a key role in reducing infectious disease transmission and child malnutrition.
  - The contribution of hygiene behaviours and food contamination in driving transmission of infections and childhood stunting requires further investigation.

- **What this study hopes to add**
  - Development, application and analysis of integrated WASH and food safety tools, towards a holistic understanding of how these practices and behaviours impact child stunting
  - Evidence towards a new typology of child stunting, with a focus on exposure pathways for pathogens, and to support programmes and policies to minimise stunting in childhood.

Confidential: For Review Only

INTRODUCTION

The incidence and severity of stunting in childhood is closely linked to exposure to infection from food, drinking water and the wider environment[1]. Infections associated with contaminated water or insufficient water, sanitation and hygiene (WASH) are responsible for an estimated 21% of the total global burden of diseases[2], contributing to the outbreak and chronicity of preventable infections such as diarrhoeal diseases and ARI (Acute Respiratory Infections), which are the two leading causes of death in children globally[3]. In addition, the World Health Organization (WHO) Foodborne Disease Burden Epidemiology Reference Group (FERG), estimated that approximately 40% of the global burden of foodborne diseases occurs in children under the age of five years[4]. A common cause of foodborne infections is the consumption of raw or undercooked meat, fish, seafood, eggs, fresh produce and dairy products contaminated by norovirus, Campylobacter, non-typhoidal Salmonella or pathogenic *E. coli*[4]. Food hygiene is a primary concern in the preparation of complementary foods - for example, bottles often cannot be adequately sterilised and perishable complementary foods are often left unrefrigerated or eaten using unclean utensils or with unwashed hands.

Disease transmission from faeces can be food- or water-borne (i.e., oral ingestion of contaminated food and water), water-washed or water-scarce (i.e., spread through inadequate hand and food hygiene) and water-based (i.e., transmitted by parasites that penetrate skin in water, such as schistosomiasis, or by walking barefoot on contaminated soil in the case of hookworm)[5]. For example, theory and evidence suggests that pathogens in foods are a main driver of infant faecal-oral disease[6]. Field studies have demonstrated high bacterial contamination from *E. Coli* or Salmonella in weaning foods[7 8]. Of particular importance for the cleanliness of infant complementary foods are the quality of water, surfaces and utensils used to prepare and eat them[9-11] as well as the quality of the food ingredients procured.

Hence, the availability of WASH and food storage and preparation facilities is likely to determine exposure to infection. However, pathogen exposure can also arise when available facilities are used inadequately. For example, cultural norms and beliefs can restrict domestic food and hygiene practices – e.g., whether mothers are the principal decisionmakers about food preparation and hygiene in the household, or whether women are able to use the same toilet facilities as other household members[12].

However, while contamination of foods during cooking and feeding is common, the behavioural drivers have not been adequately explored. A recent systematic survey of 350 WASH evaluation projects in low and middle income countries (LMICs) found that around 15% of studies examined nutrition outcomes like stunting, and handwashing before food preparation, but only 5% reported on other food hygiene behaviours, such as whether food was stored appropriately and kitchen utensils were washed[13]. Whilst food safety and WASH are inter-linked, to date little attention has been paid to understanding the relationships and trade-offs between food safety, WASH and child stunting. The safety of livestock and fish derived foods is a particularly neglected area and there has been no systematic integration of these topics in food safety and WASH protocols[9].

The UKRI GCRF Action Against Stunting Hub (AASH) explores child stunting from the Whole Child Approach. The aim of this study is to examine WASH and food safety elements, their convergence and contributions to child stunting at the household level. It evaluates WASH and food safety practices across households in three study sites with the objective to provide evidence on potential exposure to pathogens through contaminated environment, drinking water, and complementary food, and associated risk factors.

METHOD AND ANALYSIS

Conceptual Framework

Confidential: For Review Only

The manner in which WASH and food safety at the household level lead to changes in infection and dietary intake, thereby affecting child stunting, is illustrated in Figure 1, adapted from the UNICEF´s (1990) conceptual framework on the causes of malnutrition[14]. Inadequate WASH practices and food safety can contribute to childhood stunting directly, by exposing infants to infection via complementary foods contaminated during preparation and storage, or through feeding with unclean utensils, including the hands of carers. The safey of foods can also be compromised before arrival at households, throughout the different steps and processes of the food value chain. Those food hazards can be intrinsic to the food produced (e.g., pathogens from animal diseases, heavy metal marine contamination), or extrinsic through processing and handling (e.g., inadequate hygienic practices, aflatoxin contamination during storage)[15].

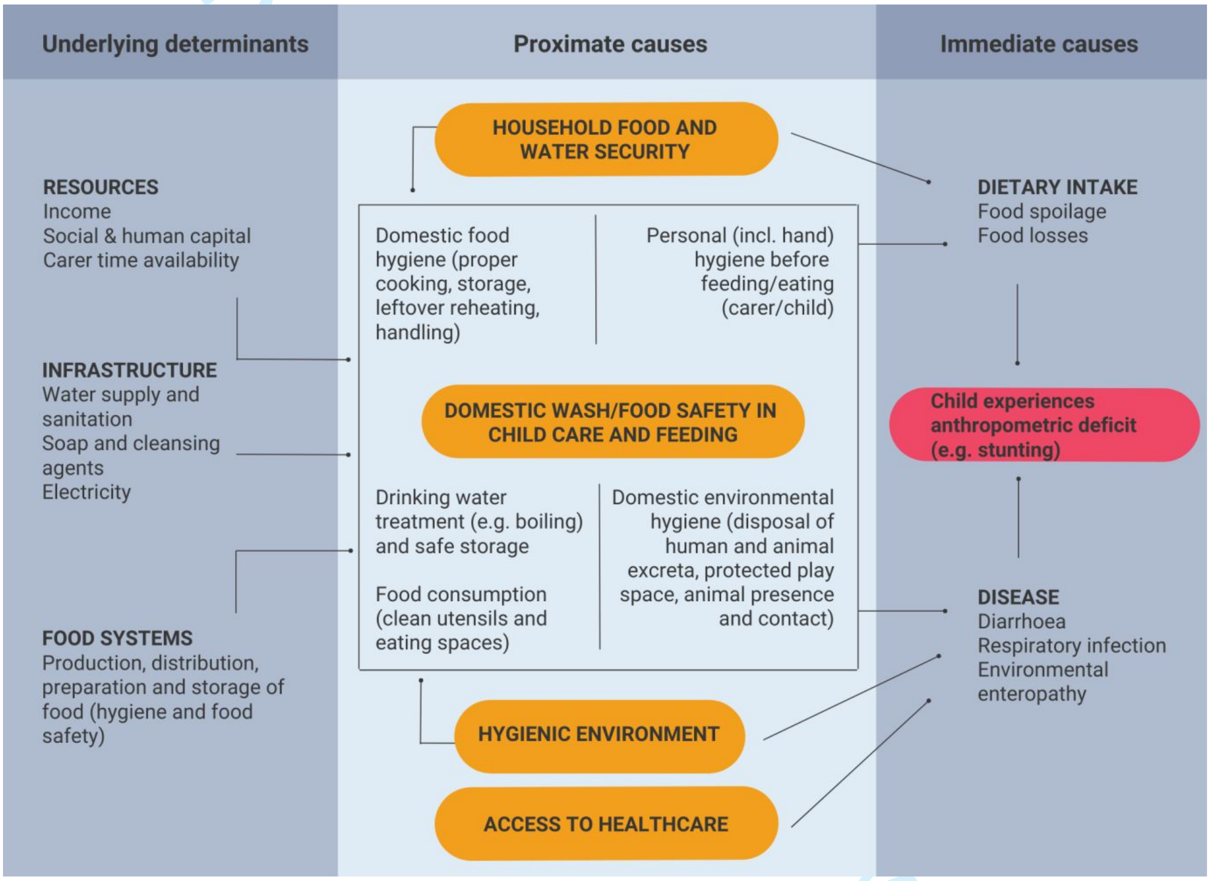

Figure 1: A conceptual framework showing the linkages of WASH and food safety with stunting at the household level

In theory, access to adequate WASH facilities enables appropriate drinking water, hygiene and food safety practices, contributing to reduction in transmission of food- and water-borne pathogens and improving nutrient uptake, and ultimately child growth and development[15].

Drinking water may be contaminated at the point of supply, or through transport and storage[10 11]. The availability of clean water is critical for optimal hygiene practices[5], to address contamination by human and animal faeces[16], and to prepare food hygienically. Sanitation facilities can enable safe faeces disposal, especially those of young children, which can be particularly pathogenic[17]. Faecal contamination can also come from the presence of domestic and wild animals or pests inside the household or in surrounding spaces [18]. It is expected that these sources of infection become more important as a child starts crawling and interacting with their environment. As such, understanding of

the determinants of food and hygiene practises in the household is key to improving infant health and nutrition[19].

Along with direct exposure, there is also an indirect route from water supply and sanitation facilities to childhood malnutrition, which operates through the opportunity costs of resource use. The time carers have to observe, correct, and address unhygienic behaviour, and the scarcity of time in relation to competing activities that need to be done by carers in the home or workplace, may also be detrimental for children's nutritional outcomes[20]. For example, it is typically women and older children, both of whom are more likely than others to be carers of young children, who spend time fetching water, engaging with animal fodder and collecting firewood. This may occur at the expense of adequate childcare and feeding, food preparation, education or rest[21]. When drinking water needs cannot be met affordably, households may need to purchase less food in order to afford enough water to drink. This kind of trade-off is clearly more prevalent for the poorest households, for whom malnutrition is also likely to be highest.

The WASH and food safety approaches, including their scope, evolution and methods are described in Table 1. Combining both WASH and food safety approaches provides an opportunity for more effective integration and learning of the two disciplines and their contributions to child growth and development. The two should converge when looking at household units with a central focus such as child stunting.

Table 1. Characteristics of Water, Sanitation and Hygiene (WASH) and Food Safety and Veterinary Public Health research

	**Water, Sanitation and Hygiene (WASH)**	**Food Safety and Veterinary Public Health**
Scope	To investigate deficiencies in water supply and quality, sanitation and hygiene to improve public health. Also, to evaluate the adequacy of hygiene standard to minimise food-borne diseases risks, and the role of water supply and sanitation access as enabling factors for improving child care, health and nutrition.	To pursue a comprehensive "farm to fork", "stable to table", "boat to throat" approach to prevent and minimise risks of food contamination at all stages of the food chain from production to human consumption and waste management.
Sector	Human-centred with a strong anchor in the public health sector.	Focus on animal source foods, zoonoses and their respective risks.

Confidential: For Review Only

Evolution	Historically, WASH research has focused on water-related disease transmission from human excreta (faecal-oral diseases). This has contributed to understanding the mechanisms of faecal-oral infection through water-borne, water-washed, and water-based routes. Early intervention studies focused on the provision of infrastructure, and information, education and communication (IEC).<br><br>Current research focuses more on behaviour change communication using bottom-up approaches, and incorporating the "A (Animals)" into WASH more holistically.	Veterinary public health traditionally looked at all food chain stages to identify where risks (e.g., biological, chemical, and physical) can emerge and how to prevent or mitigate them. The initial focus was on microbiological aspects with a strong technical dimension.<br><br>Veterinary public health now looks at human behaviour more widely, bringing in social science aspects and economics to understand better practices and the motivations behind them. There is a (slow) move towards systems thinking, and a lot of progress in quantitative microbiological and epidemiological studies, detection techniques, etc.
Research methods	▪ Interventions (e.g., randomised controlled trials)<br>▪ Cross-sectional, questionnaire- based surveys<br>▪ Longitudinal questionnaire-based surveys<br>▪ Spot checks<br>▪ Direct observation<br>▪ Biological sampling and testing<br>▪ Risk assessments<br>▪ Qualitative research on behaviour, practices and perceptions, including participatory approaches	

Confidential: For Review Only

Study Design

This is a longitudinal observational study where women are recruited during pregnancy and mother-infant pairs followed up to 24 months after birth. This protocol involves multiple approaches to assess WASH and food safety practices in households of women enrolled in the study, in Hyderabad, India, Lombok, Indonesia and Kaffrine, Senegal, at predefined timepoints (see AASH observational cohort description in Heffernan et al in this supplement).

Data collection

This WASH and food safety data collection protocol focuses on three areas: exposure pathways to wider environmental pathogens, foodborne hazards, and waterborne hazards. Four data collection tools for the household-level have been developed, namely: i) a module for WASH and food safety to be included to the cohort questionnaire-based interviews (see Appendix 1); ii) spot-checks (see Appendix 2); iii) biological sampling; and iv) direct observation (see Appendix 3). The interviews and spot-checks take place simultaneously during home visits at 9, 12 and 18 months after birth. Direct observation and biological sampling are conducted during a separate subsequent visit at each timepoing. The food safety component of the data collection focuses on nutrient-rich foods with the potential to alleviate stunting, particularly on animal source foods (ASFs). This is due to their important

nutritional profile[22], their documented potential to alleviate stunting[23-26], and their high food safety risk profile. Selected ASFs will be considered at each country, according to their relevance.

Trained enumerators collect data using electronic tablets in all study households, as summarised in Table 2. Data collection tools developed in English are translated into local languages and backtranslated into English to check for accuracy, and piloted in the field.

Table 2. Summary of the integrated study methods and tools used to assess WASH and food safety at the household level

Tool	Description	Sample size	Timepoints
Questionnaire-based interview	Mothers are asked about infant and young child feeding (IYCF) practices, household food expenditure, and the main household decision-makers and influencers of these activities. Physical activity questionnaires ask mothers about the times spent on work and housekeeping including collecting water, fodder and fuel. Furthermore, there are questions on water sources, water security, sanitation and handwashing. Other questions focus on the practices of acquisition, transport, storage, preparation of ASF, and the points of potential contamination in the household. Finally, other questions refer to the presence and behaviour of animals and potential transmission pathways associated with close proximity to animals, such as direct contact between animals and children or food.<br><br>The hazards and risks prior to arrival of ASFs in the household are elucidated in a separate component, at the value chain level (see Cooper et al in this supplement).	Whole cohort (500 households per country)	At 9, 12, and 18 months after birth
Spot-check	Enumerators observe behavioural issues alongside the interview questionnaire. The data are recorded using a check list on the availability and type of sanitation facilities available at the households (e.g., the place for defecation and proximity), handwashing infrastructure (e.g., location, availability of water and soap), presence of animals (e.g., livestock, pests), food storage facilities and practices (e.g., functioning refrigerator), and food	Whole cohort (500 households per country)	At 9, 12, and 18 months after birth

Confidential: For Review Only

	preparation (e.g., easy to clean equipment).		
Direct observation	Enumerators stay at the household for 3 hours around a feeding event, observing the mothers perform their daily activities and taking a passive approach to blend into the background in order to better observe hygiene related behaviours. Structured observation include activities related to water collection and storage, as well as personal, environmental and food hygiene (food preparation, storage, child feeding, handwashing, and animal contact). This data collection method is a gold standard for actual observation of behavioural practices.	Subsample (approximately one-third of full cohort in each country)	At 9, 12, and 18 months after birth
Biological sampling and testing	Enumerators collect samples of stored water, foods to be consumed by the children (as close to the feeding of the child as possible), and a swab of the main food preparation area at the households. Samples of hands rinse are optional.<br><br>The samples are stored in a coolbox and transported to a specialised laboratory for analysis. The samples are processed in-country and tested for overall contamination and for selected pathogens (such as Salmonella, Shigella, E.coli, and Campylobacter for food and E.coli/faecal indicators for surfaces and water), using standard laboratory protocols (of conventional culture and PCR). Part of the samples collected are used for lab analysis and the remaining part stored under -20°C for future analysis and crosschecking.	Subsample (approximately one-third of full cohort in each country)	At 9, 12, and 18 months after birth

ASF: animal source foods; IYCF: Infant and young child feeding; PCR: Polymerase chain reaction

Confidential: For Review Only

Data management and analysis

Data will be collected and analysed at each of the countries using tailored Standard Operating Procedures (SOPs). Data collected at the households and laboratories will be checked for accuracy and then transmitted to a central repository for analysis.

Descriptive, comparative and regression analyses will be used to investigate the impact of WASH and food safety on child stunting. Factors relating to WASH and food safety amenities and practices will be considered simultaneously as external risks or preventive factors influencing the incidence and severity of stunting, using multivariable statistical approaches. To relate the main exposure variables

(WASH and food safety factors) with the main outcome (stunting, measured as either z-scores or presence/absence), multivariable regressions models will be developed. According to our causal framework presented in Figure 1, we will adjust this model by potential confounding factors (including age, sex, socioeconomic status and environmental hygiene levels), and we will avoid intermediate factors (e.g., infection rates). Subsequently, we will explore the potential role of the mediators in the causal framework using mediation analysis. Due to the existence of repeated measures conducted over three different timepoints for each child, we will account for this with a random effect term by individual in the regression model. The model will be a linear regression for stunting measured as a continuous variable, and a logistic (or ordered logistic) regression for stunting (moderate and severe) measured as a dichotomous variable. Due to the fact that in our 3 country settings the relevant association might differ, we will estimate the models separately in each country, and afterwards we will conduct individual participant data meta-analysis of the coefficients of interest across the countries. This strategy will allow for an optimal fit of the model in each country and at the same time we will be able to explore the heterogeneity of coefficients across countries and within sub-groups (by age, sex and socioeconomic characteristics).

Data triangulation will also be used, firstly, to address biases in individual data collection tools. For example, known biases occur when measures are reported by participants, particularly by those people for whom illness becomes normalised after repeated bouts of infection[27]. Observation of behaviours, through enumerator presence in the household at certain times of day, can help to mitigate reporting bias. However, surveys and observation may also affect participant motivation, particularly when repeated over long periods. Extensive collection of samples for laboratory testing can be done, but have their own weaknesses [28]. Secondly, data triangulation through statistical modelling will be used to determine the relationship between WASH and food safety in each of the study contexts, and to explore relevant questions such as whether they are complements or substitutes in child health and nutrition. As such, a mix of approaches is being used in this study to address these potential challenges.

Patient and Public Involvement statement

Study participants have not been involved in the general design of the study. However, the study was designed in partnership with local research partners and stakeholders with experience in working at each of the settings. Local enumerators helped finalise the data collection tools during training and pilot testing.

Extensive consultations will be organised with the local communities to understand the ethos of the research including the involvement of participants and the implications of the research. Question and answer sections will be held during the consulations to answer any questions and/or concerns raised. Study participants and the public will be involved in the dissemination of the study's findings through community discussion or engagement events.

DISCUSSION

Poor food safety and inadequate WASH, including getting in direct contact with animals, their produce and/or their faeces, are thought to be important contributors to undernutrition. The potential mechanisms include nutrient loss (e.g., during diarrhoeal episodes), energy used by the immune system (e.g., in combating illnesses), and increased intestinal permeability and reduction in nutrients absorption (e.g., enteropathy) [29]. Other indirect pathways include scarcity of income and time with which undertake childcare and feeding practices. Cumulatively over the first two years of life, these

Confidential: For Review Only

factors can contribute to stunting expressed as linear growth faltering. However, the causes of infant stunting are not exclusive to these factors, and therefore it is critical to cohesively consider the multiple determinants involved. The UKRI GCRF AASH is using an interdisciplinary approach to investigate this variety of factors causing stunting with the aim to identify solutions for the ameriolation of growth and development in early childhood at the 'whole-child' level. Within such an interdisciplinary framework, this study design will generate comprehensive evidence on the contribution of WASH, and food safety at the household level.

Challenges to conducting integrated assessment in WASH and food safety in a multi-country study like this are various, including identifying key ASFs and key hazards in a systematic way, or the analysis of pathways linking hygiene, food and water safety, and sanitation data into the typologies of stunting. For this purpose, we developed holistic data collection tools relevant to each study setting, to measure risk factors for exposure to food- and water-borne disease. Multiple household-level interventions are available in both WASH and food safety but, to date, their effectiveness has not been assessed together, despite the similarity in the exposure mechanisms they are targeting. The effectiveness of relavant interventions and technologies in addressing malnutrition depends on both their biological efficacy, and the degree to which consumer behaviour adapts to accept and use the new approach, in the environment where it is based[30]. We draw on an approach and suggest data collection tools through which these various underlying and proximate determinants of child growth may be analysed.

Additionally, the value chains for ASF generally present important potential to improve availability, accessibility, affordability and safety for the most nutritionally vulnerable population groups[31]. This study will also provide information that will help to identify entry points in chains where upgrading may be feasible and welcome by stakeholders (e.g., consumers, government, and businesses). Based on secondary dietary and nutritional data, a systematic literature review of food safety hazards in ASF value chains in each study setting and local experts' feedback, commodities of likely relevance are eggs in all countries, milk in India and Senegal, and fish in Indonesia (see AASH food system protocol description in Cooper et al in this supplement for value chain work).

The data generated from the study will help pave the way for future interventions to to manage factors associated with and prevent stunting.

ETHICS AND DISSEMINATION

Ethical approval for the study was granted by the Institutional ethics committees of the London School of Hygiene and Tropical Medicine (17915/RR/17513), the Social Science Research Ethical Review Board at the Royal Veterinary College (URN SR2020-0198) and the International Livestock Research Institute Institutional Research Ethics Committee (ILRI-IREC2020-33). In-country approvals were also granted: the National Institute of Nutrition (ICMR), Ministry of Health and Family Welfare, Government of India (CR/04/I/2021), the Health Research Ethics Committee, University of Indonesia and Dr Cipto Mangunkusumo Hospital (KET-887/UN2.F1/ETIK/PPM.00.02/2019), and the Comité National d'Ethique pour la Recherche en Santé, Senegal (Protocole SEN19/78).

Findings of the study will be disseminated through publication in peer-reviewed journals, presented at relevant international conferences, public engagement events, and policy-maker and stakeholder events. Data generated from the study will be deposited on publicly available data repository.

Confidential: For Review Only

REFERENCES

1. Checkley W, Buckley G, Gilman RH, et al. Multi-country analysis of the effects of diarrhoea on childhood stunting. *International journal of epidemiology* 2008;37(4):816-30.

2. Black RE, Cousens S, Johnson HL, et al. Global, regional, and national causes of child mortality in 2008: a systematic analysis. *The lancet* 2010;375(9730):1969-87.

3. Liu L, Johnson HL, Cousens S, et al. Global, regional, and national causes of child mortality: an updated systematic analysis for 2010 with time trends since 2000. *The lancet* 2012;379(9832):2151-61.

4. Havelaar AH, Kirk MD, Torgerson PR, et al. World Health Organization Global Estimates and Regional Comparisons of the Burden of Foodborne Disease in 2010. *PLOS Medicine* 2015;12(12):e1001923. doi: 10.1371/journal.pmed.1001923

5. White GF, Bradley DJ, White AU. Drawers of water: domestic water use in East Africa. *Bulletin of the World Health Organization* 1972;80(1):63-73.

6. Motarjemi Y, Käferstein F, Moy G, et al. Contaminated weaning food: a major risk factor for diarrhoea and associated malnutrition. *Bulletin of the World health Organization* 1993;71(1):79.

7. Touré O, Coulibaly S, Arby A, et al. Piloting an intervention to improve microbiological food safety in Peri-Urban Mali. *International journal of hygiene and environmental health* 2013;216(2):138-45. doi: https://doi.org/10.1016/j.ijheh.2012.02.003

8. Kinkese DM, Hang'ombe MB, Toure O, et al. Contamination of complementary weaning foods for children with Escherichia coli and salmonella species in Lusaka District, Zambia. *Journal of Preventive and Rehabilitative Medicine* (1):19-31. doi: Doi:10.21617/jprm.2018.0101.3

9. Gautam OP, Schmidt W-P, Cairncross S, et al. Trial of a novel intervention to improve multiple food hygiene behaviors in Nepal. *The American journal of tropical medicine and hygiene* 2017;96(6):1415.

10. Wright J, Gundry S, Conroy R. Household drinking water in developing countries: a systematic review of microbiological contamination between source and point-of-use. *Tropical medicine & international health* 2004;9(1):106-17.

11. Lule JR, Mermin J, Ekwaru JP, et al. Effect of home based water chlorination and safe storage on diarrhea among persons with human immonodeficiency virus in Uganda. *American Journal of Tropical Medicine & Hygiene* 2005;73:926–33.

12. Curtis V. Hygiene: how myths, monsters, and mothers-in-law can promote behaviour change. *Journal of Infection* 2001;43(1):75-79.

13. Chirgwin H, Cairncross S, Zehra D, et al. Interventions promoting uptake of water, sanitation and hygiene (WASH) technologies in low-and middle-income countries: An evidence and gap map of effectiveness studies. *Campbell Systematic Reviews* 2021;17(4):e1194.

14. Unicef. Strategy for improved nutrition of children and women in developing countries. A UNICEF policy review. New York: UNICEF 1990.

15. Unnevehr L, Grace D. Aflatoxins: Finding solutions for improved food safety. Washington, DC: IFPRI 2013.

16. Penakalapati G, Swarthout J, Delahoy MJ, et al. Exposure to animal feces and human health: a systematic review and proposed research priorities. *Environmental science & technology* 2017;51(20):11537-52.

Confidential: For Review Only

17. Majorin F, Torondel B, Chan GKS, et al. Interventions to improve disposal of child faeces for preventing diarrhoea and soil-transmitted helminth infection. *Cochrane Database of Systematic Reviews* 2019(9)

18. Prendergast AJ, Gharpure R, Mor S, et al. Putting the "A" into WaSH: a call for integrated management of water, animals, sanitation, and hygiene. *The Lancet Planetary Health* 2019;3(8):e336-e37. doi: https://doi.org/10.1016/S2542-5196(19)30129-9

19. Aunger R, Curtis V. Behaviour Centred Design: towards an applied science of behaviour change. *Health psychology review* 2016;10(4):425-46. doi: http://dx.doi.org/10.1080/17437199.2016.1219673

20. Popkin BM, Solon FS. Income, time, the working mother and child nutriture. *Journal of tropical pediatrics* 1976;22(4):156-66.

21. Cairncross S, Cuff J. Water use and health in Mueda, Mozambique. *Transactions of the Royal Society of Tropical Medicine and Hygiene* 1987;81(1):51-54.

22. Iannotti L, Tarawali SA, Baltenweck I, et al. Livestock-derived foods and sustainable healthy diets. 2021

23. Iannotti LL, Lutter CK, Stewart CP, et al. Eggs in early complementary feeding and child growth: a randomized controlled trial. *Pediatrics* 2017;140(1)

24. Headey D, Hirvonen K, Hoddinott J. Animal sourced foods and child stunting: Wiley Online Library, 2018.

25. De Beer H. Dairy products and physical stature: a systematic review and meta-analysis of controlled trials. *Economics & Human Biology* 2012;10(3):299-309.

26. Neumann CG, Murphy SP, Gewa C, et al. Meat supplementation improves growth, cognitive, and behavioral outcomes in Kenyan children. *the Journal of Nutrition* 2007;137(4):1119-23.

27. Briscoe J, Feachem RG, Rahaman MM. Evaluating health impact: water supply, sanitation, and hygiene education: IDRC, Ottawa, ON, CA 1986.

28. Goddard FG, Ban R, Barr DB, et al. Measuring environmental exposure to enteric pathogens in low-income settings: review and recommendations of an interdisciplinary working group. *Environmental science & technology* 2020;54(19):11673-91. doi: DOI: 10.1021/acs.est.0c02421

29. Bourke CD, Berkley JA, Prendergast AJ. Immune dysfunction as a cause and consequence of malnutrition. *Trends in immunology* 2016;37(6):386-98.

30. Cumming O, Arnold BF, Ban R, et al. The implications of three major new trials for the effect of water, sanitation and hygiene on childhood diarrhea and stunting: a consensus statement. *BMC medicine* 2019;17(1):1-9. doi: 10.1186/s12916-019-1410-x

31. Alarcon P, Dominguez-Salas P, Fèvre EM, et al. The importance of a food systems approach to low and middle income countries and emerging economies: a review of theories and its relevance for disease control and malnutrition. *Frontiers in Sustainable Food Systems* 2021:92. doi: https://doi.org/10.3389/fsufs.2021.642635

Confidential: For Review Only

AUTHORS CONTRIBUTIONS

All authors were involved in the study design and the preparation of the study tools and protocols at different stages; BH, HW and PDS drafted the manuscript sections; all other authors critically reviewed the manuscript. PDS and HW share first authorship.

We want to thank Alessia Gasco for producing the conceptual framework and Claire Heffernan, Kaitlin Conway and Modou Jobarteh for careful editing of the manuscript.

FUNDING STATEMENT

This work was supported by the UKRI-GCRF Action Against Stunting Hub (Project ref. MR/S01313X/1). The views expressed in this manuscript are those of the authors and do not necessarily represent the views of the funders.

COMPETING INTEREST STATEMENT

The authors declare no conflict of interest.

Confidential: For Review Only

APPENDIX 1: WASH AND FOOD SAFETY QUESTIONNAIRE

WATER SANITATION *(3 TIMES AT 3RD TRIMESTER, 3 & 24 MO)*
**ws_1. What is the main source of drinking water for members of your household?**
Piped waterpiped to dwelling
Piped waterpiped to compound yard or plot
Piped waterpiped to neighbor
Public tap / tapstand
Borehole or tubewell
Protected well
Unprotected well
Protected spring
Unprotected spring
Rainwater
Delivered watertanker or truck
Delivered watersmall cart with tank drum
Water kiosk
Packaged waterbottled water
Packaged watersachet water
Surface water
Other: specify
ws_1_96x. Other: specify
**ws_2. What is the main source of water used by members of this household for other purposes, such as cooking and handwashing?**
Piped waterpiped to dwelling
Piped waterpiped to compound yard or plot
Piped waterpiped to neighbor
Public tap / tapstand
Borehole or tubewell
Protected well
Unprotected well
Protected spring
Unprotected spring
Rainwater
Delivered watertanker or truck
Delivered watersmall cart with tank drum
Water kiosk
Packaged waterbottled water
Packaged watersachet water
Surface water
Other: specify
ws_2_96x. Other: specify
**ws_3. Where is this water collected from?**
In own dwelling
In own yard

Confidential: For Review Only

Elsewhere
**ws_4. How many minutes in total does it take you to go fetch water from this source and come back? (Fill in X minutes)**
**ws_5. Do you store drinking water in your home?**
Yes
No
**ws_6. Where do you store your drinking water?**
In a container with a lid/top on it
In an open container
Container on the roof
**ws_7. In the last 4 weeks/month, how frequently did you or anyone in your household worry you would not have enough water for all of your household needs?**
Never (0 times)
Rarely (1-2 times)
Sometimes (3-10 times)
Often (11-20times)
Always (more than 20 times)
**ws_8. In the last 4 weeks/month, how frequently has your main water source been interrupted or limited (eg water pressure, less water than expected, river dried up)?**
Never (0 times)
Rarely (1-2 times)
Sometimes (3-10 times)
Often (11-20times)
Always (more than 20 times)
**ws_9. In the last 4 weeks/month, how frequently has there not been enough water to wash clothes?**
Never (0 times)
Rarely (1-2 times)
Sometimes (3-10 times)
Often (11-20times)
Always (more than 20 times)
**ws_10. In the last 4 weeks/month, how frequently have you or anyone in your household had to change schedules or plans due to problems with your water situation? Activities that may have been interrupted include caring for others, doing household chores, agricultural work, income-generating activities, sleeping, etc**
Never (0 times)
Rarely (1-2 times)
Sometimes (3-10 times)
Often (11-20times)
Always (more than 20 times)
**ws_11. In the last 4 weeks/month, how frequently have you or anyone in your household had to change what was being eaten because there were problems with water (eg, for washing foods, cooking, etc)?**
Never (0 times)
Rarely (1-2 times)
Sometimes (3-10 times)
Often (11-20times)

Confidential: For Review Only

Always (more than 20 times)
**ws_12. In the last 4 weeks/month, how frequently have you or anyone in your household had to go without washing hands after dirty activities (eg, defecating or changing diapers, cleaning animal dung) because of problems with water?**
Never (0 times)
Rarely (1-2 times)
Sometimes (3-10 times)
Often (11-20times)
Always (more than 20 times)
**ws_13. In the last 4 weeks/month, how frequently have you or anyone in your household had to go without washing their body because of problems with water (eg, not enough water, dirty, unsafe)?**
Never (0 times)
Rarely (1-2 times)
Sometimes (3-10 times)
Often (11-20times)
Always (more than 20 times)
**ws_14. In the last 4 weeks/month, how frequently has there not been as much water to drink as you would like for you or anyone in your household?**
Never (0 times)
Rarely (1-2 times)
Sometimes (3-10 times)
Often (11-20times)
Always (more than 20 times)
**ws_15. In the last 4 weeks/month, how frequently did you or anyone in your household feel angry about your water situation?**
Never (0 times)
Rarely (1-2 times)
Sometimes (3-10 times)
Often (11-20times)
Always (more than 20 times)
**ws_16. In the last 4 weeks/month, how frequently have you or anyone in your household gone to sleep thirsty because there wasn't any water to drink?**
Never (0 times)
Rarely (1-2 times)
Sometimes (3-10 times)
Often (11-20times)
Always (more than 20 times)
**ws_17. In the last 4 weeks/month, how frequently has there been no useable or drinkable water whatsoever in your household?**
Never (0 times)
Rarely (1-2 times)
Sometimes (3-10 times)
Often (11-20times)
Always (more than 20 times)
**ws_18. In the last 4 weeks/month, how frequently have problems with water caused you or anyone in your household to feel ashamed/excluded/stigmatized?**

Confidential: For Review Only

Never (0 times)
Rarely (1-2 times)
Sometimes (3-10 times)
Often (11-20times)
Always (more than 20 times)
**ws_19. Is there a latrine or toilet that members of this household use regularly?**
Yes
No
**ws_20. Where is this toilet facility located?**
In own dwelling
In own yard or plot
Elsewhere
**ws_21. How many households other than your own share this facility?**
Enter the enumerator Name
**fh_1. Is there a designated place for handwashing in the household?**
Yes
No
**fh_2. How often is soap available at this location?**
Always
Sometimes
Never
**fh_3. What were all of the times when you washed your hands (Circle all that apply but do not prompt After each response, ask "any other times?")**
After defecating
After cleaning a child/wiping a child's bottom
After changing a babies nappy
Before preparing food
Before eating
Before feeding the child
After touching animals
After touching animal faeces
After handling raw animal source foods
After crop farming activities
Other
Cannot remember/does not know
fh_3_96x. Specify other
**FOOD STORAGE**
**fh_4. Do you store meat and meat products (except dried meat)?**
Yes
No
**fh_4a. Where do you store raw meat (fresh/chilled/frozen)?**
In a fridge
In a freezer
Covered in a cool room (below 5 degrees Celsius)

Confidential: For Review Only

Uncovered in a cool room (below 5 degrees Celsius)
Covered in a room at room temperature
Uncovered in a room at room temperature
Other, specify
fh_4a_96x. Other, specify
**fh_4b. How long do you store the meat and meat products?**
less than 2 hours
2 hours to 5 hours
5 hours to 12 hours
12 hours
1-3 days
3-7 days
1 week
1 month
**fh_4c. Where do you store processed meat (ready-to-cook and ready-to-eat)?**
In a fridge
In a freezer
Covered in a cool room (below 5 degrees Celsius)
Uncovered in a cool room (below 5 degrees Celsius)
Covered in a room at room temperature
Uncovered in a room at room temperature
Other, specify
fh_4c_96x. Other, specify
**fh_4d. How long do you store the meat and meat products?**
less than 2 hours
2 hours to 5 hours
5 hours to 12 hours
12 hours
1-3 days
3-7 days
1 week
1 month
**fh_5. Do you store milk and milk products (except milk powder, evaporated milk or condensed milk)?**
Yes
No
**fh_5a. Where do you store liquid milk (including pasteurized and non-pasteurized)?**
In a fridge
In a freezer
Covered in a cool room (below 5 degrees Celsius)
Uncovered in a cool room (below 5 degrees Celsius)
Covered in a room at room temperature
Uncovered in a room at room temperature
Other, specify
fh_5a_96x. Other, specify

Confidential: For Review Only

**fh_5b. Where do you store solid/semi solid dairy/milk products (e.g cheese, yoghurt, ghee, etc)?**
In a fridge
In a freezer
Covered in a cool room (below 5 degrees Celsius)
Uncovered in a cool room (below 5 degrees Celsius)
Covered in a room at room temperature
Uncovered in a room at room temperature
Other, specify
fh_5b_96x. Other, specify
**fh_6. Do you store raw or cooked vegetables and fruits?**
Yes
No
**fh_6a. Where do you store raw vegetables?**
In a fridge
In a freezer
Covered in a cool room (below 5 degrees Celsius)
Uncovered in a cool room (below 5 degrees Celsius)
Covered in a room at room temperature
Uncovered in a room at room temperature
Other, specify
fh_6a_96x. Other, specify
**fh_6b. Where do you store cooked vegetables and fruit?**
In a fridge
In a freezer
Covered in a cool room (below 5 degrees Celsius)
Uncovered in a cool room (below 5 degrees Celsius)
Covered in a room at room temperature
Uncovered in a room at room temperature
Other, specify
fh_6b_96x. Other, specify
**fh_7. Do you store food that has been prepared for your child to give it to the child later?**
Yes
No
**fh_7a. How do you store food that has been prepared for your child?**
Thermos
Jug
Plate or bowl (covered)
Plate or bowl (uncovered)
Cooking pot (covered)
Cooking pot (uncovered)
Other container (covered)
Other container (uncovered)
Other, specify
fh_7a_96x. Other, specify

Confidential: For Review Only

**fh_7b. Where do you store this food?**
In a fridge
In a freezer
In a cool room (below 5 degrees Celsius)
In a room at room temperature
Other, specify
fh_7b_96x. Other, specify
**How long do you store this food?**
less than 1 hour
1-2 hours
2 hours to 5 hours
5 hours to 12 hours
12 hours
1-3 days
3-7 days
Longer than a week
Animals
**fh_8. Does your household keep any animals inside or outside the house you are looking after (i.e. not taken care of by others)?**
Yes
No
**fh_9. What type of animals?**
Chickens
Ducks
Other birds
Dogs
Cats
Goats
Sheep
Pigs
Cows
Fish
Other animals, specify
fh_9_96x. Other: specify
**fh_10. Which of these animals are in the patio, veranda or house any time of the day?**
None
Chickens
Ducks
Other birds
Dogs
Cats
Goats
Sheeps
Pigs
Cows

Confidential: For Review Only

Other animals, specify
fh_10_96x. Other: specify
**fh_10a. Is the animal contained in some form of enclosure?**
Yes
No
**fh_10b. Can the child be in contact with the animals or their faeces?**
Yes
No
**fh_10c. Can the animal be in contact with food for the child?**
Yes
No
**ASF1_1. What is the first priority ASF consumed by the child most recently? Select [ASF1]**
Chicken
fish
beef
egg
squid
meat ball
beef soup
chicken soup
duck egg
fish egg
None
**ASF1_2. How was this [ASF] packaged when you got it from the source? (select all that apply)**
No packaging
Sealed package
Washable container (plastic, glass, steel), open
Washable container (plastic, glass, steel), closed
Plastic bag
Newspaper
Other paper
Aluminium foil
Other foil
Container made from natural materials
Other, specify
ASF1_2_96x. ASF_2_96x Other: specify
**ASF1_3. Did you transport this [ASF] after you got it?<br>No transport means that they either produced it themselves or the ASF was brought to them (e.g. by a trader or neighbour, etc)**
Yes
No
**ASF1_3a. How long did you transport the ASF for (to take it home)?**
less than 5 minutes
5 min to 15 min
15 to 30 min

Confidential: For Review Only

30 min to 1 hour
1hour to 2 hours
2 hours to 3 hours
3 hours
**ASF1_3b. How did you transport this [ASF] after you got it?**
Chilled (e.g. on ice)
At ambient temperature
Kept warm (e.g. thermos or thermal food container)
Other, specify
ASF1_3b_96x. Other, specify
**ASF1_4. Did you store this [ASF] before preparation?**
Yes
No
**ASF1_4a. Was this [ASF] covered when you stored it before preparation?**
Yes
No
**ASF1_4b. Where did you store this [ASF] before preparation?**
In a fridge
In a freezer
In a cool room (below 5 degrees Celsius)
In a room at room temperature
Other, specify
ASF1_4b_96x. Specify, other
**ASF1_4c. How long was this [ASF] stored before preparing?**
minute
hour
day
total minutes
total hours
total day
**ASF1_5. Did you wash this [ASF] before preparation?**
Yes
No
Not applicable
**ASF1_6. If yes, why did you wash this [ASF]? (multiple answers)**
To remove visible dirt
To remove visible blood
To make it safer
Because it is a good practice
Other, specify
ASF1_6_96x. Specify, other
**ASF1_7. Did you chop or cut this [ASF] product before preparation?**
Yes
No

Confidential: For Review Only

Not applicable
**ASF1_8. If yes, which tool(s) did you use to chop or cut this [ASF]? (multiple answers)**
Cutting board
Plate
Knife
Other, specify
ASF1_8_96x. Specify, other
**ASF1_9. How did you prepare this food for the child?**
Grilling
Baking
Frying
Boiling
Stewing
Simmering
Fermenting
Heating
Mixed in with other ingredients without cooking the ASF (e.g. mixing yoghurt with fruit or adding dried fish to salad)
Made smaller portion (e.g. cutting in pieces or butting in a bowl), but did not cook or mix with other ingredients
Other, specify
ASF1_9_96x. Specify, other
**ASF1_10. For how long was this [ASF] cooked?**
1-5 min
5-15 min
15-30 min
30-60 min
More than 1 hour
**ASF1_11. After preparation, was this [ASF] stored before giving it to the child?**
Yes
No
**ASF1_12. After preparation, how long was this [ASF] stored before giving it to the child?**
1-5 min
5-15 min
15-30 min
30-60 min
More than 1 hour, less than 24h
More than 1 day, less than 2 days
More than 2 days
**ASF1_13. How did you store this [ASF] that had been prepared for your child?**
Thermos
Jug
Plate or bowl (covered)
Plate or bowl (uncovered)
Cooking pot (covered)

Confidential: For Review Only

Cooking pot (uncovered)
Other container (covered)
Other container (uncovered)
Other, specify
ASF1_13_96x. Specify, other
**ASF1_14. Where did you store this [ASF]?**
In a fridge
In a freezer
In a cool room (below 5 degrees Celsius)
In a room at room temperature
Other, specify
ASF1_14_96x. Specify other
**ASF2_1. What is the second priority ASF consumed by the child most recently? Select [ASF1]**
Chicken
fish
beef
egg
squid
meat ball
beef soup
chicken soup
duck egg
fish egg
None
**ASF2_2. How was this [ASF] packaged when you got it from the source? (select all that apply)**
No packaging
Sealed package
Washable container (plastic, glass, steel), open
Washable container (plastic, glass, steel), closed
Plastic bag
Newspaper
Other paper
Aluminium foil
Other foil
Container made from natural materials
Other, specify
ASF_2_96x Other: specify
**ASF2_3. Did you transport this [ASF] after you got it?** **No transport means that they either produced it themselves or the ASF was brought to them (e.g. by a trader or neighbour, etc)**
Yes
No
**ASF2_3a. How long did you transport the ASF for (to take it home)?**
less than 5 minutes
5 min to 15 min

Confidential: For Review Only

15 to 30 min
30 min to 1 hour
1hour to 2 hours
2 hours to 3 hours
3 hours
**ASF2_3b. How did you transport this [ASF] after you got it?**
Chilled (e.g. on ice)
At ambient temperature
Kept warm (e.g. thermos or thermal food container)
Other, specify
ASF2_3b_96x. Other specify
**ASF2_4. Did you store this [ASF] before preparation?**
Yes
No
**ASF2_4a. Was this [ASF] covered when you stored it before preparation?**
Yes
No
**ASF2_4b. Where did you store this [ASF] before preparation?**
In a fridge
In a freezer
In a cool room (below 5 degrees Celsius)
In a room at room temperature
Other, specify
ASF2_4b_96x. Specify, other
**ASF2_4c. How long was this [ASF] stored before preparing?**
minute
hour
day
total minutes
total hours
total day
**ASF2_5. Did you wash this [ASF] before preparation?**
Yes
No
Not applicable
**ASF2_6. If yes, why did you wash this [ASF]? (multiple answers)**
To remove visible dirt
To remove visible blood
To make it safer
Because it is a good practice
Other, specify
ASF2_6_96x. Specify, other
**ASF2_7. Did you chop or cut this [ASF] product before preparation?**
Yes

Confidential: For Review Only

No
Not applicable
**ASF2_8. If yes, which tool(s) did you use to chop or cut this [ASF]? (multiple answers)**
Cutting board
Plate
Knife
Other, specify
ASF2_8_96x. Specify, other
**ASF2_9. How did you prepare this food for the child?**
Grilling
Baking
Frying
Boiling
Stewing
Simmering
Fermenting
Heating
Mixed in with other ingredients without cooking the ASF (e.g. mixing yoghurt with fruit or adding dried fish to salad)
Made smaller portion (e.g. cutting in pieces or butting in a bowl), but did not cook or mix with other ingredients
Other, specify
ASF2_9_96x. Specify, other
**ASF2_10. For how long was this [ASF] cooked?**
1-5 min
5-15 min
15-30 min
30-60 min
More than 1 hour
**ASF2_11. After preparation, was this [ASF] stored before giving it to the child?**
Yes
No
**ASF2_12. After preparation, how long was this [ASF] stored before giving it to the child?**
1-5 min
5-15 min
15-30 min
30-60 min
More than 1 hour, less than 24h
More than 1 day, less than 2 days
More than 2 days
**ASF2_13. How did you store this [ASF] that had been prepared for your child?**
Thermos
Jug
Plate or bowl (covered)
Plate or bowl (uncovered)

Confidential: For Review Only

Cooking pot (covered)
Cooking pot (uncovered)
Other container (covered)
Other container (uncovered)
Other, specify
ASF2_13_96x. Specify, other
**ASF2_14. Where did you store this [ASF]?**
In a fridge
In a freezer
In a cool room (below 5 degrees Celsius)
In a room at room temperature
Other, specify
ASF2_14_96x. Specify, other
**ASF3_1. What is the third priority ASF consumed by the child most recently? Select [ASF1]**
Chicken
fish
beef
egg
squid
meat ball
beef soup
chicken soup
duck egg
fish egg
None
**ASF3_2. How was this [ASF] packaged when you got it from the source? (select all that apply)**
No packaging
Sealed package
Washable container (plastic, glass, steel), open
Washable container (plastic, glass, steel), closed
Plastic bag
Newspaper
Other paper
Aluminium foil
Other foil
Container made from natural materials
Other, specify
ASF_2_96x Other: specify
**ASF3_3. Did you transport this [ASF] after you got it?**<br>**No transport means that they either produced it themselves or the ASF was brought to them (e.g. by a trader or neighbour, etc)**
Yes
No
**ASF3_3a. How long did you transport the ASF for (to take it home)?**
less than 5 minutes

Confidential: For Review Only

5 min to 15 min
15 to 30 min
30 min to 1 hour
1hour to 2 hours
2 hours to 3 hours
3 hours
**ASF3_3b. How did you transport this [ASF] after you got it?**
Chilled (e.g. on ice)
At ambient temperature
Kept warm (e.g. thermos or thermal food container)
Other, specify
ASF3_3b_96x. Other, specify
**ASF3_4. Did you store this [ASF] before preparation?**
Yes
No
**ASF3_4a. Was this [ASF] covered when you stored it before preparation?**
Yes
No
**ASF3_4b. Where did you store this [ASF] before preparation?**
In a fridge
In a freezer
In a cool room (below 5 degrees Celsius)
In a room at room temperature
Other, specify
ASF3_4b_96x. Specify, other
**ASF3_4c. How long was this [ASF] stored before preparing?**
minutes
hour
day
total minutes
total hours
total day
**ASF3_5. Did you wash this [ASF] before preparation?**
Yes
No
Not applicable
**ASF3_6. If yes, why did you wash this [ASF]? (multiple answers)**
To remove visible dirt
To remove visible blood
To make it safer
Because it is a good practice
Other, specify
ASF3_6_96x. Specify, other
**ASF3_7. Did you chop or cut this [ASF] product before preparation?**

Confidential: For Review Only

Yes
No
Not applicable
**ASF3_8. If yes, which tool(s) did you use to chop or cut this [ASF]? (multiple answers)**
Cutting board
Plate
Knife
Other, specify
ASF3_8_96x. Specify, other
**ASF3_9. How did you prepare this food for the child?**
Grilling
Baking
Frying
Boiling
Stewing
Simmering
Fermenting
Heating
Mixed in with other ingredients without cooking the ASF (e.g. mixing yoghurt with fruit or adding dried fish to salad)
Made smaller portion (e.g. cutting in pieces or butting in a bowl), but did not cook or mix with other ingredients
Other, specify
ASF3_9_96x. Specify, other
**ASF3_10. For how long was this [ASF] cooked?**
1-5 min
5-15 min
15-30 min
30-60 min
More than 1 hour
**ASF3_11. After preparation, was this [ASF] stored before giving it to the child?**
Yes
No
**ASF3_12. After preparation, how long was this [ASF] stored before giving it to the child?**
1-5 min
5-15 min
15-30 min
30-60 min
More than 1 hour, less than 24h
More than 1 day, less than 2 days
More than 2 days
**ASF3_13. How did you store this [ASF] that had been prepared for your child?**
Thermos
Jug
Plate or bowl (covered)

Confidential: For Review Only

Plate or bowl (uncovered)
Cooking pot (covered)
Cooking pot (uncovered)
Other container (covered)
Other container (uncovered)
Other, specify
ASF3_13_96x. Specify, other
**ASF3_14. Where did you store this [ASF]?**
In a fridge
In a freezer
In a cool room (below 5 degrees Celsius)
In a room at room temperature
Other, specify
ASF3_14_96x. Other, specify

Confidential: For Review Only

APPENDIX 2: WASH AND FOOD SAFETY SPOT CHECK

WATER SANITATION (SPOT CHECK) *(3 TIMES AT 2ND TRIMESTER, 3 & 9MO)*
**wash_1. What type of facility is the primary location for defecation?**
Flush toilet to piped sewer system
Flush toilet to septic tank
Flush toilet to pit
Flush toilet to onsite, above ground, open pit
Pour flush toilet to piped sewer system
Pour flush toilet to septic tank
Pour flush toilet to pit
Pour flush toilet to onsite, above ground, open pit
Pit latrine with concrete slab (not pour flush)
Pit latrine without slab (not pour flush)
Bucket
Bag
Open defecation (no facilities)
Other:
Refused observation
wash_1_96x. Specify, other
**wash_2. Approximately how many meters is the toilet from the entrance of the household**
**wash_3. For the following, please note if following indicators of functionality are present**
**wash_3_1. Is the pan working (not broken)**
yes
no
**wash_3_2. Are there leaves or rubbish blocking the pan?**
yes
no
**wash_3_3. Is there standing water in the pan?**
yes
no
**wash_3_4. Is there a cover over the pit?**
yes
no
**wash_3_5. Are there one or two pits?**
One
Two
More
**wash_3_6. Is there a cover on all the pits?**
No
Partial (only to one pit)
Yes, to more than one pit
**wash_3_7. Are there flies?**
None

Confidential: For Review Only

Some
Many
**wash_4. Can you please show me where members of this household most often wash their hands?**
Fixed facility (sink/basin)… in dwelling
Fixed facility….in yard
Mobile object (bucket / jug)
No handwashing place in dwelling
Refused observation
**wash_5. Is there water available at this location?**
Yes
No
**wash_6. Is soap available at this location? (Soap includes bar soap, liquid soap, powder detergent, and soapy water but does not include ash, soil, sand or other handwashing agents)**
Yes
No
**wash_7. Is the dedicated location located near (within 5 steps) the primary cooking location?**
Yes
No
**wash_8. Is the dedicated location located near (within 5 steps) the latrine/toilet**
Yes
No
**wash_9. Are there animals located in or around the household? (select all)**
No animals visible
In the cooking area (outside house)
In the cooking area (inside house)
In the food storing area (outside house)
In the food storing area (inside house)
In other areas of the house that are not cooking or food storing areas
**wash_10. What type of animals (select all)**
Chickens
Ducks
Other birds
Dog
Cat
Goat
Sheep
Pig
Cow
Wild animals
Rodents
Other animal
wash_10_96x. Other, specify
**wash_11. Are there animal feces located in or around the household? (select all)**
No animal feces visible
In the cooking area (outside house)

Confidential: For Review Only

In the cooking area (inside house)
In the food storing area (outside house)
In the food storing area (inside house)
In other areas of the house that are not cooking or food storing areas
In the yard / courtyard
FOOD SAFETY QUESTIONNAIRE *(1 TIME: AT 6 MO)*
HAND WASHING
**spot_1. Can you please show me where members of this household most often wash their hands after using the toilets?**
Fixed facility (sink/basin)… in dwelling
Fixed facility….in yard
Mobile object (bucket / jug)
No handwashing place in dwelling
Refused observation
**spot_2. Is there water available at this location?**
Yes
No
**spot_3. Is soap available at this location? (Soap includes bar soap, liquid soap, powder detergent, and soapy water but does not include ash, soil, sand or other handwashing agents)**
Yes
No
**spot_4. Is the dedicated location located near (within 5 steps) the primary cooking location?**
Yes
No
**spot_5. Is the dedicated location located near (within 5 steps) the latrine/toilet**
Yes
No
**spot_6. Can you show me where members of this household most often wash their hands before eating or preparing food?**
Fixed facility (sink/basin)… in dwelling
Fixed facility….in yard
Mobile object (bucket / jug)
No handwashing place in dwelling
Refused observation
Same as after toilet
**spot_7. Is there water available at this location?**
Yes
No
**spot_8. Is soap available at this location? (Soap includes bar soap, liquid soap, powder detergent, and soapy water but does not include ash, soil, sand or other handwashing agents)**
Yes
No
**spot_9. Is the dedicated location located near (within 5 steps) the primary cooking location?**
Yes

Confidential: For Review Only

No
**spot_10. Is the dedicated location located near (within 5 steps) the latrine/toilet**
Yes
No
**spot_11. Do you see animals in or around the household? (select all)**
No animals visible
In the cooking area (outside house)
In the cooking area (inside house)
In the food storing area (outside house)
In the food storing area (inside house)
In other areas of the house that are not cooking or food storing areas
**spot_12. What type of animals (select all)**
Chickens
Ducks
Other birds
Dog
Cat
Goat
Sheep
Pig
Cow
Wild animals
Rodents
Other animal
spot_12_96x. Specify other
**spot_13. Is it possible for the child to be in contact with these animals (e.g. if they are unrestrained or the child can access an enclosure)?**
Yes
No
**spot_14. Is it possible for these animals to be in contact with food (e.g. if animals are unrestrained)**
Yes
No
**spot_15. Are there visible animal feces located in or around the household? (select all)**
No animal feces visible
In the cooking area (outside house)
In the cooking are (inside house)
In the food storing area (outside house)
In the food storing area (inside house)
In other areas of the house that are not cooking or food storing areas
In the yard / courtyard
In the animal enclosure (e.g. chicken coop)
**spot_16. Is it possible for the child to be in contact with these faeces (e.g. because they are in areas where the child is or the child can access an enclosure)?**
Yes
No

Confidential: For Review Only

**spot_17. Is it possible that food gets in contact with these faeces?**
Yes
No
**safe_1. Is there a functioning refrigerator?**
Yes
No
**safe_2. Is there a functioning freezer?**
Yes
No
**safe_3. Is there any visible, stored [ASF] not protected from the environment?**
Yes
No
**safe_4. Is it [ASF] in contact with other food products?**
Yes
No
**safe_5. Is the area where this [ASF] is kept protected from animals and flies?**
Yes
No
**safe_6. Is there a kitchen area (a dedicated food preparation area)?**
Yes
No
**safe_6a. Is the kitchen area free from pest animals (i.e. birds, flies, rats)**
Yes
No
**safe_6b. Are there anti-pest measures in place in the kitchen area? e.g. nets, traps, insect killers**
Yes
No
**safe_6c. Is the kitchen area free of visible dirt?**
Yes
No
**safe_6d. Are cooking utensils clean?**
Yes
No
**safe_6e. Is the kitchen area easy to clean (e.g. tiles)?**
Yes
No
**safe_6f. Does the kitchen area have access to running water?**
Yes
No
**safe_6g. If there is no access to running water, is there stored water in the area where food is being prepared?**
Yes, there is water in a container with a lid/top on it
Yes, there is water in an open container
No, there is no water

Confidential: For Review Only

**safe_6h. Is soap available at this location? (Soap includes bar soap, liquid soap, powder detergent, and soapy water but does not include ash, soil, sand or other handwashing agents)**
Yes
No
**safe_7. Is there a food storage area?**
Yes
No
**safe_7a. In the food storage area, is the storage equipment made of material that is easy to clean (e.g. metal, plastic)?**
Yes
No
**safe_7b. Is the food storage area free from pest animals (i.e. birds, flies, rats)**
Yes
No
**safe_7c. Are there anti-pest measures in place in the food storage area? e.g. nets, traps, insect killers**
Yes
No
**safe_7d. Is the food storage area free of visible dirt?**
Yes
No
**safe_7e. Is the food storage area easy to clean (e.g. tiles)?**
Yes
No
**safe_8. Is there a facility to dispose of food waste?**
No
In the cooking area (outside house)
In the cooking area (inside house)
In the food storing area (outside house)
In the food storing area (inside house)
Outside the house remove from cooking or food storing areas
Removed from the household
**safe_9. Are the knife and chopping board used to cut vegetables and fruits are clean?**
Yes
No
**safe_10.Are the nails of the food handler trimmed?**
Yes
No

Confidential: For Review Only

GCRF Action against stunting Hub

Household ID:	Mother ID:	Child ID:
**Enumerator ID:**	**Supervisor ID:**	
**District:**	**Sub-county:**	
**Parish:**	**Village:**	
**Salter calibration ID:**	**Salter calibration weight:**	

Date of record:	Day of the week today:
**Time ARRIVED:** *(24-hr)*<br><br>**Time DEPARTED:** *(24-hr)*	Monday ☐ Friday ☐<br>Tuesday ☐ Saturday ☐<br>Wednesday ☐ Sunday ☐<br>Thursday ☐

Complete at the end of the day

Describe the location of the observations: **Where in the home the observation was completed – where the observer sat, what the conditions were when you did the observation (weather, lighting, heat) how busy the home was, etc.**

Describe who the main caretaker of the child was:
Mother ☐
Sibling ☐
Grandmother ☐
Aunt ☐
Caretaker ☐
Other, specify ☐ ____

Please note if caretaker changes during observation: Yes ☐ **No** ☐**Mother** ☐
Sibling ☐
Grandmother ☐
Aunt ☐
Caretaker ☐
Other, specify ☐ ____

Direct observation

Household ID: ______

	Yes *(tick)*	Date: *dd/mm/yy*
**Checked by supervisor:** *(code)*		
**Checked by co-ordinator:** *(code)*		
**Data entry completed:** *(code)*		

Activity	___ ____:00				_____ _____:00				___ _____:00				_____ _____:00				Pilot notes
	a	b	c	d	e	f	g	h	l	j	k	l	m	n	o	p	
	00	15	30	45	00	15	30	45	00	15	30	45	00	15	30	45	
1. Child Activities																	
1.1 Sleeping																	
1.2 Caretaker change																	
1.3 Child being cared for by other children																	
2. Child feces																	
2.1 Child nappy or diaper is changed																	
2.2 Child defecates on floor																	
2.3 Child defecates in potty																	
2.4 Child feces disposed in toilet																	
2.5 Child feces disposed in bush / ditch																	
3. Food preparation																	
3.1 Fruit or vegetable washed																	
3.2 Food preparation surface rinsed (specify surface type)																	
3.3 Food preparation surface washed (specify surface type)																	
3.4 Utensils rinsed																	
3.5 Utensils washed																	
3.6 Plates / bowls/ cups rinsed																	
3.7 Plates / bowls/cups washed																	
3.8 Child b ottle rinsed																	
3.9 Child b ottle cleaned																	
4. Food cooking																	Specify from section 3

Confidential: For Review Only

Direct observation

Household ID: _____

Activity	___ ____:00				____ ____:00				___ ____:00				____ ____:00				Pilot notes
	a	b	c	d	e	f	g	h	I	j	k	l	m	n	o	p	
	00	15	30	45	00	15	30	45	00	15	30	45	00	15	30	45	
																	Specify type in comments
4.1 Food fried (ASF / Fruit Veg / Other)																	
4.2 Food dried (ASF / Fruit Veg / other)																	
4.3 Food roasted (ASF / Fruit Veg / other)																	
4.4 Food heated in pot, no boiled (ASF/ Fruit Veg / other)																	
4.5 Food boiled (ASF / Fruit Veg/ other)																	
4.6 Food steamed (ASF / Fruit Veg / Other)																	
4.7 Food arrives processed (ASF / Fruit Veg / Other)																	
4.8 Food added raw / uncooked to dish (ASF / Fruit Veg / Other)																	
5. Food handling																	Specify from section 4
5.1 Cooked food served immediately to child																	
5.2 Cooked food left at room temp / open																	
5.3 Cooked food placed in cabinet / cupboard																	
5.4 Cooked food placed in fridge																	
5.5 Cooked food covered (loosely)																	
5.6 Cooked food covered (sealed)																	
6. Child feeding																	Specify from section 4
6.1 Child breastfed																	
6.2 Child given food that was stored																	
6.3 Child given milk																	
6.4 Child given water																	
6.5 Flies on food given to child																	
6.6 Child fed by caregivers hand																	
6.6 Child eating using child's hand																	
6.7 Child fed/eating using utensils (e.g. spoon)																	
7. Floor contact																	
7.1 Child hand contact with floor																	
7.2 Child hand contact with dirt																	

Direct observation

Household ID: ______

Activity	___ ____:00				____ ____:00				___ ____:00				____ ____:00				Pilot notes
	a	b	c	d	e	f	g	h	I	j	k	l	m	n	o	p	
	00	15	30	45	00	15	30	45	00	15	30	45	00	15	30	45	
7.3 Child eats dirt / soil																	
7.4 Child places objects from the floor in their mouths																	
7.5 Child places hands that have been in contact with the floor in mouth																	
**8. Animal contact**																	Write down the animal (chickens, ducks, other birds, goats, sheep, pigs, cow, dog, cat, other/specify)
8.1 Caretaker hand contact with animals																	
8.2 Caretaker hand contact with animal feces																	
8.3 Child hand contact with animals																	
8.4 Child hand contact with animal feces																	
**9. Toilet use**																	
9.1 Primary caretaker uses toilet																	
9.2 Primary caretaker goes for open defecation																	
**10. Domestic cleaning**																	
10.1 Caretakers washes clothes																	
10.2 Caretaker / other household member sweeps																	
10.3 Caretaker / other household members cleans floors (with disinfectant / soap)																	
10.3 Caretakers handles garbage																	
**11. Handwashing / Facewashing**																	
11.1 Caretaker rines hands – water only																	
11.2 Caretaker washes hands – soap used																	
11.3 Child's hands rinsed – water only																	
11.4 Child's hands washed – soap used																	
11.5 Child's face wiped or rinses																	
11.6 Child's face cleaned																	
11.7 Caretaker bathes																	
11.8 Child bathed																	

Confidential: For Review Only

Direct observation

Household ID: ______

Confidential: For Review Only

Direct observation

Household ID: _____

Data log Table. Food Sample

HH ID: ___________________________

Timepoint: 9 month/ 12 month/ 18 month

Date (DD/MMM/YY): ___________________________

Collection time (24 hour clock, HH:MM): ---___________________________

Collection period: During feeding ☐ Directly after feeding☐ Before feeding☐	
Food:	
Total sample weight	
Ingredients:	
Preparation method:	
Notes:	

Confidential: For Review Only

Direct observation

Household ID: _____

Data log Table. Water Sample

HH ID: ___________________________

Timepoint: 9 month/ 12 month/ 18 month

Date (DD/MMM/YY): ___________________________

Collection time (24 hour clock, HH:MM): ---___________________________

Has the water been treated with chlorine/aquatabs or is from the municipal system?<br>Yes ☐(collect water in a small 100 mL whirlpak bag with sodium thiosulfate tablets + collect further water in a 50mL centrifuge tube)<br>No ☐ (collect water in a large 250 mL whirlpak bag)	
Notes:	

Confidential: For Review Only

Direct observation

Household ID: _____

Data log Table. Surface Sample

Timepoint: 9 month/ 12 month/ 18 month

HH ID: ___________________________

Date (DD/MMM/YY): ___________________________

Collection time (24 hour clock, HH:MM): ---___________________________

Where is the child's food prepared [the surface where foods and utensils are placed during preparation and/or where foods cut up or otherwise processed in any way]?	
Notes:	

Confidential: For Review Only

BMJ Paediatrics Open

Understanding the role of household hygiene practices and foodborne disease risks in child stunting: a UKRI GCRF Action Against Stunting Hub protocol paper

Journal:	*BMJ Paediatrics Open*
Manuscript ID	bmjpo-2022-001695.R2
Article Type:	Protocol
Date Submitted by the Author:	17-Feb-2023
Complete List of Authors:	Dominguez-Salas, Paula; University of Greenwich; International Livestock Research Institute (ILRI)<br>Waddington, Hugh Sharma; London School of Hygiene and Tropical Medicine, Department of Disease Control<br>Grace, Delia; University of Greenwich; International Livestock Research Institute (ILRI)<br>Bosire, Caroline; International Livestock Research Institute (ILRI)<br>Moodley, Arshnee; International Livestock Research Institute (ILRI)<br>Kulkarni, Bharati; National Institute of Nutrition<br>Dasi, Teena; National Institute of Nutrition<br>B, Santosh Kumar; National Institute of Nutrition, Clinical Division<br>Kumar, Ramachandrappa Naveen; National Institute of Nutrition<br>Fahmida, Umi; Southeast Asian Ministry of Education Organisation Regional Centre for Food and Nutrition (SEAMEO RECFON)<br>Htet, Min Kyaw; Southeast Asian Ministry of Education Organisation Regional Centre for Food and Nutrition (SEAMEO RECFON)<br>Sudibya, Arienta R.P.; Southeast Asian Ministry of Education Organisation Regional Centre for Food and Nutrition (SEAMEO RECFON)<br>Faye, Babacar; Université Cheikh Anta Diop (UCAD)<br>Tine, Roger ; University of Cheikh Anta DIOP, Department of Parasitology-Mycology<br>Heffernan, Claire; University of London, Department of Pathobiology and Population Sciences; London International Development Centre<br>Saxena, Deepak ; Public Health Foundation of India<br>Dreibelbis, Robert; London School of Hygiene and Tropical Medicine, Department of Disease Control<br>Häsler, Barbara N; Royal Veterinary College (RVC), Department of Pathobiology and Population Sciences
Keywords:	Epidemiology, Microbiology, Data Collection, Growth

SCHOLARONE™
Manuscripts

I, the Submitting Author has the right to grant and does grant on behalf of all authors of the Work (as defined in the below author licence), an exclusive licence and/or a non-exclusive licence for contributions from authors who are: i) UK Crown employees; ii) where BMJ has agreed a CC-BY licence shall apply, and/or iii) in accordance with the terms applicable for US Federal Government officers or employees acting as part of their official duties; on a worldwide, perpetual, irrevocable, royalty-free basis to BMJ Publishing Group Ltd ("BMJ") its licensees and where the relevant Journal is co-owned by BMJ to the co-owners of the Journal, to publish the Work in this journal and any other BMJ products and to exploit all rights, as set out in our licence.

The Submitting Author accepts and understands that any supply made under these terms is made by BMJ to the Submitting Author unless you are acting as an employee on behalf of your employer or a postgraduate student of an affiliated institution which is paying any applicable article publishing charge ("APC") for Open Access articles. Where the Submitting Author wishes to make the Work available on an Open Access basis (and intends to pay the relevant APC), the terms of reuse of such Open Access shall be governed by a Creative Commons licence – details of these licences and which Creative Commons licence will apply to this Work are set out in our licence referred to above.

Other than as permitted in any relevant BMJ Author's Self Archiving Policies, I confirm this Work has not been accepted for publication elsewhere, is not being considered for publication elsewhere and does not duplicate material already published. I confirm all authors consent to publication of this Work and authorise the granting of this licence.

Confidential: For Review Only

Understanding the role of household hygiene practices and foodborne disease risks in child stunting: a UKRI GCRF Action Against Stunting Hub protocol paper

Paula Dominguez-Salas[ab], Hugh Sharma Waddington[c], Delia Grace[ab], Caroline Bosire[b], Arshnee Moodley[bd], Bharati Kulkarni[e], Teena Dasi[e], Santosh Kumar Banjara[e], Ramachandrappa Naveen Kumar[e], Umi Fahmida[f], Kyaw Htet[f], Arienta R.P. Sudibya[f], Babacar Faye[g], Roger C. Tine[g], Claire Heffernan[hi], Deepak Saxena[j], Robert Dreibelbis[c], Barbara Häsler[i]

[a]Natural Resources Institute (NRI), University of Greenwich, London, United Kingdom

[b]International Livestock Research Institute (ILRI), Nairobi, Kenya

[c]Environmental Health Group, Department of Disease Control, London School of Hygiene and Tropical Medicine, London, United Kingdom

[d]Department of Veterinary and Animal Sciences, University of Copenhagen, Copenhagen, Denmark

[e]National Institute of Nutrition (ICMR), Hyderabad, Telangana, India

[f]Southeast Asian Ministers of Education Organization Regional Center for Food and Nutrition (SEAMEO-RECFON)-Pusat Kajian Gizi Regional, Universitas Indonesia, Jakarta, Indonesia

[g]Department of Parasitology-Mycology, University of Cheikh Anta DIOP, Dakar, Senegal

[h]London International Development Centre (LIDC), London, United Kingdom

[i]Department of Pathobiology and Population Sciences, Royal Veterinary College (RVC), Hatfield, United Kingdom

[j]Indian Institute of Public Health Gandhinagar (IIPHG), India

Corresponding author:

Paula Dominguez Salas, Natural Resources Institute, University of Greenwich, Chatham Maritime, Kent, United Kingdom, P.DominguezSalas@gre.ac.uk

Keywords: food safety, hygiene, water, sanitation, child stunting, protocol

Confidential: For Review Only

ABSTRACT

Introduction: Environmental hygiene and food safety are important determinants of child stunting. This research aims to explore the relationship between child stunting and household hygiene practices and behaviours, including the availability of water, sanitation and hygiene (WASH) facilities; the use of safe food and good quality drinking water (especially when used for complementary feeding); hygienic practices in food transport, storage and preparation; and the control of cross-contamination from animals, their produce and waste.

Methods and analysis: This study is part of a wider observational study which aims to investigate the interdisciplinary factors contributing to child stunting using a 'whole child' paradigm. The observational study recruits women during pregnancy in Hyderabad, India, Lombok, Indonesia and Kaffrine, Senegal, and dyads (i.e., 500 mother-infant pairs per country) are followed longitudinally up to 24 months after birth. Within the interdisciplinary niche, the study herein has developed tools to investigate the potential exposure pathways to environmental pathogen contamination of foods and water. Holistic WASH and food safety data collection tools have been developed to explore exposure pathways at the household-level, including: i) survey questionnaires; ii) spot-checks; iii) biological sampling of drinking water, food and domestic surfaces; and iv) direct observation. An integrated analytical approach will be used to triangulate the evidence in order to examine the relationships between child stunting, WASH and food safety behaviours.

Ethics and dissemination: Ethical approval of the study was granted by the ethics committee of the LSHTM, RVC, ILRI, ICMR, IIPHG, SEAMEO-RECFON, University of Cheikh Anta Diop. Findings of the study will be disseminated through publication in peer-reviewed journals, relevant international conferences, public engagement events, and policy-maker and stakeholder events.

Confidential: For Review Only

SUMMARY BOX

- **What is already known on this topic**
  - From the moment complementary feeding begins, the chances of exposure to food contamination and infectious disease increase exponentially
  - Adequate WASH and food safety practices can play a key role in reducing infectious disease transmission and child malnutrition.
  - The contribution of hygiene behaviours and food contamination in driving transmission of infections and childhood stunting requires further investigation.

- **What this study adds**
  - Development, application and analysis of integrated WASH and food safety tools, towards a holistic understanding of how these practices and behaviours impact child stunting
  - Evidence towards a new typology of child stunting, with a focus on exposure pathways for pathogens, and to support programmes and policies to minimise stunting in childhood.

- **How this study might affect research, practice or policy**
  - Understanding of the role of home hygiene and food safety related practices in childhood stunting with a view to identify solutions.
  - New and more integrated approaches in research and practice that bring together WASH and food safety considerations at the household level to investigate exposure to pathogens.
  - Evidence on complementarities between food hygiene and WASH in order to help design suitable interventions to combat stunting.

Confidential: For Review Only

INTRODUCTION

The incidence and severity of stunting in childhood is closely linked to exposure to infection from food, drinking water and the wider environment.[1] Infections associated with contaminated water or insufficient water, sanitation and hygiene (WASH) are responsible for an estimated 21% of the total global burden of diseases,[2] contributing to the outbreak and chronicity of preventable infections such as diarrhoeal diseases and ARI (Acute Respiratory Infections), which are the two leading causes of death in children globally.[3] However, three recent randomised controlled trials (RCTs) have assessed the impacts of WASH combination interventions on nutrition in Bangladesh,[4] Kenya[5] and Zimbabwe.[6] The studies were not able to detect any effects on child linear growth, and only in Bangladesh was diarrhoea reduced.[7] Cumming, et al. have challenged the efficacy of the WASH interventions in addressing common causes of faeco-oral disease in low-income contexts, such as cryptosporidium.[8] More generally, the interventions did not increase the quantity of water supply available to practice domestic hygiene.

In addition, the World Health Organization (WHO) Foodborne Disease Burden Epidemiology Reference Group (FERG), estimated that approximately 40% of the global burden of foodborne diseases occurs in children under the age of five years.[9] A common cause of foodborne infections is the consumption of raw or undercooked meat, fish, seafood, eggs, fresh produce and dairy products contaminated by norovirus, Campylobacter, non-typhoidal Salmonella or pathogenic *E. coli.[9]* Food hygiene is a primary concern in the preparation of complementary foods - for example, bottles often cannot be adequately sterilised and perishable complementary foods are often left unrefrigerated or eaten using unclean utensils or with unwashed hands.

Disease transmission from faeces can be food- or water-borne (i.e., oral ingestion of contaminated food and water), water-washed or water-scarce (i.e., spread through inadequate hand and food hygiene) and water-based (i.e., transmitted by parasites that penetrate skin in water, such as schistosomiasis, or by walking barefoot on contaminated soil in the case of hookworm).[10] For example, theory and evidence suggests that pathogens in foods are a main driver of infant faecal-oral disease.[11] Field studies have demonstrated high bacterial contamination from *E. Coli* or Salmonella in weaning foods.[12, 13] Of particular importance for the cleanliness of infant complementary foods are the quality of water, surfaces and utensils used to prepare and eat them[14-16] as well as the quality of the food ingredients procured.

Hence, the availability of WASH and food storage and preparation facilities is likely to determine exposure to infection. Nonetheless, pathogen exposure can also arise when available facilities are used inadequately. For example, cultural norms and beliefs can restrict domestic food and hygiene practices – e.g., whether mothers are the principal decisionmakers about food preparation and hygiene in the household, or whether women are able to use the same toilet facilities as other household members.[17] However, while contamination of foods during cooking and feeding is common, the behavioural drivers have not been adequately explored. A recent systematic survey of 350 WASH evaluation projects in low and middle income countries (LMICs) found that around 15% of studies examined nutrition outcomes like stunting, and handwashing before food preparation, but only 5% reported on other food hygiene behaviours, such as whether food was stored appropriately and kitchen utensils were washed.[18]

Whilst food safety and WASH are inter-linked, to date little attention has been paid to understanding the relationships and trade-offs between food safety, WASH and child stunting. The safety of livestock and fish derived foods is a particularly neglected area and there has been no systematic integration of these topics in food safety and WASH protocols.[14] The UKRI GCRF Action Against Stunting Hub (AASH) explores child stunting from the Whole Child Approach, that is, considering different aspects of child development, including biology considerations, the home environment, the educational

environment, and the food system, that can contribute to thriving childhood. The aim of this study is to examine WASH and food safety elements, their convergence and contributions to child stunting at the household level. It evaluates WASH and food safety practices across households in three study sites with the objective to provide evidence on potential exposure to pathogens through contaminated environment, drinking water, and complementary food, and associated risk factors.

METHODS AND ANALYSIS

Conceptual Framework

The manner in which WASH and food safety at the household level can potentially lead to changes in infection and dietary intake, thereby affecting child stunting, is illustrated in Figure 1, adapted from the UNICEF´s (1990) conceptual framework on the causes of malnutrition.[19] Inadequate WASH practices and food safety can contribute to childhood stunting directly, by exposing infants to infection via complementary foods contaminated during preparation and storage, or through feeding with unclean utensils, including the hands of carers. The safety of foods can also be compromised before arrival at households, throughout the different steps and processes of the food value chain. Those food hazards can be intrinsic to the food produced (e.g., pathogens from animal diseases, heavy metal marine contamination), or extrinsic through processing and handling (e.g., inadequate hygienic practices, aflatoxin contamination during storage).[20] Mechanisms include nutrient loss (e.g., during diarrhoeal episodes), energy used by the immune system (e.g., in combating illnesses), and increased intestinal permeability and reduction in nutrients absorption (e.g., enteropathy).[21]

Figure 1: A conceptual framework showing the linkages of WASH and food safety with stunting at the household level

In theory, access to adequate WASH facilities enables appropriate drinking water, hygiene and food safety practices, contributing to reduction in transmission of food- and water-borne pathogens and improving nutrient uptake, and ultimately child growth and development.[20] Drinking water may be contaminated at the point of supply, or through transport and storage.[15, 16] The availability of clean water is critical for optimal hygiene practices[10], to address contamination by human and animal faeces,[22] and to prepare food hygienically. Sanitation facilities can enable safe faeces disposal, especially those of young children, which can be particularly pathogenic.[23] Faecal contamination can also come from the presence of domestic and wild animals or pests inside the household or in surrounding spaces.[24] It is expected that these sources of infection become more important as a child starts crawling and interacting with their environment. As such, understanding of the determinants of food and hygiene practises in the household is key to improving infant health and nutrition.[25]

Along with direct exposure, there is also an indirect route from water supply and sanitation facilities to childhood malnutrition, which operates through the opportunity costs of resource use. The time carers have to observe, correct, and address unhygienic behaviour, and the scarcity of time in relation to competing activities that need to be done by carers in the home or workplace, may also be detrimental for children's nutritional outcomes.[26] For example, it is typically women and older children, both of whom are more likely than others to be carers of young children, who spend time fetching water, engaging with animal fodder and collecting firewood. This may occur at the expense of adequate childcare and feeding, food preparation, education or rest.[27] When drinking water

Confidential: For Review Only

needs cannot be met affordably, households may need to purchase less food in order to afford enough water to drink. This kind of trade-off is clearly more prevalent for the poorest households, for whom malnutrition is also likely to be highest.

The WASH and food safety approaches, including their scope, evolution and methods are described in Table 1. Combining both WASH and food safety approaches provides an opportunity for more effective integration and learning of the two disciplines and their contributions to child growth and development. The two should converge when looking at household units with a central focus such as child stunting.

Table 1. Characteristics of Water, Sanitation and Hygiene (WASH) and Food Safety and Veterinary Public Health research

	**Water, Sanitation and Hygiene (WASH)**	**Food Safety and Veterinary Public Health**
Scope	To investigate deficiencies in water supply and quality, sanitation and hygiene to improve public health. Also, to evaluate the adequacy of hygiene standard to minimise food-borne diseases risks, and the role of water supply and sanitation access as enabling factors for improving child care, health and nutrition.	To pursue a comprehensive "farm to fork", "stable to table", "boat to throat" approach to prevent and minimise risks of food contamination at all stages of the food chain from production to human consumption and waste management.
Sector	Human-centred with a strong anchor in the public health sector.	Focus on animal source foods, zoonoses and their respective risks.
Evolution	Historically, WASH research has focused on water-related disease transmission from human excreta (faecal-oral diseases). This has contributed to understanding the mechanisms of faecal-oral infection through water-borne, water-washed, and water-based routes. Early intervention studies focused on the provision of infrastructure, and information, education and communication (IEC).<br><br>Current research focuses more on behaviour change communication using bottom-up approaches, and incorporating the "A (Animals)" into WASH more holistically.[24]	Veterinary public health traditionally looked at all food chain stages to identify where risks (e.g., biological, chemical, and physical) can emerge and how to prevent or mitigate them. The initial focus was on microbiological aspects with a strong technical dimension.<br><br>Veterinary public health now looks at human behaviour more widely, bringing in social science aspects and economics to understand better practices and the motivations behind them. There is a (slow) move towards systems thinking, and a lot of progress in quantitative microbiological and epidemiological studies, detection techniques, etc.

Confidential: For Review Only

Research methods	▪ Interventions (e.g., randomised controlled trials)<br>▪ Cross-sectional, questionnaire- based surveys<br>▪ Longitudinal questionnaire-based surveys<br>▪ Spot checks<br>▪ Direct observation<br>▪ Biological sampling and testing<br>▪ Risk assessments<br>▪ Qualitative research on behaviour, practices and perceptions, including participatory approaches

Study Design

This is a multicountry longitudinal observational study where women are recruited during pregnancy in Senegal, Indonesia and India and mother-infant pairs are followed up to 24 months after birth. Average stunting prevalences in these three countries selected are medium (17.9% in Senegal) to very high (30.8% in Indonesia and 37.4% in India).[28] This protocol involves multiple approaches to assess WASH and food safety practices in urban or rural households of women enrolled in the study, in Hyderabad city, India, East Lombok, Indonesia and Kaffrine, Senegal, at predefined timepoints (see AASH observational cohort description in Heffernan et al in this supplement, including further details on the study setting, sampling process, participant schedule, data collection and quality control, data management, etc).

Data Collection

This WASH and food safety data collection protocol focuses on three areas: exposure pathways to wider environmental pathogens, foodborne hazards, and waterborne hazards. Four data collection tools for the household-level have been developed, namely: i) a module for WASH and food safety to be included to the cohort questionnaire-based interviews (see Appendix 1); ii) spot-checks (see Appendix 2); iii) biological sampling; and iv) direct observation (see Appendix 3). The interviews and spot-checks take place simultaneously during home visits at 9, 12 and 18 months after birth. Data will be collected throughout the different seasons. Direct observation and biological sampling are conducted during a separate subsequent visit at each timepoint, in a subsample of the cohort. No additional inclusion or exclusion criteria were considered in the selection. Food and water samples are kept to a minimum for the analysis required, namely half filling a 50ml Falcon tube with food served to the child, and the measure of the container in which the child is normally given drinking water, respectively.

The food safety component of the data collection focuses on nutrient-rich foods with the potential to alleviate stunting, particularly on animal source foods (ASFs). This is due to their important nutritional profile,[29] their documented potential to alleviate stunting,[30-33] and their high food safety risk profile. Selected ASFs will be considered at each country, according to their relevance.

Trained enumerators collect data using electronic tablets in all study households, as summarised in Table 2. The direct observation tool is recorded on paper and enumerator positioning and previous rapport creation are used as ways to minimise bias. Privacy is maintained by stopping the direct observation on the respondent's request. Data collection tools developed in English are translated into local languages and backtranslated into English to check for accuracy, and piloted in the field.

Table 2. Summary of the integrated study methods and tools used to assess WASH and food safety at the household level

Confidential: For Review Only

Tool	Description	Sample size
Questionnaire-based interview	Mothers are asked about infant and young child feeding (IYCF) practices, household food expenditure, and the main household decision-makers and influencers of these activities. Physical activity questionnaires ask mothers about the times spent on work and housekeeping including collecting water, fodder and fuel. Furthermore, there are questions on water sources, water security, sanitation and handwashing. Other questions focus on the practices of acquisition, transport, storage, preparation of ASF, and the points of potential contamination in the household. Finally, other questions refer to the presence and behaviour of animals and potential transmission pathways associated with close proximity to animals, such as direct contact between animals and children or food.<br><br>The hazards and risks prior to arrival of ASFs in the household are elucidated in a separate component, at the value chain level (see Cooper et al in this supplement).	Whole cohort (500 households per country)
Spot-check	Enumerators observe behavioural issues alongside the interview questionnaire. The data are recorded using a check list on the availability and type of sanitation facilities available at the households (e.g., the place for defecation and proximity), handwashing infrastructure (e.g., location, availability of water and soap), presence of animals (e.g., livestock, pests), food storage facilities and practices (e.g., functioning refrigerator), and food preparation (e.g., easy to clean equipment).	Whole cohort (500 households per country)
Direct observation	Enumerators stay at the household for 3 hours around a feeding event, observing the mothers perform their daily activities and taking a passive approach to blend into the background in order to better observe hygiene related behaviours. Structured observation include activities related to water collection and storage, as well as personal, environmental and food hygiene (food preparation, storage, child feeding, handwashing, and animal contact). This data collection method is a gold standard for actual observation of behavioural practices.	Subsample (approximately one-third of full cohort in each country)
Biological sampling and testing	Enumerators collect samples of stored water, foods to be consumed by the children (as close to the feeding of the child as possible), and a swab of the main food preparation area at the households. Samples of hands rinse are optional.<br><br>The samples are stored in a coolbox and transported to a specialised laboratory for analysis. The samples are processed	Subsample (approximately one-third of full cohort in each country)

Confidential: For Review Only

	in-country and tested for overall contamination and for selected pathogens (such as Salmonella, Shigella, E.coli, and Campylobacter for food and E.coli/faecal indicators for surfaces and water), using standard laboratory protocols (of conventional culture and PCR). Part of the samples collected are used for lab analysis and the remaining part stored under -20°C for future analysis and crosschecking.	

ASF: animal source foods; IYCF: Infant and young child feeding; PCR: Polymerase chain reaction

Confidential: For Review Only

Data Management and Analysis

Data are collected at the households in CommCare and checked for accuracy before being transmitted to a central repository and can be downloaded in Excel for analysis. Samples are analysed at laboratories in each of the countries using tailored Standard Operating Procedures (SOPs).

Descriptive, comparative and regression analyses will be used to investigate the impact of WASH and food safety on child stunting. Factors relating to WASH and food safety amenities and practices will be considered simultaneously as external risks or preventive factors influencing the incidence and severity of stunting, using multivariable statistical approaches. To relate the main exposure variables (WASH and food safety factors) with the main outcome (stunting, measured as either z-scores or presence/absence), multivariable regressions models will be developed. According to our causal framework presented in Figure 1, we will adjust this model for potential confounding factors. Some of these confounding factors will be fairly stable (e.g. sex, maternal literacy, household facilities, socioeconomic status, etc.) and others will be time-varying (e.g. age, breastfeeding, environmental hygiene levels, etc.). We will avoid intermediate factors (e.g., infection rates). Subsequently, we will explore the potential role of the mediators in the causal framework using mediation analysis. Due to the existence of repeated measures conducted over three different timepoints for each child, we will account for this with a random effect term by individual in the regression model. The model will be a linear regression for stunting measured as a continuous variable, and a logistic (or ordered logistic) regression for stunting (moderate and severe) measured as a dichotomous variable. Due to the fact that in our 3 country settings the relevant association might differ, we will estimate the models separately in each country, and afterwards we will conduct individual participant data meta-analysis of the coefficients of interest across the countries. This strategy will allow for an optimal fit of the model in each country and at the same time we will be able to explore the heterogeneity of coefficients across countries and within sub-groups (by age, sex and socioeconomic characteristics).

Data triangulation will also be used, firstly, to address biases in individual data collection tools. For example, known biases occur when measures are reported by participants, particularly by those people for whom illness becomes normalised after repeated bouts of infection.[34] Observation of behaviours, through enumerator presence in the household at certain times of day, can help to mitigate reporting bias. However, surveys and observation may also affect participant motivation, particularly when repeated over long periods. Extensive collection of samples for laboratory testing can be done, but have their own weaknesses.[35] Secondly, data triangulation through statistical modelling will be used to determine the relationship between WASH and food safety in each of the study contexts, and to explore relevant questions such as whether they are complements or substitutes in child health and nutrition. As such, a mix of approaches is being used in this study to address these potential challenges.

Patient and Public Involvement Statement

Study participants have not been involved in the general design of the study. However, the study was designed in partnership with local research partners and stakeholders with experience in working at each of the settings. Local enumerators helped finalise the data collection tools during training and pilot testing.

Extensive consultations will be organised with the local communities to understand the ethos of the research including the involvement of participants and the implications of the research. Question and answer sessions will be held during the consultations to answer any questions and/or concerns raised. Study participants and the public will be involved in the dissemination of the study's findings through community discussion or engagement events.

DISCUSSION

Poor food safety and inadequate WASH, including getting in direct contact with animals, their produce and/or their faeces, are considered as important contributors to undernutrition. Cumulatively over the first two years of life, these factors can determine linear growth faltering. However, the causes of infant stunting are not exclusive to these factors, and therefore it is critical to cohesively consider the myriad of determinants involved. The UKRI GCRF AASH is using an interdisciplinary approach to investigate this variety of factors causing stunting with the aim to identify solutions for the amelioration of growth and development in early childhood at the 'whole-child' level. Within such an interdisciplinary framework, this study design will generate comprehensive evidence on the contribution of WASH, and food safety at the household level.

Challenges to conducting integrated assessment in WASH and food safety in a multi-country study like this are various, including identifying key ASFs and key hazards in a systematic way, or the analysis of pathways linking hygiene, food and water safety, and sanitation data into the typologies of stunting. For this purpose, we developed holistic data collection tools relevant to each study setting, to measure risk factors for exposure to food- and water-borne disease. Multiple household-level interventions are available in both WASH and food safety but, to date, their effectiveness has not been assessed together, despite the similarity in the exposure mechanisms they are targeting. The effectiveness of relevant interventions and technologies in addressing malnutrition depends on both their biological efficacy, and the degree to which consumers adapt to and use the new approach to their contexts.[8] We propose an approach and suggest data collection tools through which these various underlying and proximate determinants of child growth may be analysed.

The data generated from the study will help pave the way for future interventions to manage factors associated with and aimed at preventing stunting. At the individual level (immediate causes), inadequate practices and knowledge gaps identified will help tailor education and social behaviour change communication interventions. These are aimed at improving intake while reducing disease risks. At the household level (proximate causes) the findings can inform the implementation of more efficient WASH interventions, that ensure healthy environments and better personal hygiene. Ultimately, it can inform advocacy efforts to improve infrastructures and services at the community, regional or even national level (underlying causes).

ASF value chains generally present important potential to improve availability, accessibility, affordability and safety for the most nutritionally vulnerable population groups.[36] This study can provide information on mechanisms through which value chains are linked to stunting. The study also offers information that will help to identify entry points in chains where upgrading may be feasible and agreeable among stakeholders (e.g., consumers, government, and businesses). Based on secondary dietary and nutritional data, a systematic literature review of food safety hazards in ASF

Confidential: For Review Only

value chains in each study setting and local experts' feedback, commodities of likely relevance are eggs in all countries, milk in India and Senegal, and fish in Indonesia (see AASH food system protocol description in Cooper et al in this supplement for value chain work).

All in all, the described data, combined with data from other workstreams related to epigenetics, microbiome, gut health and nutrition, education, etc. also described in this supplement, this work aims to providing with a powerful piece of evidence for improving the child environment with a ´Whole child´ approach.

ETHICS AND DISSEMINATION

Ethical approval for the study was granted by the Institutional ethics committees of the London School of Hygiene and Tropical Medicine (17915/RR/17513), the Social Science Research Ethical Review Board at the Royal Veterinary College (URN SR2020-0198) and the International Livestock Research Institute Institutional Research Ethics Committee (ILRI-IREC2020-33). In-country approvals were also granted: the National Institute of Nutrition (ICMR), Ministry of Health and Family Welfare, Government of India (CR/04/I/2021), the Health Research Ethics Committee, University of Indonesia and Dr Cipto Mangunkusumo Hospital (KET-887/UN2.F1/ETIK/PPM.00.02/2019), and the Comité National d'Ethique pour la Recherche en Santé, Senegal (Protocole SEN19/78). Mothers and relevant family members are explained to all the procedures and processes prior to acceptance, then informed consent is obtained. All data and samples are anonymised with a unique identification number. Withdrawal from the study is allowed at any stage.

Findings of the study will be disseminated through publication in peer-reviewed journals, presented at relevant international conferences, public engagement events, and policy-maker and stakeholder events. Data generated from the study will be deposited in a publicly available data repository.

Confidential: For Review Only

AUTHORS CONTRIBUTIONS

All authors were involved in the study design and the preparation of the study tools and protocols at different stages; BH, HW and PDS drafted the manuscript sections; all other authors critically reviewed the manuscript. PDS and HW share first authorship.

We want to thank Alessia Gasco for producing the conceptual framework, David Prieto-Merino for his advice on statistics, and Kaitlin Conway and Modou Jobarteh for careful editing of the manuscript.

FUNDING STATEMENT

This work was supported by the UKRI-GCRF Action Against Stunting Hub (Project ref. MR/S01313X/1). The views expressed in this manuscript are those of the authors and do not necessarily represent the views of the funders.

COMPETING INTEREST STATEMENT

The authors declare no conflict of interest.

REFERENCES

[1] W. Checkley *et al.*, "Multi-country analysis of the effects of diarrhoea on childhood stunting," *International journal of epidemiology,* vol. 37, no. 4, pp. 816-830, 2008.

[2] R. E. Black *et al.*, "Global, regional, and national causes of child mortality in 2008: a systematic analysis," *The lancet,* vol. 375, no. 9730, pp. 1969-1987, 2010.

[3] L. Liu *et al.*, "Global, regional, and national causes of child mortality: an updated systematic analysis for 2010 with time trends since 2000," *The lancet,* vol. 379, no. 9832, pp. 2151-2161, 2012.

[4] S. P. Luby *et al.*, "Effects of water quality, sanitation, handwashing, and nutritional interventions on diarrhoea and child growth in rural Bangladesh: a cluster randomised controlled trial," *The Lancet Global Health,* vol. 6, no. 3, pp. e302-e315, 2018.

[5] C. Null *et al.*, "Effects of water quality, sanitation, handwashing, and nutritional interventions on diarrhoea and child growth in rural Kenya: a cluster-randomised controlled trial," *The Lancet Global Health,* vol. 6, no. 3, pp. e316-e329, 2018.

[6] J. H. Humphrey *et al.*, "Independent and combined effects of improved water, sanitation, and hygiene, and improved complementary feeding, on child stunting and anaemia in rural Zimbabwe: a cluster-randomised trial," *The Lancet Global Health,* vol. 7, no. 1, pp. e132-e147, 2019.

[7] A. J. Pickering *et al.*, "The WASH Benefits and SHINE trials: interpretation of WASH intervention effects on linear growth and diarrhoea," *The Lancet Global Health,* vol. 7, no. 8, pp. e1139-e1146, 2019.

[8] O. Cumming *et al.*, "The implications of three major new trials for the effect of water, sanitation and hygiene on childhood diarrhea and stunting: a consensus statement," *BMC medicine,* vol. 17, no. 1, pp. 1-9, 2019.

[9] A. H. Havelaar *et al.*, "World Health Organization Global Estimates and Regional Comparisons of the Burden of Foodborne Disease in 2010," *PLOS Medicine,* vol. 12, no. 12, p. e1001923, 2015, doi: 10.1371/journal.pmed.1001923.

Confidential: For Review Only

[10] G. F. White, D. J. Bradley, and A. U. White, "Drawers of water: domestic water use in East Africa," *Bulletin of the World Health Organization,* vol. 80, no. 1, pp. 63-73, 1972.

[11] Y. Motarjemi, F. Käferstein, G. Moy, and F. Quevedo, "Contaminated weaning food: a major risk factor for diarrhoea and associated malnutrition," *Bulletin of the World health Organization,* vol. 71, no. 1, p. 79, 1993.

[12] O. Touré, S. Coulibaly, A. Arby, F. Maiga, and S. Cairncross, "Piloting an intervention to improve microbiological food safety in Peri-Urban Mali," *International journal of hygiene and environmental health,* vol. 216, no. 2, pp. 138-145, 2013, doi: https://doi.org/10.1016/j.ijheh.2012.02.003.

[13] D. M. Kinkese, M. B. Hang'ombe, O. Toure, T. Kinkese, and E. Kangwa, "Contamination of complementary weaning foods for children with Escherichia coli and salmonella species in Lusaka District, Zambia.," *Journal of Preventive and Rehabilitative Medicine,* no. 1, pp. 19-31, doi: Doi:10.21617/jprm.2018.0101.3.

[14] O. P. Gautam, W.-P. Schmidt, S. Cairncross, S. Cavill, and V. Curtis, "Trial of a novel intervention to improve multiple food hygiene behaviors in Nepal," *The American journal of tropical medicine and hygiene,* vol. 96, no. 6, p. 1415, 2017.

[15] J. Wright, S. Gundry, and R. Conroy, "Household drinking water in developing countries: a systematic review of microbiological contamination between source and point-of-use," *Tropical medicine & international health,* vol. 9, no. 1, pp. 106-117, 2004.

[16] J. R. Lule *et al.*, "Effect of home based water chlorination and safe storage on diarrhea among persons with human immonodeficiency virus in Uganda," *American Journal of Tropical Medicine & Hygiene,* vol. 73, pp. 926–933, 2005.

[17] V. Curtis, "Hygiene: how myths, monsters, and mothers-in-law can promote behaviour change," *Journal of Infection,* vol. 43, no. 1, pp. 75-79, 2001.

[18] H. Chirgwin, S. Cairncross, D. Zehra, and H. Sharma Waddington, "Interventions promoting uptake of water, sanitation and hygiene (WASH) technologies in low-and middle-income countries: An evidence and gap map of effectiveness studies," *Campbell Systematic Reviews,* vol. 17, no. 4, p. e1194, 2021.

[19] Unicef, *Strategy for improved nutrition of children and women in developing countries. A UNICEF policy review.* New York: UNICEF, 1990.

[20] L. Unnevehr and D. Grace, *Aflatoxins: Finding solutions for improved food safety*. Intl Food Policy Res Inst, 2013.

[21] C. D. Bourke, J. A. Berkley, and A. J. Prendergast, "Immune dysfunction as a cause and consequence of malnutrition," *Trends in immunology,* vol. 37, no. 6, pp. 386-398, 2016.

[22] G. Penakalapati *et al.*, "Exposure to animal feces and human health: a systematic review and proposed research priorities," *Environmental science & technology,* vol. 51, no. 20, pp. 11537-11552, 2017.

[23] F. Majorin, B. Torondel, G. K. S. Chan, and T. Clasen, "Interventions to improve disposal of child faeces for preventing diarrhoea and soil-transmitted helminth infection," *Cochrane Database of Systematic Reviews,* no. 9, 2019.

[24] A. J. Prendergast *et al.*, "Putting the "A" into WaSH: a call for integrated management of water, animals, sanitation, and hygiene," *The Lancet Planetary Health,* vol. 3, no. 8, pp. e336-e337, 2019.

Confidential: For Review Only

[25] R. Aunger and V. Curtis, "Behaviour Centred Design: towards an applied science of behaviour change," *Health psychology review,* vol. 10, no. 4, pp. 425-446, 2016, doi: http://dx.doi.org/10.1080/17437199.2016.1219673.

[26] B. M. Popkin and F. S. Solon, "Income, time, the working mother and child nutriture," *Journal of tropical pediatrics,* vol. 22, no. 4, pp. 156-166, 1976.

[27] S. Cairncross and J. Cuff, "Water use and health in Mueda, Mozambique," *Transactions of the Royal Society of Tropical Medicine and Hygiene,* vol. 81, no. 1, pp. 51-54, 1987.

[28] M. Roser and H. Ritchie. Hunger and undernourishment [Online] Available: https://ourworldindata.org/hunger-and-undernourishment

[29] L. Iannotti *et al.*, "Livestock-derived foods and sustainable healthy diets," 2021.

[30] L. L. Iannotti *et al.*, "Eggs in early complementary feeding and child growth: a randomized controlled trial," *Pediatrics,* vol. 140, no. 1, 2017.

[31] D. Headey, K. Hirvonen, and J. Hoddinott, "Animal sourced foods and child stunting," ed: Wiley Online Library, 2018.

[32] H. De Beer, "Dairy products and physical stature: a systematic review and meta-analysis of controlled trials," *Economics & Human Biology,* vol. 10, no. 3, pp. 299-309, 2012.

[33] C. G. Neumann, S. P. Murphy, C. Gewa, M. Grillenberger, and N. O. Bwibo, "Meat supplementation improves growth, cognitive, and behavioral outcomes in Kenyan children," *the Journal of Nutrition,* vol. 137, no. 4, pp. 1119-1123, 2007.

[34] J. Briscoe, R. G. Feachem, and M. M. Rahaman, *Evaluating health impact: water supply, sanitation, and hygiene education*. IDRC, Ottawa, ON, CA, 1986.

[35] F. G. Goddard *et al.*, "Measuring environmental exposure to enteric pathogens in low-income settings: review and recommendations of an interdisciplinary working group," *Environmental science & technology,* vol. 54, no. 19, pp. 11673-11691, 2020, doi: DOI: 10.1021/acs.est.0c02421.

[36] P. Alarcon, P. Dominguez-Salas, E. M. Fèvre, and J. Rushton, "The importance of a food systems approach to low and middle income countries and emerging economies: a review of theories and its relevance for disease control and malnutrition," *Frontiers in Sustainable Food Systems,* p. 92, 2021, doi: https://doi.org/10.3389/fsufs.2021.642635.

Confidential: For Review Only

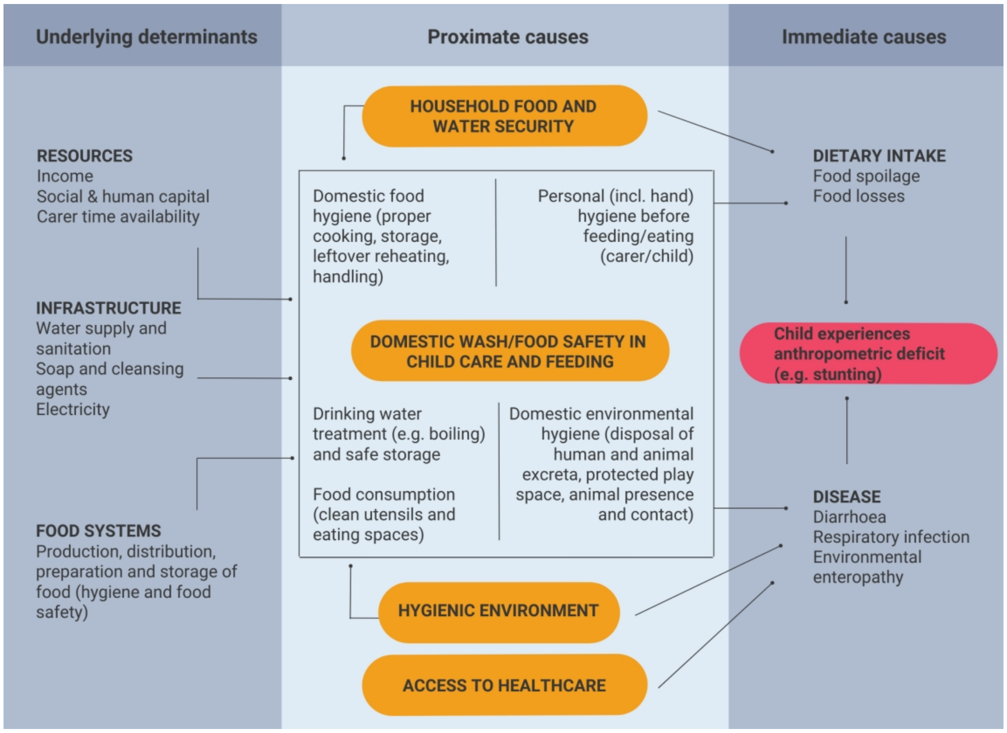

Figure 1: A conceptual framework showing the linkages of WASH and food safety with stunting at the household level

736x533mm (69 x 69 DPI)

APPENDIX 1: WASH AND FOOD SAFETY QUESTIONNAIRE

This questionnaire is administered by trained enumerators to mothers at 3 different time points, to elicit information about food safety and water and sanitation and hygiene practices at home, particularly in relation to infant and young child feeding. Data are collected in tablets on CommCare.

WATER SANITATION *(3 TIMES AT 3RD TRIMESTER, 3 & 24 MO)*
**ws_1. What is the main source of drinking water for members of your household?**
Piped waterpiped to dwelling
Piped waterpiped to compound yard or plot
Piped waterpiped to neighbor
Public tap / tapstand
Borehole or tubewell
Protected well
Unprotected well
Protected spring
Unprotected spring
Rainwater
Delivered watertanker or truck
Delivered watersmall cart with tank drum
Water kiosk
Packaged waterbottled water
Packaged watersachet water
Surface water
Other: specify
ws_1_96x. Other: specify
**ws_2. What is the main source of water used by members of this household for other purposes, such as cooking and handwashing?**
Piped waterpiped to dwelling
Piped waterpiped to compound yard or plot
Piped waterpiped to neighbor
Public tap / tapstand
Borehole or tubewell
Protected well
Unprotected well
Protected spring
Unprotected spring
Rainwater
Delivered watertanker or truck
Delivered watersmall cart with tank drum
Water kiosk
Packaged waterbottled water
Packaged watersachet water
Surface water
Other: specify
ws_2_96x. Other: specify
**ws_3. Where is this water collected from?**
In own dwelling

Confidential: For Review Only

In own yard
Elsewhere
**ws_4. How many minutes in total does it take you to go fetch water from this source and come back? (Fill in X minutes)**
**ws_5. Do you store drinking water in your home?**
Yes
No
**ws_6. Where do you store your drinking water?**
In a container with a lid/top on it
In an open container
Container on the roof
**ws_7. In the last 4 weeks/month, how frequently did you or anyone in your household worry you would not have enough water for all of your household needs?**
Never (0 times)
Rarely (1-2 times)
Sometimes (3-10 times)
Often (11-20times)
Always (more than 20 times)
**ws_8. In the last 4 weeks/month, how frequently has your main water source been interrupted or limited (eg water pressure, less water than expected, river dried up)?**
Never (0 times)
Rarely (1-2 times)
Sometimes (3-10 times)
Often (11-20times)
Always (more than 20 times)
**ws_9. In the last 4 weeks/month, how frequently has there not been enough water to wash clothes?**
Never (0 times)
Rarely (1-2 times)
Sometimes (3-10 times)
Often (11-20times)
Always (more than 20 times)
**ws_10. In the last 4 weeks/month, how frequently have you or anyone in your household had to change schedules or plans due to problems with your water situation? Activities that may have been interrupted include caring for others, doing household chores, agricultural work, income-generating activities, sleeping, etc**
Never (0 times)
Rarely (1-2 times)
Sometimes (3-10 times)
Often (11-20times)
Always (more than 20 times)
**ws_11. In the last 4 weeks/month, how frequently have you or anyone in your household had to change what was being eaten because there were problems with water (eg, for washing foods, cooking, etc)?**
Never (0 times)
Rarely (1-2 times)
Sometimes (3-10 times)
Often (11-20times)
Always (more than 20 times)

Confidential: For Review Only

**ws_12. In the last 4 weeks/month, how frequently have you or anyone in your household had to go without washing hands after dirty activities (eg, defecating or changing diapers, cleaning animal dung) because of problems with water?**
Never (0 times)
Rarely (1-2 times)
Sometimes (3-10 times)
Often (11-20times)
Always (more than 20 times)
**ws_13. In the last 4 weeks/month, how frequently have you or anyone in your household had to go without washing their body because of problems with water (eg, not enough water, dirty, unsafe)?**
Never (0 times)
Rarely (1-2 times)
Sometimes (3-10 times)
Often (11-20times)
Always (more than 20 times)
**ws_14. In the last 4 weeks/month, how frequently has there not been as much water to drink as you would like for you or anyone in your household?**
Never (0 times)
Rarely (1-2 times)
Sometimes (3-10 times)
Often (11-20times)
Always (more than 20 times)
**ws_15. In the last 4 weeks/month, how frequently did you or anyone in your household feel angry about your water situation?**
Never (0 times)
Rarely (1-2 times)
Sometimes (3-10 times)
Often (11-20times)
Always (more than 20 times)
**ws_16. In the last 4 weeks/month, how frequently have you or anyone in your household gone to sleep thirsty because there wasn't any water to drink?**
Never (0 times)
Rarely (1-2 times)
Sometimes (3-10 times)
Often (11-20times)
Always (more than 20 times)
**ws_17. In the last 4 weeks/month, how frequently has there been no useable or drinkable water whatsoever in your household?**
Never (0 times)
Rarely (1-2 times)
Sometimes (3-10 times)
Often (11-20times)
Always (more than 20 times)
**ws_18. In the last 4 weeks/month, how frequently have problems with water caused you or anyone in your household to feel ashamed/excluded/stigmatized?**
Never (0 times)
Rarely (1-2 times)
Sometimes (3-10 times)
Often (11-20times)

Confidential: For Review Only

Always (more than 20 times)
**ws_19. Is there a latrine or toilet that members of this household use regularly?**
Yes
No
**ws_20. Where is this toilet facility located?**
In own dwelling
In own yard or plot
Elsewhere
**ws_21. How many households other than your own share this facility?**
Enter the enumerator Name
**fh_1. Is there a designated place for handwashing in the household?**
Yes
No
**fh_2. How often is soap available at this location?**
Always
Sometimes
Never
**fh_3. What were all of the times when you washed your hands (Circle all that apply but do not prompt After each response, ask "any other times?")**
After defecating
After cleaning a child/wiping a child's bottom
After changing a babies nappy
Before preparing food
Before eating
Before feeding the child
After touching animals
After touching animal faeces
After handling raw animal source foods
After crop farming activities
Other
Cannot remember/does not know
fh_3_96x. Specify other
**FOOD STORAGE**
**fh_4. Do you store meat and meat products (except dried meat)?**
Yes
No
**fh_4a. Where do you store raw meat (fresh/chilled/frozen)?**
In a fridge
In a freezer
Covered in a cool room (below 5 degrees Celsius)
Uncovered in a cool room (below 5 degrees Celsius)
Covered in a room at room temperature
Uncovered in a room at room temperature
Other, specify
fh_4a_96x. Other, specify
**fh_4b. How long do you store the meat and meat products?**
less than 2 hours

Confidential: For Review Only

2 hours to 5 hours
5 hours to 12 hours
12 hours
1-3 days
3-7 days
1 week
1 month
**fh_4c. Where do you store processed meat (ready-to-cook and ready-to-eat)?**
In a fridge
In a freezer
Covered in a cool room (below 5 degrees Celsius)
Uncovered in a cool room (below 5 degrees Celsius)
Covered in a room at room temperature
Uncovered in a room at room temperature
Other, specify
fh_4c_96x. Other, specify
**fh_4d. How long do you store the meat and meat products?**
less than 2 hours
2 hours to 5 hours
5 hours to 12 hours
12 hours
1-3 days
3-7 days
1 week
1 month
**fh_5. Do you store milk and milk products (except milk powder, evaporated milk or condensed milk)?**
Yes
No
**fh_5a. Where do you store liquid milk (including pasteurized and non-pasteurized)?**
In a fridge
In a freezer
Covered in a cool room (below 5 degrees Celsius)
Uncovered in a cool room (below 5 degrees Celsius)
Covered in a room at room temperature
Uncovered in a room at room temperature
Other, specify
fh_5a_96x. Other, specify
**fh_5b. Where do you store solid/semi solid dairy/milk products (e.g cheese, yoghurt, ghee, etc)?**
In a fridge
In a freezer
Covered in a cool room (below 5 degrees Celsius)
Uncovered in a cool room (below 5 degrees Celsius)
Covered in a room at room temperature
Uncovered in a room at room temperature
Other, specify
fh_5b_96x. Other, specify
**fh_6. Do you store raw or cooked vegetables and fruits?**

Confidential: For Review Only

Yes
No
**fh_6a. Where do you store raw vegetables?**
In a fridge
In a freezer
Covered in a cool room (below 5 degrees Celsius)
Uncovered in a cool room (below 5 degrees Celsius)
Covered in a room at room temperature
Uncovered in a room at room temperature
Other, specify
fh_6a_96x. Other, specify
**fh_6b. Where do you store cooked vegetables and fruit?**
In a fridge
In a freezer
Covered in a cool room (below 5 degrees Celsius)
Uncovered in a cool room (below 5 degrees Celsius)
Covered in a room at room temperature
Uncovered in a room at room temperature
Other, specify
fh_6b_96x. Other, specify
**fh_7. Do you store food that has been prepared for your child to give it to the child later?**
Yes
No
**fh_7a. How do you store food that has been prepared for your child?**
Thermos
Jug
Plate or bowl (covered)
Plate or bowl (uncovered)
Cooking pot (covered)
Cooking pot (uncovered)
Other container (covered)
Other container (uncovered)
Other, specify
fh_7a_96x. Other, specify
**fh_7b. Where do you store this food?**
In a fridge
In a freezer
In a cool room (below 5 degrees Celsius)
In a room at room temperature
Other, specify
fh_7b_96x. Other, specify
**How long do you store this food?**
less than 1 hour
1-2 hours
2 hours to 5 hours
5 hours to 12 hours
12 hours

Confidential: For Review Only

1-3 days
3-7 days
Longer than a week
Animals
**fh_8. Does your household keep any animals inside or outside the house you are looking after (i.e. not taken care of by others)?**
Yes
No
**fh_9. What type of animals?**
Chickens
Ducks
Other birds
Dogs
Cats
Goats
Sheep
Pigs
Cows
Fish
Other animals, specify
fh_9_96x. Other: specify
**fh_10. Which of these animals are in the patio, veranda or house any time of the day?**
None
Chickens
Ducks
Other birds
Dogs
Cats
Goats
Sheeps
Pigs
Cows
Other animals, specify
fh_10_96x. Other: specify
**fh_10a. Is the animal contained in some form of enclosure?**
Yes
No
**fh_10b. Can the child be in contact with the animals or their faeces?**
Yes
No
**fh_10c. Can the animal be in contact with food for the child?**
Yes
No
**ASF1_1. What is the first priority ASF consumed by the child most recently? Select [ASF1]**
Chicken
fish
beef
egg

Confidential: For Review Only

squid
meat ball
beef soup
chicken soup
duck egg
fish egg
None
**ASF1_2. How was this [ASF] packaged when you got it from the source? (select all that apply)**
No packaging
Sealed package
Washable container (plastic, glass, steel), open
Washable container (plastic, glass, steel), closed
Plastic bag
Newspaper
Other paper
Aluminium foil
Other foil
Container made from natural materials
Other, specify
ASF1_2_96x. ASF_2_96x Other: specify
**ASF1_3. Did you transport this [ASF] after you got it?**<br>**No transport means that they either produced it themselves or the ASF was brought to them (e.g. by a trader or neighbour, etc)**
Yes
No
**ASF1_3a. How long did you transport the ASF for (to take it home)?**
less than 5 minutes
5 min to 15 min
15 to 30 min
30 min to 1 hour
1hour to 2 hours
2 hours to 3 hours
3 hours
**ASF1_3b. How did you transport this [ASF] after you got it?**
Chilled (e.g. on ice)
At ambient temperature
Kept warm (e.g. thermos or thermal food container)
Other, specify
ASF1_3b_96x. Other, specify
**ASF1_4. Did you store this [ASF] before preparation?**
Yes
No
**ASF1_4a. Was this [ASF] covered when you stored it before preparation?**
Yes
No
**ASF1_4b. Where did you store this [ASF] before preparation?**
In a fridge
In a freezer

Confidential: For Review Only

In a cool room (below 5 degrees Celsius)
In a room at room temperature
Other, specify
ASF1_4b_96x. Specify, other
**ASF1_4c. How long was this [ASF] stored before preparing?**
minute
hour
day
total minutes
total hours
total day
**ASF1_5. Did you wash this [ASF] before preparation?**
Yes
No
Not applicable
**ASF1_6. If yes, why did you wash this [ASF]? (multiple answers)**
To remove visible dirt
To remove visible blood
To make it safer
Because it is a good practice
Other, specify
ASF1_6_96x. Specify, other
**ASF1_7. Did you chop or cut this [ASF] product before preparation?**
Yes
No
Not applicable
**ASF1_8. If yes, which tool(s) did you use to chop or cut this [ASF]? (multiple answers)**
Cutting board
Plate
Knife
Other, specify
ASF1_8_96x. Specify, other
**ASF1_9. How did you prepare this food for the child?**
Grilling
Baking
Frying
Boiling
Stewing
Simmering
Fermenting
Heating
Mixed in with other ingredients without cooking the ASF (e.g. mixing yoghurt with fruit or adding dried fish to salad)
Made smaller portion (e.g. cutting in pieces or butting in a bowl), but did not cook or mix with other ingredients
Other, specify
ASF1_9_96x. Specify, other
**ASF1_10. For how long was this [ASF] cooked?**

Confidential: For Review Only

1-5 min
5-15 min
15-30 min
30-60 min
More than 1 hour
**ASF1_11. After preparation, was this [ASF] stored before giving it to the child?**
Yes
No
**ASF1_12. After preparation, how long was this [ASF] stored before giving it to the child?**
1-5 min
5-15 min
15-30 min
30-60 min
More than 1 hour, less than 24h
More than 1 day, less than 2 days
More than 2 days
**ASF1_13. How did you store this [ASF] that had been prepared for your child?**
Thermos
Jug
Plate or bowl (covered)
Plate or bowl (uncovered)
Cooking pot (covered)
Cooking pot (uncovered)
Other container (covered)
Other container (uncovered)
Other, specify
ASF1_13_96x. Specify, other
**ASF1_14. Where did you store this [ASF]?**
In a fridge
In a freezer
In a cool room (below 5 degrees Celsius)
In a room at room temperature
Other, specify
ASF1_14_96x. Specify other
**ASF2_1. What is the second priority ASF consumed by the child most recently? Select [ASF1]**
Chicken
fish
beef
egg
squid
meat ball
beef soup
chicken soup
duck egg
fish egg
None
**ASF2_2. How was this [ASF] packaged when you got it from the source? (select all that apply)**

Confidential: For Review Only

No packaging
Sealed package
Washable container (plastic, glass, steel), open
Washable container (plastic, glass, steel), closed
Plastic bag
Newspaper
Other paper
Aluminium foil
Other foil
Container made from natural materials
Other, specify
ASF_2_96x Other: specify
**ASF2_3. Did you transport this [ASF] after you got it?** **No transport means that they either produced it themselves or the ASF was brought to them (e.g. by a trader or neighbour, etc)**
Yes
No
**ASF2_3a. How long did you transport the ASF for (to take it home)?**
less than 5 minutes
5 min to 15 min
15 to 30 min
30 min to 1 hour
1hour to 2 hours
2 hours to 3 hours
3 hours
**ASF2_3b. How did you transport this [ASF] after you got it?**
Chilled (e.g. on ice)
At ambient temperature
Kept warm (e.g. thermos or thermal food container)
Other, specify
ASF2_3b_96x. Other specify
**ASF2_4. Did you store this [ASF] before preparation?**
Yes
No
**ASF2_4a. Was this [ASF] covered when you stored it before preparation?**
Yes
No
**ASF2_4b. Where did you store this [ASF] before preparation?**
In a fridge
In a freezer
In a cool room (below 5 degrees Celsius)
In a room at room temperature
Other, specify
ASF2_4b_96x. Specify, other
**ASF2_4c. How long was this [ASF] stored before preparing?**
minute
hour
day

Confidential: For Review Only

total minutes
total hours
total day
**ASF2_5. Did you wash this [ASF] before preparation?**
Yes
No
Not applicable
**ASF2_6. If yes, why did you wash this [ASF]? (multiple answers)**
To remove visible dirt
To remove visible blood
To make it safer
Because it is a good practice
Other, specify
ASF2_6_96x. Specify, other
**ASF2_7. Did you chop or cut this [ASF] product before preparation?**
Yes
No
Not applicable
**ASF2_8. If yes, which tool(s) did you use to chop or cut this [ASF]? (multiple answers)**
Cutting board
Plate
Knife
Other, specify
ASF2_8_96x. Specify, other
**ASF2_9. How did you prepare this food for the child?**
Grilling
Baking
Frying
Boiling
Stewing
Simmering
Fermenting
Heating
Mixed in with other ingredients without cooking the ASF (e.g. mixing yoghurt with fruit or adding dried fish to salad)
Made smaller portion (e.g. cutting in pieces or butting in a bowl), but did not cook or mix with other ingredients
Other, specify
ASF2_9_96x. Specify, other
**ASF2_10. For how long was this [ASF] cooked?**
1-5 min
5-15 min
15-30 min
30-60 min
More than 1 hour
**ASF2_11. After preparation, was this [ASF] stored before giving it to the child?**
Yes
No

Confidential: For Review Only

**ASF2_12. After preparation, how long was this [ASF] stored before giving it to the child?**
1-5 min
5-15 min
15-30 min
30-60 min
More than 1 hour, less than 24h
More than 1 day, less than 2 days
More than 2 days
**ASF2_13. How did you store this [ASF] that had been prepared for your child?**
Thermos
Jug
Plate or bowl (covered)
Plate or bowl (uncovered)
Cooking pot (covered)
Cooking pot (uncovered)
Other container (covered)
Other container (uncovered)
Other, specify
ASF2_13_96x. Specify, other
**ASF2_14. Where did you store this [ASF]?**
In a fridge
In a freezer
In a cool room (below 5 degrees Celsius)
In a room at room temperature
Other, specify
ASF2_14_96x. Specify, other
**ASF3_1. What is the third priority ASF consumed by the child most recently? Select [ASF1]**
Chicken
fish
beef
egg
squid
meat ball
beef soup
chicken soup
duck egg
fish egg
None
**ASF3_2. How was this [ASF] packaged when you got it from the source? (select all that apply)**
No packaging
Sealed package
Washable container (plastic, glass, steel), open
Washable container (plastic, glass, steel), closed
Plastic bag
Newspaper
Other paper
Aluminium foil

Confidential: For Review Only

Other foil
Container made from natural materials
Other, specify
ASF_2_96x Other: specify
**ASF3_3. Did you transport this [ASF] after you got it?<br>No transport means that they either produced it themselves or the ASF was brought to them (e.g. by a trader or neighbour, etc)**
Yes
No
**ASF3_3a. How long did you transport the ASF for (to take it home)?**
less than 5 minutes
5 min to 15 min
15 to 30 min
30 min to 1 hour
1hour to 2 hours
2 hours to 3 hours
3 hours
**ASF3_3b. How did you transport this [ASF] after you got it?**
Chilled (e.g. on ice)
At ambient temperature
Kept warm (e.g. thermos or thermal food container)
Other, specify
ASF3_3b_96x. Other, specify
**ASF3_4. Did you store this [ASF] before preparation?**
Yes
No
**ASF3_4a. Was this [ASF] covered when you stored it before preparation?**
Yes
No
**ASF3_4b. Where did you store this [ASF] before preparation?**
In a fridge
In a freezer
In a cool room (below 5 degrees Celsius)
In a room at room temperature
Other, specify
ASF3_4b_96x. Specify, other
**ASF3_4c. How long was this [ASF] stored before preparing?**
minutes
hour
day
total minutes
total hours
total day
**ASF3_5. Did you wash this [ASF] before preparation?**
Yes
No
Not applicable
**ASF3_6. If yes, why did you wash this [ASF]? (multiple answers)**

Confidential: For Review Only

To remove visible dirt
To remove visible blood
To make it safer
Because it is a good practice
Other, specify
ASF3_6_96x. Specify, other
**ASF3_7. Did you chop or cut this [ASF] product before preparation?**
Yes
No
Not applicable
**ASF3_8. If yes, which tool(s) did you use to chop or cut this [ASF]? (multiple answers)**
Cutting board
Plate
Knife
Other, specify
ASF3_8_96x. Specify, other
**ASF3_9. How did you prepare this food for the child?**
Grilling
Baking
Frying
Boiling
Stewing
Simmering
Fermenting
Heating
Mixed in with other ingredients without cooking the ASF (e.g. mixing yoghurt with fruit or adding dried fish to salad)
Made smaller portion (e.g. cutting in pieces or butting in a bowl), but did not cook or mix with other ingredients
Other, specify
ASF3_9_96x. Specify, other
**ASF3_10. For how long was this [ASF] cooked?**
1-5 min
5-15 min
15-30 min
30-60 min
More than 1 hour
**ASF3_11. After preparation, was this [ASF] stored before giving it to the child?**
Yes
No
**ASF3_12. After preparation, how long was this [ASF] stored before giving it to the child?**
1-5 min
5-15 min
15-30 min
30-60 min
More than 1 hour, less than 24h
More than 1 day, less than 2 days
More than 2 days

Confidential: For Review Only

**ASF3_13. How did you store this [ASF] that had been prepared for your child?**
Thermos
Jug
Plate or bowl (covered)
Plate or bowl (uncovered)
Cooking pot (covered)
Cooking pot (uncovered)
Other container (covered)
Other container (uncovered)
Other, specify
ASF3_13_96x. Specify, other
**ASF3_14. Where did you store this [ASF]?**
In a fridge
In a freezer
In a cool room (below 5 degrees Celsius)
In a room at room temperature
Other, specify
ASF3_14_96x. Other, specify

Confidential: For Review Only

APPENDIX 2: WASH AND FOOD SAFETY SPOT CHECK

This checklist is filled by trained enumerators in the households at 3 different time points, to observe and record information about behaviours and facilities in relation to food safety and water and sanitation and hygiene practices at home. Data are collected in tablets on CommCare.

WATER SANITATION (SPOT CHECK) *(3 TIMES AT 2ND TRIMESTER, 3 & 9MO)*
**wash_1. What type of facility is the primary location for defecation?**
Flush toilet to piped sewer system
Flush toilet to septic tank
Flush toilet to pit
Flush toilet to onsite, above ground, open pit
Pour flush toilet to piped sewer system
Pour flush toilet to septic tank
Pour flush toilet to pit
Pour flush toilet to onsite, above ground, open pit
Pit latrine with concrete slab (not pour flush)
Pit latrine without slab (not pour flush)
Bucket
Bag
Open defecation (no facilities)
Other:
Refused observation
wash_1_96x. Specify, other
**wash_2. Approximately how many meters is the toilet from the entrance of the household**
**wash_3. For the following, please note if following indicators of functionality are present**
**wash_3_1. Is the pan working (not broken)**
yes
no
**wash_3_2. Are there leaves or rubbish blocking the pan?**
yes
no
**wash_3_3. Is there standing water in the pan?**
yes
no
**wash_3_4. Is there a cover over the pit?**
yes
no
**wash_3_5. Are there one or two pits?**
One
Two
More
**wash_3_6. Is there a cover on all the pits?**
No
Partial (only to one pit)
Yes, to more than one pit
**wash_3_7. Are there flies?**

Confidential: For Review Only

None
Some
Many
**wash_4. Can you please show me where members of this household most often wash their hands?**
Fixed facility (sink/basin)… in dwelling
Fixed facility….in yard
Mobile object (bucket / jug)
No handwashing place in dwelling
Refused observation
**wash_5. Is there water available at this location?**
Yes
No
**wash_6. Is soap available at this location? (Soap includes bar soap, liquid soap, powder detergent, and soapy water but does not include ash, soil, sand or other handwashing agents)**
Yes
No
**wash_7. Is the dedicated location located near (within 5 steps) the primary cooking location?**
Yes
No
**wash_8. Is the dedicated location located near (within 5 steps) the latrine/toilet**
Yes
No
**wash_9. Are there animals located in or around the household? (select all)**
No animals visible
In the cooking area (outside house)
In the cooking area (inside house)
In the food storing area (outside house)
In the food storing area (inside house)
In other areas of the house that are not cooking or food storing areas
**wash_10. What type of animals (select all)**
Chickens
Ducks
Other birds
Dog
Cat
Goat
Sheep
Pig
Cow
Wild animals
Rodents
Other animal
wash_10_96x. Other, specify
**wash_11. Are there animal feces located in or around the household? (select all)**
No animal feces visible
In the cooking area (outside house)
In the cooking area (inside house)
In the food storing area (outside house)

Confidential: For Review Only

In the food storing area (inside house)
In other areas of the house that are not cooking or food storing areas
In the yard / courtyard
FOOD SAFETY QUESTIONNAIRE *(1 TIME: AT 6 MO)*
HAND WASHING
**spot_1. Can you please show me where members of this household most often wash their hands after using the toilets?**
Fixed facility (sink/basin)… in dwelling
Fixed facility….in yard
Mobile object (bucket / jug)
No handwashing place in dwelling
Refused observation
**spot_2. Is there water available at this location?**
Yes
No
**spot_3. Is soap available at this location? (Soap includes bar soap, liquid soap, powder detergent, and soapy water but does not include ash, soil, sand or other handwashing agents)**
Yes
No
**spot_4. Is the dedicated location located near (within 5 steps) the primary cooking location?**
Yes
No
**spot_5. Is the dedicated location located near (within 5 steps) the latrine/toilet**
Yes
No
**spot_6. Can you show me where members of this household most often wash their hands before eating or preparing food?**
Fixed facility (sink/basin)… in dwelling
Fixed facility….in yard
Mobile object (bucket / jug)
No handwashing place in dwelling
Refused observation
Same as after toilet
**spot_7. Is there water available at this location?**
Yes
No
**spot_8. Is soap available at this location? (Soap includes bar soap, liquid soap, powder detergent, and soapy water but does not include ash, soil, sand or other handwashing agents)**
Yes
No
**spot_9. Is the dedicated location located near (within 5 steps) the primary cooking location?**
Yes
No
**spot_10. Is the dedicated location located near (within 5 steps) the latrine/toilet**
Yes
No
**spot_11. Do you see animals in or around the household? (select all)**

Confidential: For Review Only

No animals visible
In the cooking area (outside house)
In the cooking area (inside house)
In the food storing area (outside house)
In the food storing area (inside house)
In other areas of the house that are not cooking or food storing areas
**spot_12. What type of animals (select all)**
Chickens
Ducks
Other birds
Dog
Cat
Goat
Sheep
Pig
Cow
Wild animals
Rodents
Other animal
spot_12_96x. Specify other
**spot_13. Is it possible for the child to be in contact with these animals (e.g. if they are unrestrained or the child can access an enclosure)?**
Yes
No
**spot_14. Is it possible for these animals to be in contact with food (e.g. if animals are unrestrained)**
Yes
No
**spot_15. Are there visible animal feces located in or around the household? (select all)**
No animal feces visible
In the cooking area (outside house)
In the cooking are (inside house)
In the food storing area (outside house)
In the food storing area (inside house)
In other areas of the house that are not cooking or food storing areas
In the yard / courtyard
In the animal enclosure (e.g. chicken coop)
**spot_16. Is it possible for the child to be in contact with these faeces (e.g. because they are in areas where the child is or the child can access an enclosure)?**
Yes
No
**spot_17. Is it possible that food gets in contact with these faeces?**
Yes
No
**safe_1. Is there a functioning refrigerator?**
Yes
No
**safe_2. Is there a functioning freezer?**
Yes

Confidential: For Review Only

No
**safe_3. Is there any visible, stored [ASF] not protected from the environment?**
Yes
No
**safe_4. Is it [ASF] in contact with other food products?**
Yes
No
**safe_5. Is the area where this [ASF] is kept protected from animals and flies?**
Yes
No
**safe_6. Is there a kitchen area (a dedicated food preparation area)?**
Yes
No
**safe_6a. Is the kitchen area free from pest animals (i.e. birds, flies, rats)**
Yes
No
**safe_6b. Are there anti-pest measures in place in the kitchen area? e.g. nets, traps, insect killers**
Yes
No
**safe_6c. Is the kitchen area free of visible dirt?**
Yes
No
**safe_6d. Are cooking utensils clean?**
Yes
No
**safe_6e. Is the kitchen area easy to clean (e.g. tiles)?**
Yes
No
**safe_6f. Does the kitchen area have access to running water?**
Yes
No
**safe_6g. If there is no access to running water, is there stored water in the area where food is being prepared?**
Yes, there is water in a container with a lid/top on it
Yes, there is water in an open container
No, there is no water
**safe_6h. Is soap available at this location? (Soap includes bar soap, liquid soap, powder detergent, and soapy water but does not include ash, soil, sand or other handwashing agents)**
Yes
No
**safe_7. Is there a food storage area?**
Yes
No
**safe_7a. In the food storage area, is the storage equipment made of material that is easy to clean (e.g. metal, plastic)?**
Yes
No
**safe_7b. Is the food storage area free from pest animals (i.e. birds, flies, rats)**

Confidential: For Review Only

Yes
No
**safe_7c. Are there anti-pest measures in place in the food storage area? e.g. nets, traps, insect killers**
Yes
No
**safe_7d. Is the food storage area free of visible dirt?**
Yes
No
**safe_7e. Is the food storage area easy to clean (e.g. tiles)?**
Yes
No
**safe_8. Is there a facility to dispose of food waste?**
No
In the cooking area (outside house)
In the cooking area (inside house)
In the food storing area (outside house)
In the food storing area (inside house)
Outside the house remove from cooking or food storing areas
Removed from the household
**safe_9. Are the knife and chopping board used to cut vegetables and fruits are clean?**
Yes
No
**safe_10.Are the nails of the food handler trimmed?**
Yes
No

Confidential: For Review Only

APPENDIX 3: WASH AND FOOD SAFETY DIRECT OBSERVATION TOOL

This direct observation tool is filled by trained enumerators in the households at 3 different time points, to observe and record information about behaviours related to food safety and water and sanitation and hygiene practices at home, particularly in relation to infant and young child feeding. Food, drinking water and surface samples are subsequently collected for microbiological testing. Data are collected on paper.

GCRF Action against stunting Hub

Confidential

**Household ID:**	**Mother ID:**	**Child ID:**
**Enumerator ID:**	**Supervisor ID:**	
**District:**	**Sub-county:**	
**Parish:**	**Village:**	
**Salter calibration ID:**	**Salter calibration weight:**	
**Date of record:**<br><br>**Time ARRIVED:** *(24-hr)*<br><br>**Time DEPARTED:** *(24-hr)*	**Day of the week <u>today</u>:**<br><br>Monday ☐ Friday ☐<br>Tuesday ☐ Saturday ☐<br>Wednesday ☐ Sunday ☐<br>Thursday ☐	

Complete at the end of the day

Describe the location of the observations: **Where in the home the observation was completed – where the observer sat, what the conditions were when you did the observation (weather, lighting, heat) how busy the home was, etc.**

Describe who the main caretaker of the child was:

Mother ☐
Sibling ☐
Grandmother ☐
Aunt ☐
Caretaker ☐
Other, specify ☐ ____

Please note if caretaker changes during observation: Yes ☐ **No** ☐ **Mother** ☐
Sibling ☐
Grandmother ☐
Aunt ☐
Caretaker ☐
Other, specify ☐ ____

Direct observation

Household ID: ______

	Yes *(tick)*	**Date:** *dd/mm/yy*
**Checked by supervisor:** *(code)*		
**Checked by co-ordinator:** *(code)*		
**Data entry completed:** *(code)*		

Activity	___ ____:00				____ ____:00				___ ____:00				____ ____:00				Pilot notes
	a	b	c	d	e	f	g	h	I	j	k	l	m	n	o	p	
	00	15	30	45	00	15	30	45	00	15	30	45	00	15	30	45	
1. Child Activities																	
1.1 Sleeping																	
1.2 Caretaker change																	
1.3 Child being cared for by other children																	
2. Child feces																	
2.1 Child nappy or diaper is changed																	
2.2 Child defecates on floor																	
2.3 Child defecates in potty																	
2.4 Child feces disposed in toilet																	
2.5 Child feces disposed in bush / ditch																	
3. Food preparation																	
3.1 Fruit or vegetable washed																	
3.2 Food preparation surface rinsed (specify surface type)																	
3.3 Food preparation surface washed (specify surface type)																	
3.4 Utensils rinsed																	
3.5 Utensils washed																	
3.6 Plates / bowls/ cups rinsed																	
3.7 Plates / bowls/cups washed																	
3.8 Child b ottle rinsed																	
3.9 Child b ottle cleaned																	
4. Food cooking																	Specify from section 3

Confidential: For Review Only

Direct observation

Household ID: _____

Activity	___ ____:00				____ ____:00				___ ____:00				____ ____:00				Pilot notes
	a	b	c	d	e	f	g	h	I	j	k	l	m	n	o	p	
	00	15	30	45	00	15	30	45	00	15	30	45	00	15	30	45	
																	Specify type in comments
4.1 Food fried (ASF / Fruit Veg / Other)																	
4.2 Food dried (ASF / Fruit Veg / other)																	
4.3 Food roasted (ASF / Fruit Veg / other)																	
4.4 Food heated in pot, no boiled (ASF/ Fruit Veg / other)																	
4.5 Food boiled (ASF / Fruit Veg/ other)																	
4.6 Food steamed (ASF / Fruit Veg / Other)																	
4.7 Food arrives processed (ASF / Fruit Veg / Other)																	
4.8 Food added raw / uncooked to dish (ASF / Fruit Veg / Other)																	
5. Food handling																	Specify from section 4
5.1 Cooked food served immediately to child																	
5.2 Cooked food left at room temp / open																	
5.3 Cooked food placed in cabinet / cupboard																	
5.4 Cooked food placed in fridge																	
5.5 Cooked food covered (loosely)																	
5.6 Cooked food covered (sealed)																	
6. Child feeding																	Specify from section 4
6.1 Child breastfed																	
6.2 Child given food that was stored																	
6.3 Child given milk																	
6.4 Child given water																	
6.5 Flies on food given to child																	
6.6 Child fed by caregivers hand																	
6.6 Child eating using child's hand																	
6.7 Child fed/eating using utensils (e.g. spoon)																	
7. Floor contact																	

Direct observation

Household ID: _____

Activity	___ ____:00				____ ____:00				___ ____:00				____ ____:00				Pilot notes
	a	b	c	d	e	f	g	h	I	j	k	l	m	n	o	p	
	00	15	30	45	00	15	30	45	00	15	30	45	00	15	30	45	
7.1 Child hand contact with floor																	
7.2 Child hand contact with dirt																	
7.3 Child eats dirt / soil																	
7.4 Child places objects from the floor in their mouths																	
7.5 Child places hands that have been in contact with the floor in mouth																	
8. Animal contact																	Write down the animal (chickens, ducks, other birds, goats, sheep, pigs, cow, dog, cat, other/specify)
8.1 Caretaker hand contact with animals																	
8.2 Caretaker hand contact with animal feces																	
8.3 Child hand contact with animals																	
8.4 Child hand contact with animal feces																	
9. Toilet use																	
9.1 Primary caretaker uses toilet																	
9.2 Primary caretaker goes for open defecation																	
10. Domestic cleaning																	
10.1 Caretakers washes clothes																	
10.2 Caretaker / other household member sweeps																	
10.3 Caretaker / other household members cleans floors (with disinfectant / soap)																	
10.3 Caretakers handles garbage																	
11. Handwashing / Facewashing																	
11.1 Caretaker rines hands – water only																	
11.2 Caretaker washes hands – soap used																	
11.3 Child's hands rinsed – water only																	
11.4 Child's hands washed – soap used																	
11.5 Child's face wiped or rinses																	
11.6 Child's face cleaned																	
11.7 Caretaker bathes																	

Confidential: For Review Only

Direct observation

Household ID: ______

Activity	___ ____:00				_____ _____:00				___ _____:00				_____ _____:00				Pilot notes
	a	b	c	d	e	f	g	h	I	j	k	l	m	n	o	p	
	00	15	30	45	00	15	30	45	00	15	30	45	00	15	30	45	
11.8 Child bathed																	

Confidential: For Review Only

Direct observation
Household ID: _____

Data log Table. Food Sample

HH ID: ________________________
Timepoint: 9 month/ 12 month/ 18 month
Date (DD/MMM/YY): ________________________
Collection time (24 hour clock, HH:MM): ---________________________

Collection period: During feeding ☐ Directly after feeding ☐ Before feeding ☐	
Food:	
Total sample weight	
Ingredients:	
Preparation method:	
Notes:	

Confidential: For Review Only

Direct observation
Household ID: _____

Data log Table. Water Sample

HH ID: ________________________
Timepoint: 9 month/ 12 month/ 18 month
Date (DD/MMM/YY): ________________________
Collection time (24 hour clock, HH:MM): ---________________________

Has the water been treated with chlorine/aquatabs or is from the municipal system?<br>Yes ☐ (collect water in a small 100 mL whirlpak bag with sodium thiosulfate tablets + collect further water in a 50mL centrifuge tube)<br>No ☐ (collect water in a large 250 mL whirlpak bag)	
Notes:	

Confidential: For Review Only

Direct observation
Household ID: _____

Data log Table. Surface Sample

Timepoint: 9 month/ 12 month/ 18 month
HH ID: _________________________
Date (DD/MMM/YY): _________________________
Collection time (24 hour clock, HH:MM): ---_________________________

Where is the child's food prepared [the surface where foods and utensils are placed during preparation and/or where foods cut up or otherwise processed in any way]?	
Notes:	

Confidential: For Review Only
